# Pulpectomy vs. Pulpotomy as Alternative Emergency Treatments for Symptomatic Irreversible Pulpitis—A Multicenter Comparative Randomised Clinical Trial on Patient Perceptions

**DOI:** 10.3390/clinpract13040082

**Published:** 2023-08-02

**Authors:** Guillem Esteve-Pardo, Pedro Barreiro-Gabeiras, Lino Esteve-Colomina

**Affiliations:** Group Aula Dental Avanzada, 03001 Alicante, Spain

**Keywords:** pulpitis, SIP, pulpectomy, pulpotomy, emergency treatment, pain management, patient-reported outcome measures

## Abstract

Aim: There needs to be more general agreement on the most effective treatment for the emergency patient with Symptomatic Irreversible Pulpitis (SIP). This equivalence randomised clinical study compared the clinical efficiency, as an urgent treatment, of pulpotomy (POT) and pulpectomy (PEC) in the permanent teeth with SIP. The primary outcome was pain management, and the secondary outcome was the patient’s perception of duration, comfort, and satisfaction. Material & Methods: 80 patients were blindly and randomly allocated into two equal parallel groups, the control group treated by PEC and the test group by POT. Data were collected through numerical rating scales (NRS) during the intervention and 6, 24, and 72 h post-op. Non-parametric tests were used to analyse the data. The Brunner-Longer models were adopted for longitudinal data and the analysis of variance (ANOVA)-type statistical was used. Results: The mean preoperative pain levels for the whole sample scored 5.8 ± 2.8 and significantly decreased to 2.1 ± 2.4 at 6 h, 1.5 ± 2.1 at 24 h, and 1.3 ± 2 at 72 h, without any differences between the groups. No significant differences were found in the patient’s perception of treatment discomfort or duration between the groups. Three days after the intervention, patient satisfaction was high, with 9.2 ± 1.7 and 9.1 ± 2 in the PEC and POT groups, respectively. Self-reported pain was the only variable penalising the patient’s final satisfaction. Conclusions: The current randomised control trial (RCT) showed that both pulpectomy and pulpotomy effectively eliminate pain and achieve high levels of patient satisfaction. Furthermore, the patient’s perceptions of the duration and discomfort of the two treatments were similar. Given that pulpotomy is a faster and more straightforward technique, it may be recommended as a viable and pragmatic option for treating emergency patients with symptomatic irreversible pulpitis.

## 1. Introduction

Pain emergencies are a significant problem in the dental office, and the cause is often endodontics. Although data on general prevalence are lacking, most general dentists would likely see daily deep caries or traumatic pulp exposures causing pulp affection [1].

Most endodontic emergencies come from Symptomatic Irreversible Pulpitis (SIP), a condition without a clinically precise definition beyond the lingering pain elicited by mild thermal stimuli [2] and the degree of spontaneity of its onset [3]. SIP implies that progressive pulpal inflammation has overpassed the threshold, in which tissue damage will not resolve without definitive treatments, a root canal, tooth extraction, or pulpotomy. SIP significantly impacts the patient’s perception of pain and quality of life [4,5,6], and removing inflamed pulpal tissues is mandatory to control the patient’s pain [1,7].

Traditionally, the preferred treatment option has been pulpectomy, even with complete instrumentation [6], and this approach continues [2,8]. The main goals of pulpectomy are to completely remove the vital pulp, to create enough space for irrigation, and to facilitate canal filling [9], all of these steps help maintain the initial asepsis of the root canal system [10].

Not long ago, the concept of minimally invasive endodontics was put forward [11,12] and since then, this approach has led several clinicians to return to pulpotomy as an alternative to root canal treatment and reported it as a valid option [13,14]. Pulpotomy consists of the complete removal of the coronal pulp, and its applications have even been extended to irreversible pulp conditions [15].

Because they are not scheduled, emergencies interrupt the dental team agenda and are often a source of stress for the operator, and sometimes even frustration for the patient, who feels the operator’s burden and rush [1,16]. When treating a patient with emergency SIP, the main objective is to avoid tooth extraction by eliminating the current pain as easily and comfortably as possible. Thus, the optimal technique for treating acute pulpal emergencies should combine clinical efficacy with the least possible postoperative pain, patient comfort, and practicality.

The long-term efficacy of complete root canal treatment is universally accepted as the gold standard in SIP treatment [17].

The improvement in the patient’s quality of life by root canal treatment has also been reported [18]. However, removing the coronal pulp is more straightforward than completing root canal treatment. Indeed, numerous researchers considered pulpotomy (POT) technically simpler, less time-consuming, and more cost-effective than pulpectomy (PEC) [15,16,19].

POT and PEC were previously compared in reducing postoperative pain in SIP patients, and both appeared to be equally valid [16,20,21,22]. However, studies have different methodologies, and there is no consensus on which of these two techniques could be the better option for SIP treatment. Indeed, a recent systematic review highlighted the current paucity of available evidence for basing clinical decisions on the existing direct comparative trials [3]. In fact, several surveys on dentists’ preferences for the treatment of deep carious exposures reflected a lack of consensus [3]. However, there is still little evidence of patients’ perceptions and experiences in the emergency treatment of SIP comparing total and partial pulp removal techniques.

This randomised clinical study aims to compare the clinical efficiency of POT and PEC for pain control in emergency SIP patients. It also compares the two therapies regarding the patient’s perception of treatment duration, comfort, and satisfaction.

## 2. Materials and Methods

### 2.1. Study Design

This multicenter clinical trial is a parallel-arm, single-blind, randomised, comparative study. It aims to compare two alternative techniques for managing symptomatic irreversible pulpits: POT and PEC. The study does not have an external funding source.

The study population was all patients consecutively attending two private dental clinics due to pain caused by SIP between 1 December 2020 and 31 July 2022.

Patients were randomly assigned to one of two parallel groups. The experimental group was treated with POT, and the control group was treated with PEC.

This randomised clinical trial has been designed according to the Consolidated Standards of Reporting Trials statement (CONSORT 2010).

Any patient presenting SIP, whatever its cause, in any tooth whose preservation was considered viable, was included in the study. Patients of any age, of either sex, in ASA I or II health status, and without contraindications to receiving an endodontic procedure under local anaesthesia were included. Haemorrhage after pulp exposure was considered an essential criterion for patient inclusion in the investigation.

Exclusion criteria before the procedure were allergy to non-steroidal anti-inflammatory drugs or local anaesthetics, pregnant or nursing women or with contraceptive treatment, psychotropic drug abuse, or medication that may alter painful perception taken 15 days before the intervention (e.g., anxiolytics), major mental disorders (dementia, psychosis), patients younger than 18 years, or patients uncooperative or with cognitive difficulties. The clinical exclusion criteria were a negative response to the cold pulp vitality test or absence of bleeding after pulp exposure (Table 1).

The random assignment of participants to one of the two treatment modalities, PEC or POT, was performed immediately before the intervention following a simple software-generated random number procedure using the “List Randomizer” application (https://www.random.org/lists, accessed on 15 November 2020). A block randomisation method was followed to allow equal allocation for both treatments per operator/centre. Before each procedure, the operator telephoned a secretary to find out the patient’s group assignment. The secretary did not participate in the investigation but managed the randomised list of interventions.

The participating patients were blinded to the type of treatment, and the operators were only aware of the treatment to be performed when the intervention. A third person, blinded to the clinical performances, was responsible for collecting and managing the data and writing the draft. Another statistician, unrelated to the investigators, analysed the data.

### 2.2. Sample-Size Calculation

Recent studies have reported a marked reduction in pain with both techniques. The medians of pain reduction described were −1.5 (range: −9 to 2) for complete PEC and −6 (range: −10 to 3) for POT [16].

The difference in medians (Δ = 4.5) estimates the shift of one distribution versus the other. The standard deviation in each group was calculated by the Wan-2014 method, with ±2.88 and ±3.40, for the PEC and POT groups, respectively.

These estimates show that Eren’s work’s effect size is large (d = 1.43). Therefore, a minimum of 80 patients (40 per group) is needed to detect significantly different pain reductions of 2 and 4.5 points (large effect with d = 0.8) in the groups with a statistical power of 90%. The statistical power calculations were performed by a statistician external to the investigation (GM, JL. ST Halley Statistics, Valencia, Spain).

### 2.3. Outcomes

In this study, the predictive variable was the intervention used to treat a tooth with SIP. The study’s primary outcome was the patient’s perception of pain after the intervention three days later. The secondary outcome was patient satisfaction three days later. Other secondary outcomes were the patient’s perception of the duration of treatment received and the degree of discomfort experienced. Data were measured from 0 to 10 on an 11-point NRS, verbalised by the patient.

Dropouts were defined as the patients who did not respond to all questionnaire responses up to three days after the intervention.

### 2.4. Patients

Once the patient was in the dental office, the diagnosis of SIP was confirmed by anamnesis, clinical, and radiographic examination. Firstly, the patient’s medical and dental history was recorded, particularly the pain characteristics. A visual examination of the tooth and the surrounding area was performed, including soft tissue palpation, periodontal evaluation, mobility, and percussion testing. Then, a periapical radiograph was taken to evaluate the periodontal and periapical structures.

To diagnose SIP, patients must present with severe, spontaneous, radiating pain that persists for 30 s or more after the removal of the stimulus, as defined by the recent American Association of Endodontists (AAE) and European Society of Endodontology (ESE) Consensus [23,24]. After verifying that the patient had SIP-compatible symptoms, a thermal pulp vitality test was performed to confirm the positive vitality status of the pulp. The test was made with a dry cotton pellet and cold spray (Endo-Frost, Roeko, Coltene Group, Altstätten, Swiss).

Only after a positive response to the vitality test will the patient be informed about his potential participation in the study. In case of verbal acceptance, the patient signed the Study Consent document.

The Ethics Committee of San Juan Hospital of the University Miguel Hernández, Alicante, Spain, approved the study with application nº 20/047, agreed on 25 November 2020. The study was registered on Clinicaltrials.gov with the identifier: NCT04654845. All interventions performed in this study followed the institutional and national research committee’s ethical standards, the 1964 Helsinki Declaration, and its later amendments or comparable ethical standards.

The operator then requested the type of procedure assigned to the particular patient by calling the appropriate telephone number. A secretary external to the investigation was the custodian of the randomised allocation list.

Once the procedure number was assigned and before anaesthesia was performed, the patient was asked the first three questions about their current pain, anxiety, and chewing pain in that area. The patients were blinded to the intervention they were about to receive.

### 2.5. Interventions

Anaesthesia was equally performed in both groups using 1.8 mL cartridges containing 4% articaine chlorhydrate (40 mg/mL) and 1:100,000 epinephrine (Ultracain, Normon Laboratories, Madrid, Spain). The anesthetic technique used on each tooth was appropriate for its location. Thus, the first-choice anesthetic techniques used were the inferior alveolar nerve block in the lower molars and second premolars, and the infiltrative techniques were used for the rest of the teeth.

Before starting the procedure, a new vitality test was performed to check the degree of anaesthesia achieved. Depending on the case, the operator determined whether or not to complete the anaesthesia with additional techniques, such as intraligamentary or intrapulpal anaesthesia.

For the experimental group (POT), the following clinical sequence was carried out:

- Isolation of the tooth with a rubber dam.
- Accessing to pulp chamber with a diamond bur.
- Confirmation of the presence of pulp bleeding.
- Removal of pulp tissue of the chamber with a round tungsten carbide bur.
- Use of cotton pellets soaked with sodium hypochlorite (NaOCl 5.25%).
- Waiting until the hemostasis is achieved.
- Placement of Teflon tape (PTFE) condensed on the chamber floor.
- Placement of a temporary restoration (Fermin, DETAX GmbH, Ettlingen, Germany).
- Occlusal reduction, verifying the absence of contact with 200μ articulating paper (Bausch Articulating Paper, NH, USA).

For the control group (PEC), the clinical sequence consisted of the following steps:

- Isolation of the tooth with a rubber dam.
- Accessing to pulp chamber with a diamond bur.
- Confirmation of the presence of pulp bleeding.
- Removal of pulp tissue of the chamber with a round tungsten carbide bur.
- Irrigation with sodium hypochlorite (NaCl at 5.25%) throughout the procedure, according to the operator’s preference.
- Determination of the working length for each canal using an Electronic Apex Locator (Root ZX mini, Morita, J. Morita Europe GmbH, Frankfurt, Germany) and a Pre-K file (EndoGal, Lugo, Spain).
- Root canal preparation using rotary cutting instruments with a reciprocating system (EndoGal, Lugo, Spain) up to WL and up to a minimum apical diameter of #25.
- Placement of Teflon tape (PTFE) condensed on the chamber floor. Calcium hydroxide was not used so as not to create differences between the two groups due to its anti-inflammatory effects.
- Placement of a temporary restoration (Fermin, DETAX GmbH, Ettlingen, Germany).
- Occlusal reduction, verifying the absence of contact with 200 μ articulating paper (Bausch Articulating Paper, NH, USA).

In both groups, patients were advised to avoid chewing on the treated side to prevent coronal fractures of the tooth under treatment.

Before leaving the office, the preferred form of contact, text message or phone call, was agreed by the patient to obtain the investigated data further.

### 2.6. Data Collection and Management

The operator recorded the patients’ perception data in an ‘Intervention Document’ form assigned to each procedure/patient. At the end of the treatment, the operator filled out the form with the procedure data.

To better understand how patients should describe their perceived degree of pain, the NSR included in the “Intervention Document” was explained to them in the preoperative phase. The numerical pain perception scale scored pain from 0 to 10, with 0: no pain, 1–3: mild, 4–6: moderate, 7–9: severe, and 10: the worst pain imaginable. Patients were asked to state the number that best represented the perceived duration of the pain they were experiencing at the time of assessment. Patient responses to the NSR were collected on the following four occasions:

- Before the intervention: the patient’s perception of current pain, anxiety, and pain sensation when chewing on the diseased side.
- Immediately after the intervention: the patient’s perception of the duration of treatment and the degree of discomfort during treatment.
- At the time intervals after the intervention: the patient’s perceived pain at 6, 24, and 72 h.
- The patient’s global satisfaction with the treatment 72 h after the procedure.

The patient’s perception at the postoperative intervals was obtained by phone calls or messages. The patient must indicate the NSR score in the same way as they did when in physical presence in pre- and immediate postoperative pain assessment.

The operators collected the following procedure data:

- Age.
- Gender.
- The number of teeth involved (FDI World Dental Federation notation).
- The cause of SIP (caries, previous restoration, cracks, periodontal affection, trauma, cusp fractures, and occlusal or cervical wear).
- The presence of primary acute apical periodontitis (AAP) with a percussion test before the intervention, in terms of Yes or No.
- Whether preoperative non-steroidal anti-inflammatory drugs (NSAIDs) had been taken by the patient, Yes or No.
- The time elapsed from pulp exposure to the end of the intervention in minutes.
- The postoperative chewing pain in terms of Yes or No.

The operators ticked the relevant boxes on the ’Intervention Form´ and filled in the five NSR scores submitted by the patient 6, 24, and 72 h after the intervention; then, a third investigator, blinded to the clinical cases, transferred data to the ´Data Table.´ Once this table with the whole sample of 80 interventions/patients was completed, it was statistically analyzed.

### 2.7. Statistical Analysis

The Mann-Whitney test was used to compare the rating distributions in the two groups, and the Wilcoxon test to compare the pain distributions between different follow-up moments. Brunner-Langer’s non-parametric longitudinal data models were used to analyse the evolution of the pain level throughout the study. A between-subjects factor (the group) and a within-subjects factor (moment of assessment) were included. The analysis of variance (ANOVA)-type statistic (ATS) was calculated to evaluate the main effects and interaction.

The choice of this methodology was justified by the ordinal nature of the data and its lack of normal distribution. This model is a non-parametric ANOVA with repeated measures and maximum statistical power.

The Mann-Whitney and Wilcoxon tests with Bonferroni correction were used for multiple comparisons between groups at different measurement moments or between moments within each group. Spearman’s non-parametric correlation was used to assess the degree of non-linear association between different questionnaire ratings.

A multiple linear regression model was used to analyze the outcome satisfaction related to other variables. The input method for the variables in the model was stepwise. The significance level used in the analyses was 5% (α = 0.05).

## 3. Results

**Figure 1:**
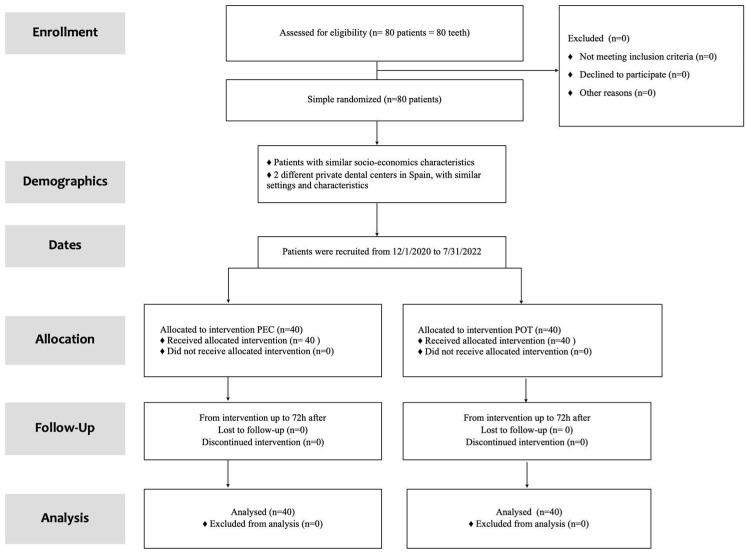
Flow-chart. PEC: Pulpectomy; POT: Pulpotomy.

Eighty patients (40 men and 40 women) with a mean age of 50.7 years (19 to 81, ±15.2) were included in the study between 1 December 2020 and 31 July 2022. The study ended when the calculated sample size of 80 patients was reached. All patients in the study reported their degree of pain and satisfaction at all necessary moments (up to 3 days after the intervention), so there were no dropouts (Figure 1).

Table 2 shows the demographic characteristics, tooth type, cause of pulp inflammation, presence of AAP, and previous NSAID use in the two intervention groups. Both groups were homogeneous since the differences were statistically insignificant. Half of the patients had deep caries as a cause of pulpitis, 28.8% had a previous restoration, 7.5% had a fissure, and the rest of the sample had other pathologies such as periodontal, trauma, cusp fractures, or occlusal or cervical wear.

Table 3 shows the mean NRS scores of the patient-perceived variables for both groups. Before treatment, the mean pain experienced in the whole sample was 5.8 ± 2.8, 6 ± 2.6 in the control PEC group and 5.6 ± 3.1 in the experimental POT group. The mean pain reduction from pre-treatment to 3 days after treatment was 4.7 ± 3.2 in the PEC group and 4.3 ± 3.8 in the POT group, with no statistical difference between them.

**Figure 2:**
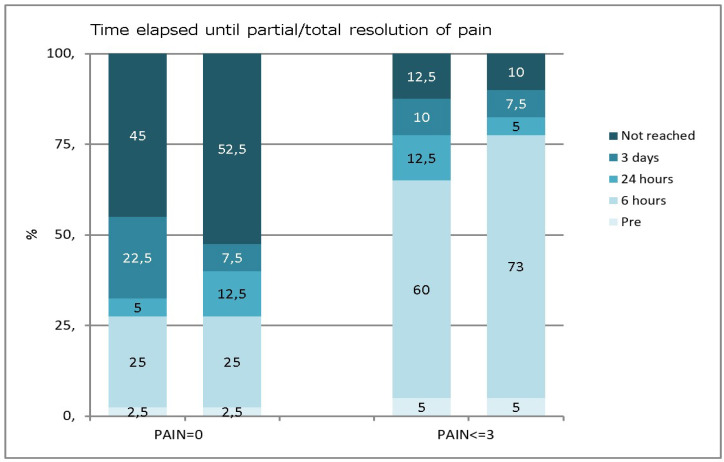
Percentage of patients reaching zero and low pain levels at different follow-up times in the two groups. The bar graph on the left corresponds to patients who reached a pain level of 0, and the bar graph on the right corresponds to patients who reached a low level of pain. The bars are for PEC (left) and POT (right). The numbers represent the percentages. PEC: Pulpectomy; POT: Pulpotomy.

Table 4 shows that the most significant reduction occurred 6 h after treatment for both groups. The pain continued dropping until 24 h, and half of the samples achieved the complete absence of pain after 3 days, with no differences between the two groups (Figure 2).

**Figure 3:**
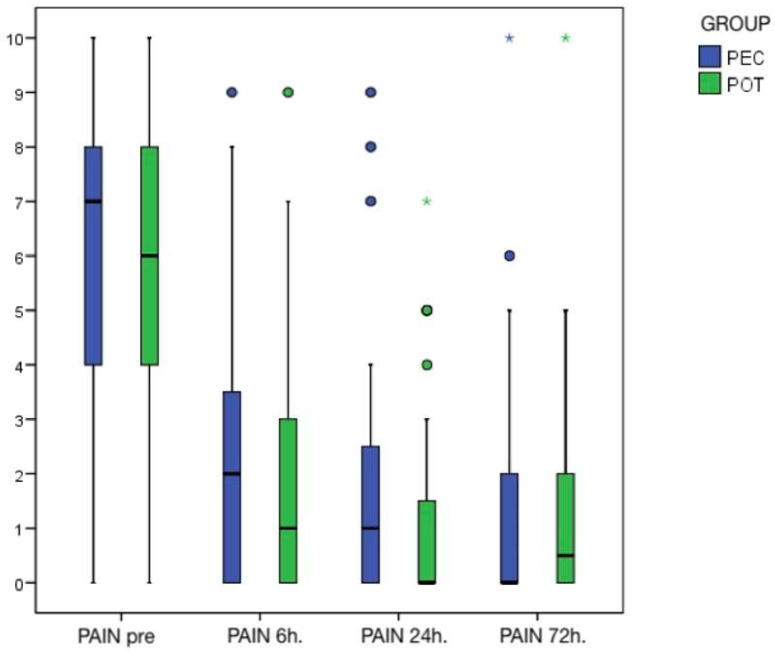
Boxplot showing an identical pattern in the decrease of pain in both techniques. The outliers specific to each case are represented by dots and astherisks. PEC: Pulpectomy; POT: Pulpotomy.

The patterns of pain decrease during the follow-up time can be seen in Figure 3. They were parallel between the two groups with some outliers.

Sixty percent of the patients required supplementary anaesthesia, with a homogeneous distribution of 24 patients in each group. No statistically significant association was found between the need for supplementary anaesthesia and the evolution of pain perception or the final patient satisfaction.

Patients reported a mean score of anxiety before treatment of 3.2 ± 3.4, and a weak-moderate relation (r = 0.39) was found with preoperative pain perception (*p* < 0.001), especially in women who seemed to feel more anxiety before treatment (*p* = 0.024). Fifty patients (62.5%) had previous experience with endodontic treatment, but the patient’s previous experience did not condition the magnitude of the perceived pain or the time it takes to subside (*p* = 0.286).

Thirty-seven patients had taken NSAIDs before the intervention, with 17 in the PEC group and 20 in the POT group. No statistical correlation was found between previous NSAID intake and the evolution of post-treatment pain (*p* = 0.706). Most patients (56 in total and 28 in each group) did not need painkillers after treatment. No significant differences were found between the two treatments and the need or not to take subsequent medication (*p* = 0.099).

Patients reported a mean score of chewing pain before treatment of 6.5 ± 3.3. Thirty-three patients (41.3% of the total samples) presented AAP concomitant with SIP. Twenty patients were treated with PEC and 13 with POT. Before the intervention, pain in patients with AAP was higher (*p* = 0.052); therefore, the pain reduction was more significant than when no AAP symptoms were present previous to the intervention (*p* = 0.033). Regardless of the presence or absence of AAP, the pain evolved similarly after treatments in both groups (*p* = 0.614). Twelve patients (15%) still had postoperative chewing pain, but this was not statistically related to the type of intervention (*p* = 0.201).

**Figure 4:**
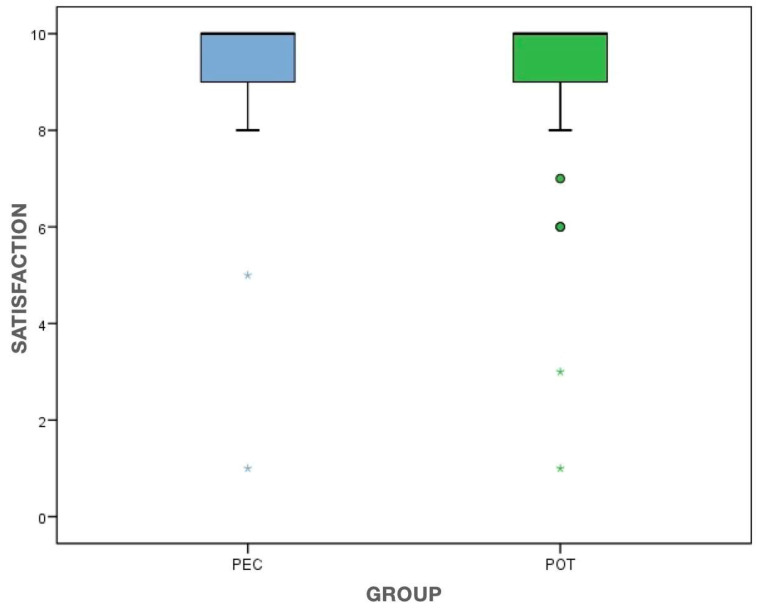
Boxplot showing an identical pattern of patient satisfaction three days after treatment. The outliers are represented by dots and astherisks PEC: Pulpectomy; POT: Pulpotomy.

Three days after the treatment, the mean patient satisfaction scored 9.2 ± 1.7 and 9.1 ± 2.0 in the PEC and POT groups, respectively. The homogeneity between them (*p* = 0.848, MW) can be seen in Figure 4.

Among all the variables, pain perception was the most penalised for the patient’s final satisfaction and, especially the pain level on the third day, the same day they had to communicate their level of satisfaction (Table 5).

The mean intervention time in minutes, from pulp exposure to finalisation of the treatment, was 13.4 ± 6.1 for the control PEC group and 7.4 ± 3.0 for the test POT group. In both groups, the longer the pulp was exposed, the longer it took the patient to reach pain levels lower than score 3 (*r* = 0.23; *p* = 0.044).

**Figure 5:**
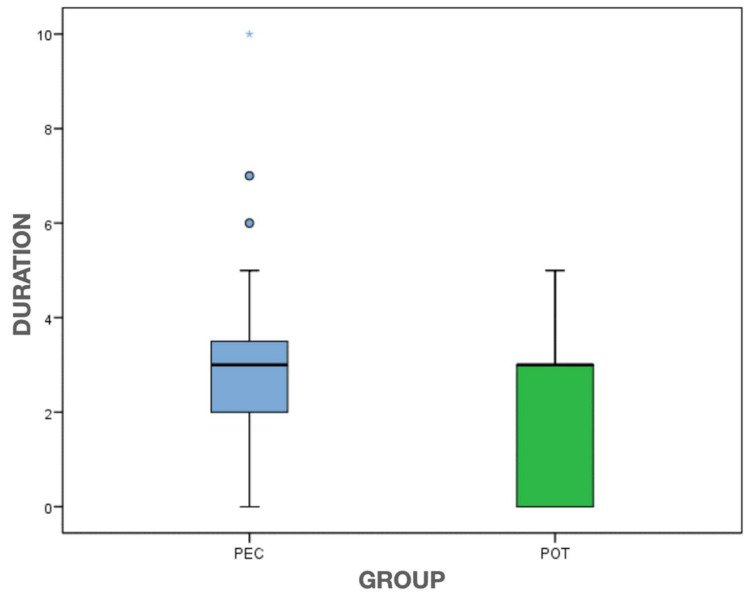
Boxplot showing the differences between the two groups regarding the patient’s perception of the duration of the treatments. The outliers are represented by dots and astherisks PEC: Pulpectomy; POT: Pulpotomy.

Patients perceived both interventions as short duration with a median of 3 (1.0–3.0) for all the samples. However, Figure 5 shows that the PEC group scored significantly higher on the perception of duration as moderate-long than the POT group (*p* = 0.021, MW). A weak-moderate correlation (*r* = 0.4) was also found between the patient’s perceived duration and the time taken from pulp exposure to the end of treatment in both groups, especially in the PEC group (*p* = 0.034). 

The mean level of discomfort perceived by the patients was 1.5 ± 1.9 with no statistically significant differences between the two groups. All teeth received a complete root canal treatment at a second appointment, irrespective of the group they belonged to during the investigation.

## 4. Discussion

The present study aimed to test the null hypothesis that both of the interventions investigated were equally effective in eliminating pain in emergency SIP patients. Data collection was set up to three days post-treatment to capture the most immediate decrease in severe pain. The patients’ satisfaction and perceptions of the duration and discomfort of both interventions were also studied.

According to the results, both interventions appeared to be equally effective in eliminating pain in emergency SIP patients. Regardless of the procedure, patients benefited from a significant reduction in pain within 6 h of the intervention, with no statistical differences between the two groups. Since the homogeneity of the test and control groups was statistically confirmed, due to the lack of differences in the pain perceived by the patients after receiving one type of treatment or another this study failed to reject the null hypothesis. The atypical scores, outliers in Figure 3, obtained three days after the intervention were due, in one case, to mucosal pain caused by intraligamentary anaesthesia and, in another case, to the maintenance of pulp inflammation. In the latter, a new endodontic treatment had to be performed four days after the initial POT.

Although the patient’s satisfaction with the treatment depends on factors beyond the mere technical procedure, such as duration, comfort, cost, or personal rapport, the treatment outcome is satisfaction’s first and foremost cause. In this case, the satisfactory outcome meant pain dissipation. When this outcome was not achieved in either group, higher pain scores correlated significantly with lower patient satisfaction. This study found that self-reported satisfaction with treatment was similarly high in both groups.

Even though POT can be performed faster than PEC by about half of the intervention time, patients scored the perceived duration and discomfort of both interventions similarly.

In this RCT, SIP was diagnosed by pain anamnesis and cold vitality testing. The precise diagnosis of the pulp status is challenging for clinicians since there is no precise correlation between the symptoms and the pulp tissue histopathological status severity [25,26]. Thus, a histological-based pulp examination would be needed to allow a precise pulp status diagnosis. Moreover, the condition of the pulp will determine the type of treatment required. In endodontic emergencies, the most common situations are pulp inflammation and pulp infection [27]. Several diagnostic tests are available to inform the clinician about the pulp status in painful conditions [2], but some of them are expensive and technically complex [28]. However, the cold vitality test has shown a sufficiently high degree of sensitivity and specificity, second only to the pulse oximeter [29]. The electric pulp test (EPT) could also be added to the diagnostic tools since some authors concluded that a combination of tests and clinical symptoms might be the best way to approach the actual state of the pulp [28]. However, an EPT device is only sometimes used in our environment. This study emphasised practicality, focusing on the daily setting including generalists. Therefore, the cold test was preferred, and the diagnosis was based on the cold vitality test and the anamnesis of pain. Besides, bleeding after pulp exposure was an essential inclusion criterion in the intervention, thus avoiding false positive treatments.

Some studies considered the type and intensity of pulp bleeding as indicative of the pulp status and its reversibility [25]. In the present multicenter study, this sign was discarded as a diagnostic indicator to avoid introducing observer bias. Therefore, some of the treated pulps could not have been in such an advanced state of irreversibility. This circumstance would explain why some patients in the POT group still showed signs of maintained pulp vitality at the second intervention when, outside the investigation, the complete root canal treatment was performed, even without any pulp capping. The potential underestimation of the state of the pulp also refers to the inability of the reversible/irreversible classification to deal with the different pulp situations of symptomatic pulpitis [30]. Indeed, the very dichotomous classification of reversible/irreversible pulpitis has been called into question, as it does not fit the continuum of the pulpal inflammatory process observed in the clinic [12,31]. Some authors proposed the classification (mild/moderate/severe pulpitis) as more discriminating of the state of the pulp and its healing capacity [12,32].

Although both scales are validated and widely used [33], in this study, the NRS scale was selected due to its simplicity, ease of use, better responsiveness, and good applicability compared to the visual analog scale (VAS). NRS was proven to be equivalent to VAS in pain assessment [34]. NRS was considered optimal for evaluating acute pain and has been used even in children [35]. Its potential drawback is response variability, which is inherent to pain assessment’s subjective nature.

Pain in chewing is the classic symptom of the AAP patient. In this study, patients who had SIP and AAP experienced higher preoperative pain and subsequent higher reduction of pain scores than those without AAP. Some authors have found a positive relationship between occlusal reduction and pain decrease three days after endodontic treatment [32]. In a study similar to the present RCT, chewing pain lessened earlier in patients treated with POT than in patients who received PEC or partial PEC [16]. However, the treated teeth did not undergo occlusal reduction. In this study, occlusal surfaces of treated teeth were reduced to the absence of contacts, with a 200μ articulated paper in both groups. Occlusal reduction may have influenced the degree of pain decrease without significant differences between the groups. Thus, it is possible to rule out the potential confusion caused by the presence of the AAP variable in analysing the differences between the two groups.

In the same randomised study, the patients’ preoperative pain levels averaged around eight points on a VAS scale [16]. However, in our study, the mean pre-treatment pain expressed was 5.8 ± 2.8 points, representing relatively moderate pain. Several factors might have influenced this difference. NSAID intake could be one of them, but no significant relationship was found with pre-treatment pain. Anxiety was related to pain perception [36]. However, in our study, patients reported low mean levels of anxiety. More than half of the patients had no anxiety, which may explain our sample’s moderate average scores of pre-treatment pain. Previous patient experience with the procedure has also been a factor in pre-treatment pain relief [37]. In our sample, more than half of the patients had already experienced the type of treatment, but no statistical significance was found for this factor concerning pre-treatment pain scores. Differences in SIP diagnosis are also possible. Therefore, some pulpitis in our sample could have been treated in a less advanced inflammatory process.

POT is a faster technique than PEC [38,39]. Some authors have reported POT durations up to almost five times less than PEC [16]. In this study, the difference is up to almost half the intervention time. Several factors can influence the duration of treatment, including the operator or the tooth being treated. Figures obtained in a multicenter study could therefore be more representative than when a single operator intervenes. As for the type of tooth, in this study, 97% of the teeth were multi-rooted. The number of anterior teeth was negligible (2.6%) and cannot have influenced the different treatment duration between the groups. In our study, the duration of the intervention was set as the time from pulp exposure to the end of treatment, similar to Eren et al. [16], and the time from anaesthesia to pulp exposure was disregarded. Thus, influential variables such as the time of the anaesthetic effect, whether pre-endodontic reconstruction has been performed on the affected tooth, or the time it took for the operator to attend to the patient once anaesthetised, have been discarded.

On the other hand, the patient’s perception of the duration of the treatment is an independent variable. We find no differences between the groups if we look exclusively at the patient-centred results. In the end, a few minutes seem unimportant to the patient. Similarly, no differences were found in the patient’s perception of discomfort between the procedures, even if one was shorter than the other. It has already been shown in other studies that root canal treatment is not as poorly perceived as one might think in terms of its lack of comfort [40]. Nevertheless, POT seems to have three practical advantages from the clinician’s perspective. Firstly, POT maintains the option of selecting the case for Vital Pulp Therapy (VPT). Also, the clinician can perform a more straightforward and faster treatment with similar short-term clinical results. Finally, with a less time-consuming technique, a better organisation of the clinician’s work and schedule can be achieved. [15,16].

When talking about efficacy, we think of the ability of a procedure, under ideal conditions, to achieve the desired results, eliminating pain and obtaining patient satisfaction. However, effectiveness means producing the desired effect in real-world settings, with less controlled factors and a wider range of circumstances. In this regard, clinicians prefer the most effective procedure, as simple and fast as possible, especially when dealing with an emergency with no previous appointment. Since POT is more straightforward and shorter than PEC, it could be considered a more effective procedure for treating SIP emergencies. POT is even considered by various public health systems as the treatment of choice for irreversible pulpitis in adults due to its better cost-benefit ratio [11]. However, in this regard, further investigation is needed to compare the longer-term outcomes of both alternative treatment modalities for SIP [36].

Moreover, a POT is considered an effective and less invasive treatment in the VPT approaches [12,19,32,38,39,41]. Treating a SIP initially with a POT leaves the possibility of performing a PTV in a second intervention, depending on the pulp reaction and the patient’s response to emergency treatment, thus avoiding over-treatment of pulps potentially mistaken for irreversible. It is, therefore, a matter of making more definitive treatment decisions in a pre-planned situation not conditioned by urgency.

Regarding study limitations, since the study findings are restricted to large size differences, it cannot be concluded that POT is the “technique of first choice” without having scientifically weighed the cost, availability, and accessibility of both treatments as the relevant factors in choosing the best option. Additionally, the study only looks at short-term outcomes and does not address the seven-day success rate of the two treatment techniques following the standard method [42]. Depending on their preferences, the operators diagnosed and performed the treatment, potentially adding some bias to the result. On the other hand, the thermal test cannot be considered 100% reliable, and a pulp oximeter could have refined the SIP diagnosis. Lastly, the study did not control for influencing variables such as the individual patients’ pain tolerance or expectation.

However, the study provides valuable information on the comparable efficacy of POT and PEC and the superior effectiveness of POT for the emergency treatment of irreversible pulpitis.

## 5. Conclusions

The current randomised controlled trial compared two endodontic treatment techniques for emergency patients with symptomatic irreversible pulpitis. The results demonstrated that both pulpectomy and pulpotomy effectively eliminate pain and achieve high levels of patient satisfaction. Furthermore, the patient’s perceptions of the duration and discomfort of the two treatments were comparable.

Given that pulpotomy is a faster and more straightforward technique, it may be recommended as a viable and pragmatic option for treating emergency patients with symptomatic irreversible pulpitis.

## Figures and Tables

**Table 1:** Exclusion/inclusion criteria for medical and clinical conditions. NSAIDs: Non-Steroidal Anti-Inflammatory Drugs; ASA: American Society of Anesthesiology.

Type of Criteria	Medical	Clinical
Inclusion	ASA I/II Without contraindication for endodoncias procedure under local anesthesia	Acute Irreversible pulpitis Cold vitality test+ Pulp hemorrhage after exposure
Exclusion	Allergy NSAIDs Allergy to local anesthetics Pregnant, nursing, or with contraceptive treatment Psychotropic drugs and pathological mental states (dementia, psychosis) Young patients (<18 years) Uncooperative patients or cognitive difficulties	Cold Vitality test− No bleeding after pulp exposure

**Table 2:** Demographic characteristics, tooth type, cause of pulp inflammation, presence of AAP and previous NSAID use; distribution between groups, and statistical analysis result with the *p*-value. NSAID: Non-Steroidal Anti-Inflammatory Drug; AAP: Acute Apical Periodontitis; PEC: Pulpectomy. MW: Mann-Whitney test. Chi2: Chi square test.

	Variable	PEC-N (%)	POT-N (%)	*p* Value (Test)
Gender	Men	20 (50%)	20 (50%)	1.000 (Chi2)
	Women	20 (50%)	20 (50%)	
Age	Years mean (SD=)	50.8 (SD = 15.4)	50.6 (SD = 15.2)	0.985 (MW)
Tooth Type Group	Molar	28 (70%)	25 (62.5%)	0.690 (Chi2)
	Premolar	12 (30%)	13 (32.5%)	
	Anterior	0 (0%)	2 (5%)	
Cause of pulpitis	Decay	19 (47.5%)	21 (52.5%)	0.760 (Chi2)
	Restoration	13 (32.5%)	10 (25%)	
	Others	8 (20%)	9 (22.5%)	
	Previous AAP present	20 (50%)	13 (32.5%)	0.112 (Chi2)
	Previous NSAID intake	17 (42.5%)	20 (50%)	0.501 (Chi2)

**Table 3:** All the variables as the patients give their opinions in the form of a numerical assessment between groups and the result of the statistical significance test. NSR: Numerical Score Rate; SD: Standard Deviation; PEC: Pulpectomy; POT: Pulpotomy. MW: Mann-Whitney test. Chi2: Chi square test. ATS: ANOVA-type statistic (Brunner-Langer model). * *p* < 0.001.

	NSR Scores from 0 to 10 (Mean SD=); Median		
Variable	PEC	POT	*p* Value (Test)
Previous pain	6.0 (SD = 2.6); 7.0	5.6 (SD = 3.1); 6.0	0.763 (MW)
Previous anxiety	3.2 (SD = 3.2); 2.0	3.3 (SD = 3.5); 2.5	0.908 (MW)
Previous chewing pain	7.2 (SD = 2.7); 8.0	5.8 (SD = 3.6); 6.5	0.147 (MW)
Perceived duration	3.1 (SD = 1.9); 3.0	2.0 (SD = 1.6); 3.0	0.021 * (MW)
Degree of discomfort	1.5 (SD = 2.0); 0.0	1.6 (SD = 1.8); 1.0	0.453 (MW)
Pain after 6 h	2.2 (SD = 2.6); 2.0	2.0 (SD = 2.3); 1.0	
Pain after 24 h	1.8 (SD = 2.3); 1.0	1.3 (SD = 2.0); 0.0	
Pain after 3 days	1.3 (SD = 2.1); 0.0	1.3 (SD = 2.0); 0.5	0.522 (ATS)
Degree of satisfaction	9.2 (SD = 1.7); 10.0	9.1 (SD = 2.0); 10.0	0.848 (MW)

**Table 4:** Degree of pain between specific moments in each group: Wilcoxon test results with Bonferroni correction. ** *p* < 0.01; *** *p* < 0.001.

	PEC	POT
Pre vs. 6 h	<0.001 ***	<0.001 ***
6 h vs. 24 h	0.849	0.003 **
24 h vs. 3 d	0.354	1.000

**Table 5:** Degree of satisfaction according to the degree of pain 3 days after treatment: results of the simple linear regression model with a stepwise entry of the variables. Beta coefficients, standard error (SE), and 95% confidence intervals.) The final pain rating is the most important variable in explaining the final satisfaction. *** *p* < 0.001.

	Beta	SE	CI 95%	*p*-Value
Constant	9.76	0.20	9.36 10.2	<0.001 ***
Pain 3 d	−0.50	0.09	−0.67 −0.33	<0.001 ***

## Data Availability

All the data presented in this study are available in Supplementary File S1.

## Supplementary Materials

The following supporting information can be downloaded at: https://www.mdpi.com/article/10.3390/clinpract13040082/s1.
