# Supplementary material for: Pulpectomy vs. Pulpotomy as Alternative Emergency Treatments for Symptomatic Irreversible Pulpitis—A Multicenter Comparative Randomised Clinical Trial on Patient Perceptions
Source: Clin Pract. 2023 Aug 2;13(4):898–913. doi: 10.3390/clinpract13040082 (PMC10453127; doi:10.3390/clinpract13040082)
Supplement: Supplementary file 1 [file clinpract-13-00082-s001.zip › clinpract-2429426-supplementary.pdf]

## T0.- GRUPO

| | N | % |
|---|---|---|
| Total | 80 | 100,0 % |
| PEC | 40 | 50,0 % |
| POT | 40 | 50,0 % |

## T1.- SEXO según GRUPO

| | GRUPO | | | | | |
|---|---|---|---|---|---|---|
| | Total | | PEC | | POT | |
| | N | % | N | % | N | % |
| Total | 80 | 100,0 % | 40 | 100,0 % | 40 | 100,0 % |
| Hombre | 40 | 50,0 % | 20 | 50,0 % | 20 | 50,0 % |
| Mujer | 40 | 50,0 % | 20 | 50,0 % | 20 | 50,0 % |

## T2.- EDAD (años) según GRUPO

| | GRUPO | | |
|---|---|---|---|
| | Total | PEC | POT |
| N | 80 | 40 | 40 |
| Media | 50,7 | 50,8 | 50,6 |
| Desviación típica | 15,2 | 15,4 | 15,2 |
| Mínimo | 19,0 | 19,0 | 21,0 |
| Máximo | 81,0 | 81,0 | 80,0 |
| Percentil 25 | 40,5 | 41,5 | 40,0 |
| Mediana | 49,5 | 49,0 | 52,0 |
| Percentil 75 | 62,5 | 63,0 | 60,5 |

## T3.- DIENTE según GRUPO

| | GRUPO | | | | | |
|---|---|---|---|---|---|---|
| | Total | | PEC | | POT | |
| | N | % | N | % | N | % |
| Total | 80 | 100,0 % | 40 | 100,0 % | 40 | 100,0 % |

| | | | | | | |
|---|---|---|---|---|---|---|
| 12 | 1 | 1,3 % | 0 | 0,0 % | 1 | 2,5 % |
| 14 | 2 | 2,5 % | 2 | 5,0 % | 0 | 0,0 % |
| 15 | 4 | 5,0 % | 0 | 0,0 % | 4 | 10,0 % |
| 16 | 2 | 2,5 % | 1 | 2,5 % | 1 | 2,5 % |
| 17 | 4 | 5,0 % | 2 | 5,0 % | 2 | 5,0 % |
| 18 | 1 | 1,3 % | 1 | 2,5 % | 0 | 0,0 % |
| 24 | 3 | 3,8 % | 0 | 0,0 % | 3 | 7,5 % |
| 25 | 5 | 6,3 % | 5 | 12,5 % | 0 | 0,0 % |
| 26 | 6 | 7,5 % | 2 | 5,0 % | 4 | 10,0 % |
| 27 | 13 | 16,3 % | 8 | 20,0 % | 5 | 12,5 % |
| 33 | 1 | 1,3 % | 0 | 0,0 % | 1 | 2,5 % |
| 34 | 1 | 1,3 % | 1 | 2,5 % | 0 | 0,0 % |
| 35 | 3 | 3,8 % | 1 | 2,5 % | 2 | 5,0 % |
| 36 | 2 | 2,5 % | 1 | 2,5 % | 1 | 2,5 % |
| 37 | 8 | 10,0 % | 6 | 15,0 % | 2 | 5,0 % |
| 38 | 1 | 1,3 % | 0 | 0,0 % | 1 | 2,5 % |
| 44 | 2 | 2,5 % | 1 | 2,5 % | 1 | 2,5 % |
| 45 | 5 | 6,3 % | 2 | 5,0 % | 3 | 7,5 % |
| 46 | 8 | 10,0 % | 5 | 12,5 % | 3 | 7,5 % |
| 47 | 6 | 7,5 % | 1 | 2,5 % | 5 | 12,5 % |
| 48 | 2 | 2,5 % | 1 | 2,5 % | 1 | 2,5 % |

T4.- TIPO DIENTE según GRUPO

| | GRUPO | | | | | |
|---|---|---|---|---|---|---|
| | Total | | PEC | | POT | |
| | N | % | N | % | N | % |
| Total | 80 | 100,0 % | 40 | 100,0 % | 40 | 100,0 % |
| Incisivos | 1 | 1,3 % | 0 | 0,0 % | 1 | 2,5 % |
| Caninos | 1 | 1,3 % | 0 | 0,0 % | 1 | 2,5 % |
| Premolares | 25 | 31,3 % | 12 | 30,0 % | 13 | 32,5 % |
| Molares | 53 | 66,3 % | 28 | 70,0 % | 25 | 62,5 % |

## T5.- ARCADA según GRUPO

| | GRUPO | | | | | |
|---|---|---|---|---|---|---|
| | Total | | PEC | | POT | |
| | N | % | N | % | N | % |
| Total | 80 | 100,0 % | 40 | 100,0 % | 40 | 100,0 % |
| Maxilar | 41 | 51,2 % | 21 | 52,5 % | 20 | 50,0 % |
| Mandibular | 39 | 48,8 % | 19 | 47,5 % | 20 | 50,0 % |

## T6.- CAUSA PULPITIS según GRUPO

| | GRUPO | | | | | |
|---|---|---|---|---|---|---|
| | Total | | PEC | | POT | |
| | N | % | N | % | N | % |
| Total | 80 | 100,0 % | 40 | 100,0 % | 40 | 100,0 % |
| Caries | 40 | 50,0 % | 19 | 47,5 % | 21 | 52,5 % |
| Obturación | 23 | 28,7 % | 13 | 32,5 % | 10 | 25,0 % |
| Periodontal | 3 | 3,8 % | 1 | 2,5 % | 2 | 5,0 % |
| Fisura | 6 | 7,5 % | 5 | 12,5 % | 1 | 2,5 % |
| Fractura cuspídea | 2 | 2,5 % | 1 | 2,5 % | 1 | 2,5 % |
| Desgaste cervical | 3 | 3,8 % | 1 | 2,5 % | 2 | 5,0 % |
| TAL | 3 | 3,8 % | 0 | 0,0 % | 3 | 7,5 % |

## T7.- PAA según GRUPO

| | GRUPO | | | | | |
|---|---|---|---|---|---|---|
| | Total | | PEC | | POT | |
| | N | % | N | % | N | % |
| Total | 80 | 100,0 % | 40 | 100,0 % | 40 | 100,0 % |
| No | 47 | 58,8 % | 20 | 50,0 % | 27 | 67,5 % |
| Sí | 33 | 41,3 % | 20 | 50,0 % | 13 | 32,5 % |

## T8.- AINES PRE según GRUPO

| | GRUPO |
|---|---|

| | Total | | PEC | | POT | |
|---|---|---|---|---|---|---|
| | N | % | N | % | N | % |
| Total | 80 | 100,0 % | 40 | 100,0 % | 40 | 100,0 % |
| No | 43 | 53,8 % | 23 | 57,5 % | 20 | 50,0 % |
| Sí | 37 | 46,3 % | 17 | 42,5 % | 20 | 50,0 % |

T9.- EXPERIENCIA PREVIA según GRUPO

| | GRUPO | | | | | |
|---|---|---|---|---|---|---|
| | Total | | PEC | | POT | |
| | N | % | N | % | N | % |
| Total | 80 | 100,0 % | 40 | 100,0 % | 40 | 100,0 % |
| No | 23 | 28,7 % | 14 | 35,0 % | 9 | 22,5 % |
| Sí | 50 | 62,5 % | 24 | 60,0 % | 26 | 65,0 % |
| NS | 7 | 8,8 % | 2 | 5,0 % | 5 | 12,5 % |

T10.- PRIMERA VISITA según GRUPO

| | GRUPO | | | | | |
|---|---|---|---|---|---|---|
| | Total | | PEC | | POT | |
| | N | % | N | % | N | % |
| Total | 80 | 100,0 % | 40 | 100,0 % | 40 | 100,0 % |
| No | 57 | 71,3 % | 31 | 77,5 % | 26 | 65,0 % |
| Sí | 23 | 28,7 % | 9 | 22,5 % | 14 | 35,0 % |

T11.- ANESTESIA según GRUPO

| | GRUPO | | | | | |
|---|---|---|---|---|---|---|
| | Total | | PEC | | POT | |
| | N | % | N | % | N | % |
| Total | 80 | 100,0 % | 40 | 100,0 % | 40 | 100,0 % |
| Infiltrativa | 45 | 56,3 % | 23 | 57,5 % | 22 | 55,0 % |
| Troncular | 35 | 43,8 % | 17 | 42,5 % | 18 | 45,0 % |

## T12.- ANESTESIA COMPLEMENTARIA según GRUPO

| | | GRUPO | | | | | |
|---|---|---|---|---|---|---|---|
| | | Total | | PEC | | POT | |
| | | N | % | N | % | N | % |
| INTRALIGAMENTOSA | Total | 80 | 100,0 % | 40 | 100,0 % | 40 | 100,0 % |
| | No | 43 | 53,8 % | 23 | 57,5 % | 20 | 50,0 % |
| | Sí | 37 | 46,3 % | 17 | 42,5 % | 20 | 50,0 % |
| INTRAPULPAR | Total | 80 | 100,0 % | 40 | 100,0 % | 40 | 100,0 % |
| | No | 55 | 68,8 % | 27 | 67,5 % | 28 | 70,0 % |
| | Sí | 25 | 31,3 % | 13 | 32,5 % | 12 | 30,0 % |
| ANESTESIA COMPLEMENTARIA | Total | 80 | 100,0 % | 40 | 100,0 % | 40 | 100,0 % |
| | No | 32 | 40,0 % | 16 | 40,0 % | 16 | 40,0 % |
| | Sólo intraligamentosa | 23 | 28,7 % | 11 | 27,5 % | 12 | 30,0 % |
| | Sólo intrapulpar | 11 | 13,8 % | 7 | 17,5 % | 4 | 10,0 % |
| | Ambas | 14 | 17,5 % | 6 | 15,0 % | 8 | 20,0 % |

## T13.- RX CONDUCTOMETRIA según GRUPO

| | GRUPO | | | | | |
|---|---|---|---|---|---|---|
| | Total | | PEC | | POT | |
| | N | % | N | % | N | % |
| Total | 80 | 100,0 % | 40 | 100,0 % | 40 | 100,0 % |
| No | 80 | 100,0 % | 40 | 100,0 % | 40 | 100,0 % |

## T14.- SANGRADO PROFUSO según GRUPO

| | GRUPO | | | | | |
|---|---|---|---|---|---|---|
| | Total | | PEC | | POT | |
| | N | % | N | % | N | % |
| Total | 80 | 100,0 % | 40 | 100,0 % | 40 | 100,0 % |
| No | 51 | 63,7 % | 24 | 60,0 % | 27 | 67,5 % |
| Sí | 29 | 36,3 % | 16 | 40,0 % | 13 | 32,5 % |

## T15.- SANGRADO CONDUCTO según GRUPO

| | GRUPO | | | | | |
|---|---|---|---|---|---|---|
| | Total | | PEC | | POT | |
| | N | % | N | % | N | % |
| Total | 80 | 100,0 % | 40 | 100,0 % | 40 | 100,0 % |
| No | 59 | 73,8 % | 28 | 70,0 % | 31 | 77,5 % |
| Sí | 21 | 26,3 % | 12 | 30,0 % | 9 | 22,5 % |

## T15b.- CONDUCTO QUE SANGRA según GRUPO

| | GRUPO | | | | | |
|---|---|---|---|---|---|---|
| | Total | | PEC | | POT | |
| | N | % | N | % | N | % |
| Total | 21 | 100,0 % | 12 | 100,0 % | 9 | 100,0 % |
| V | 1 | 4,8 % | 0 | 0,0 % | 1 | 11,1 % |
| P | 9 | 42,9 % | 7 | 58,3 % | 2 | 22,2 % |
| D | 3 | 14,3 % | 2 | 16,7 % | 1 | 11,1 % |
| C | 7 | 33,3 % | 2 | 16,7 % | 5 | 55,6 % |
| MV/D | 1 | 4,8 % | 1 | 8,3 % | 0 | 0,0 % |

## T16.- CALCIFICACION según GRUPO

| | GRUPO | | | | | |
|---|---|---|---|---|---|---|
| | Total | | PEC | | POT | |
| | N | % | N | % | N | % |
| Total | 80 | 100,0 % | 40 | 100,0 % | 40 | 100,0 % |
| No | 73 | 91,3 % | 36 | 90,0 % | 37 | 92,5 % |
| Sí | 7 | 8,8 % | 4 | 10,0 % | 3 | 7,5 % |

## T17.- RECONSTRUCCIÓN PRE-ENDO según GRUPO

| | GRUPO | | |
|---|---|---|---|
| | Total | PEC | POT |

| | N | % | N | % | N | % |
|---|---|---|---|---|---|---|
| Total | 80 | 100,0 % | 40 | 100,0 % | 40 | 100,0 % |
| No | 49 | 61,3 % | 23 | 57,5 % | 26 | 65,0 % |
| Sí | 31 | 38,8 % | 17 | 42,5 % | 14 | 35,0 % |

T18.- GRADO DOLOR PRE según GRUPO

| | GRUPO | | |
|---|---|---|---|
| | Total | PEC | POT |
| N | 80 | 40 | 40 |
| Media | 5,8 | 6,0 | 5,6 |
| Desviación típica | 2,8 | 2,6 | 3,1 |
| Mínimo | 0,0 | 0,0 | 0,0 |
| Máximo | 10,0 | 10,0 | 10,0 |
| Percentil 25 | 4,0 | 4,0 | 4,0 |
| Mediana | 6,5 | 7,0 | 6,0 |
| Percentil 75 | 8,0 | 8,0 | 8,0 |

T19.- GRADO NERVIOSISMO PRE según GRUPO

| | GRUPO | | |
|---|---|---|---|
| | Total | PEC | POT |
| N | 80 | 40 | 40 |
| Media | 3,2 | 3,2 | 3,3 |
| Desviación típica | 3,4 | 3,2 | 3,5 |
| Mínimo | 0,0 | 0,0 | 0,0 |
| Máximo | 10,0 | 10,0 | 10,0 |
| Percentil 25 | 0,0 | 0,0 | 0,0 |
| Mediana | 2,0 | 2,0 | 2,5 |
| Percentil 75 | 6,0 | 5,5 | 6,0 |

T20.- GRADO MOLESTIAS AL MASTICAR PRE según GRUPO

| | GRUPO |
|---|---|

| | Total | PEC | POT |
|---|---|---|---|
| N | 80 | 40 | 40 |
| Media | 6,5 | 7,2 | 5,8 |
| Desviación típica | 3,3 | 2,7 | 3,6 |
| Mínimo | 0,0 | 0,0 | 0,0 |
| Máximo | 10,0 | 10,0 | 10,0 |
| Percentil 25 | 4,0 | 5,0 | 3,0 |
| Mediana | 7,0 | 8,0 | 6,5 |
| Percentil 75 | 9,0 | 9,0 | 9,5 |

T21- TIEMPOS DE INTERVENCIÓN (minutos) según GRUPO

| | | GRUPO | | |
|---|---|---|---|---|
| | | Total | PEC | POT |
| TIEMPO ANESTESIA A APERTURA | N | 79 | 39 | 40 |
| | Media | 16,6 | 19,2 | 14,2 |
| | Desviación típica | 10,6 | 11,6 | 9,0 |
| | Mínimo | 3,0 | 6,0 | 3,0 |
| | Máximo | 63,0 | 63,0 | 50,0 |
| | Percentil 25 | 10,0 | 12,0 | 8,5 |
| | Mediana | 14,0 | 15,0 | 11,5 |
| | Percentil 75 | 20,0 | 22,0 | 19,0 |
| TIEMPO APERTURA A FINAL | N | 79 | 39 | 40 |
| | Media | 10,3 | 13,4 | 7,4 |
| | Desviación típica | 5,6 | 6,1 | 3,0 |
| | Mínimo | 3,0 | 6,0 | 3,0 |
| | Máximo | 30,0 | 30,0 | 16,0 |
| | Percentil 25 | 6,0 | 8,0 | 5,0 |
| | Mediana | 8,0 | 12,0 | 7,0 |
| | Percentil 75 | 13,0 | 18,0 | 8,0 |

T22- EVALUACIÓN DURACIÓN TRATAMIENTO (0-muy corto a 10-demasiado largo) según GRUPO

| | | GRUPO | | |
|---|---|---|---|---|
| | | Total | PEC | POT |
| DURACION | N | 80 | 40 | 40 |
| | Media | 2,6 | 3,1 | 2,0 |
| | Desviación típica | 1,8 | 1,9 | 1,6 |
| | Mínimo | 0,0 | 0,0 | 0,0 |
| | Máximo | 10,0 | 10,0 | 5,0 |
| | Percentil 25 | 1,0 | 2,0 | 0,0 |
| | Mediana | 3,0 | 3,0 | 3,0 |
| | Percentil 75 | 3,0 | 3,5 | 3,0 |

T23- EVALUACIÓN INCOMODIDAD TRATAMIENTO (0-ninguna a 10-muy desagradable) según GRUPO

| | | GRUPO | | |
|---|---|---|---|---|
| | | Total | PEC | POT |
| INCOMODIDAD | N | 80 | 40 | 40 |
| | Media | 1,6 | 1,5 | 1,6 |
| | Desviación típica | 1,9 | 2,0 | 1,8 |
| | Mínimo | 0,0 | 0,0 | 0,0 |
| | Máximo | 7,0 | 7,0 | 6,0 |
| | Percentil 25 | 0,0 | 0,0 | 0,0 |
| | Mediana | 1,0 | 0,0 | 1,0 |
| | Percentil 75 | 3,0 | 3,0 | 3,0 |

T24.- DATOS POST-OPERATORIOS según GRUPO

| | | GRUPO | | | | | |
|---|---|---|---|---|---|---|---|
| | | Total | | PEC | | POT | |
| | | N | % | N | % | N | % |
| AINES | Total | 80 | 100,0 % | 40 | 100,0 % | 40 | 100,0 % |
| | No | 66 | 82,5 % | 36 | 90,0 % | 30 | 75,0 % |
| | Sí | 14 | 17,5 % | 4 | 10,0 % | 10 | 25,0 % |

| | | | | | | | |
|---|---|---|---|---|---|---|---|
| ANALGESICOS | Total | 80 | 100,0 % | 40 | 100,0 % | 40 | 100,0 % |
| | No | 64 | 80,0 % | 30 | 75,0 % | 34 | 85,0 % |
| | Sí | 16 | 20,0 % | 10 | 25,0 % | 6 | 15,0 % |
| DOLOR FUNCION | Total | 80 | 100,0 % | 40 | 100,0 % | 40 | 100,0 % |
| | No | 68 | 85,0 % | 36 | 90,0 % | 32 | 80,0 % |
| | Sí | 12 | 15,0 % | 4 | 10,0 % | 8 | 20,0 % |
| DOLOR PREAURICULAR | Total | 80 | 100,0 % | 40 | 100,0 % | 40 | 100,0 % |
| | No | 79 | 98,8 % | 39 | 97,5 % | 40 | 100,0 % |
| | Sí | 1 | 1,3 % | 1 | 2,5 % | 0 | 0,0 % |
| INFLAMACION ENCIA | Total | 80 | 100,0 % | 40 | 100,0 % | 40 | 100,0 % |
| | No | 76 | 95,0 % | 38 | 95,0 % | 38 | 95,0 % |
| | Sí | 4 | 5,0 % | 2 | 5,0 % | 2 | 5,0 % |

T24b.- MEDICACIÓN DOLOR POST según GRUPO

| | GRUPO | | | | | |
|---|---|---|---|---|---|---|
| | Total | | PEC | | POT | |
| | N | % | N | % | N | % |
| Total | 80 | 100,0 % | 40 | 100,0 % | 40 | 100,0 % |
| Ninguna | 56 | 70,0 % | 28 | 70,0 % | 28 | 70,0 % |
| Sólo AINES | 8 | 10,0 % | 2 | 5,0 % | 6 | 15,0 % |
| Sólo analgésicos | 10 | 12,5 % | 8 | 20,0 % | 2 | 5,0 % |
| Ambos | 6 | 7,5 % | 2 | 5,0 % | 4 | 10,0 % |

T25- EVOLUCIÓN DOLOR (VAS) según GRUPO

| | | GRUPO | | |
|---|---|---|---|---|
| | | Total | PEC | POT |
| DOLOR pre | N | 80 | 40 | 40 |
| | Media | 5,8 | 6,0 | 5,6 |
| | Desviación típica | 2,8 | 2,6 | 3,1 |
| | Mínimo | 0,0 | 0,0 | 0,0 |
| | Máximo | 10,0 | 10,0 | 10,0 |
| | Percentil 25 | 4,0 | 4,0 | 4,0 |

| | | Total | PEC | POT |
|---|---|---|---|---|
| | Mediana | 6,5 | 7,0 | 6,0 |
| | Percentil 75 | 8,0 | 8,0 | 8,0 |
| DOLOR 6h | N | 80 | 40 | 40 |
| | Media | 2,1 | 2,2 | 2,0 |
| | Desviación típica | 2,4 | 2,6 | 2,3 |
| | Mínimo | 0,0 | 0,0 | 0,0 |
| | Máximo | 9,0 | 9,0 | 9,0 |
| | Percentil 25 | 0,0 | 0,0 | 0,0 |
| | Mediana | 2,0 | 2,0 | 1,0 |
| | Percentil 75 | 3,0 | 3,5 | 3,0 |
| DOLOR 24h | N | 80 | 40 | 40 |
| | Media | 1,5 | 1,8 | 1,3 |
| | Desviación típica | 2,1 | 2,3 | 2,0 |
| | Mínimo | 0,0 | 0,0 | 0,0 |
| | Máximo | 9,0 | 9,0 | 7,0 |
| | Percentil 25 | 0,0 | 0,0 | 0,0 |
| | Mediana | 1,0 | 1,0 | 0,0 |
| | Percentil 75 | 2,0 | 2,5 | 1,5 |
| DOLOR 3d | N | 80 | 40 | 40 |
| | Media | 1,3 | 1,3 | 1,3 |
| | Desviación típica | 2,0 | 2,1 | 2,0 |
| | Mínimo | 0,0 | 0,0 | 0,0 |
| | Máximo | 10,0 | 10,0 | 10,0 |
| | Percentil 25 | 0,0 | 0,0 | 0,0 |
| | Mediana | 0,0 | 0,0 | 0,5 |
| | Percentil 75 | 2,0 | 2,0 | 2,0 |

T25b- REDUCCIÓN DOLOR (VAS) 3d - PRE según GRUPO

| | | GRUPO | | |
|---|---|---|---|---|
| | | Total | PEC | POT |
| | N | 80 | 40 | 40 |
| | Media | -4,5 | -4,7 | -4,3 |

| | | | | |
|---|---|---|---|---|
| DIF.DOLOR.3d_PRE | Desviación típica | 3,5 | 3,2 | 3,8 |
| | Mínimo | -9,0 | -9,0 | -9,0 |
| | Máximo | 6,0 | 4,0 | 6,0 |
| | Percentil 25 | -7,0 | -7,0 | -7,0 |
| | Mediana | -6,0 | -6,0 | -5,0 |
| | Percentil 75 | -1,5 | -1,5 | -1,5 |

T25c.- TIEMPO HASTA NO DOLOR / DOLOR LEVE según GRUPO

| | | GRUPO | | | | | |
|---|---|---|---|---|---|---|---|
| | | Total | | PEC | | POT | |
| | | N | % | N | % | N | % |
| TIEMPO HASTA NO DOLOR DEFINITIVO | Total | 80 | 100,0 % | 40 | 100,0 % | 40 | 100,0 % |
| | Pre | 2 | 2,5 % | 1 | 2,5 % | 1 | 2,5 % |
| | 6h | 20 | 25,0 % | 10 | 25,0 % | 10 | 25,0 % |
| | 24h | 7 | 8,8 % | 2 | 5,0 % | 5 | 12,5 % |
| | 3d | 12 | 15,0 % | 9 | 22,5 % | 3 | 7,5 % |
| | No se alcanza | 39 | 48,8 % | 18 | 45,0 % | 21 | 52,5 % |
| TIEMPO HASTA DOLOR LEVE DEFINITIVO | Total | 80 | 100,0 % | 40 | 100,0 % | 40 | 100,0 % |
| | Pre | 4 | 5,0 % | 2 | 5,0 % | 2 | 5,0 % |
| | 6h | 53 | 66,3 % | 24 | 60,0 % | 29 | 72,5 % |
| | 24h | 7 | 8,8 % | 5 | 12,5 % | 2 | 5,0 % |
| | 3d | 7 | 8,8 % | 4 | 10,0 % | 3 | 7,5 % |
| | No se alcanza | 9 | 11,3 % | 5 | 12,5 % | 4 | 10,0 % |

T26- EVALUACIÓN SATISFACCIÓN GENERAL TRATAMIENTO (0-nada satisfecho a 10-muy satisfecho) según GRUPO

| | | GRUPO | | |
|---|---|---|---|---|
| | | Total | PEC | POT |
| SATISFACCION | N | 80 | 40 | 40 |
| | Media | 9,1 | 9,2 | 9,1 |
| | Desviación típica | 1,8 | 1,7 | 2,0 |
| | Mínimo | 1,0 | 1,0 | 1,0 |

| | | | | | | | | | |
|---|---|---|---|---|---|---|---|---|---|
| [illegible] | Máximo | 10,0 | 10,0 | 10,0 | | | | | |
| | Percentil 25 | 9,0 | 9,0 | 9,0 | | | | | |
| | Mediana | 10,0 | 10,0 | 10,0 | | | | | |
| | Percentil 75 | 10,0 | 10,0 | 10,0 | | | | | |

T27- EVOLUCIÓN DOLOR (VAS) según GRUPO y AINES PRE

| | | GRUPO | | | | | | | |
|---|---|---|---|---|---|---|---|---|---|
| | | Total | | | PEC | | | POT | |
| | | AINES PRE | | | AINES PRE | | | AINES PRE | |
| | | Total | No | Sí | Total | No | Sí | Total | No |
| DOLOR pre | N | 80 | 43 | 37 | 40 | 23 | 17 | 40 | 20 |
| | Media | 5,8 | 5,4 | 6,2 | 6,0 | 5,2 | 7,0 | 5,6 | 5,8 |
| | Desviación típica | 2,8 | 2,8 | 2,8 | 2,6 | 2,8 | 1,8 | 3,1 | 2,8 |
| | Mínimo | 0,0 | 0,0 | 0,0 | 0,0 | 0,0 | 3,0 | 0,0 | 0,0 |
| | Máximo | 10,0 | 10,0 | 10,0 | 10,0 | 9,0 | 10,0 | 10,0 | 10,0 |
| | Percentil 25 | 4,0 | 4,0 | 4,0 | 4,0 | 3,0 | 7,0 | 4,0 | 4,5 |
| | Mediana | 6,5 | 6,0 | 7,0 | 7,0 | 6,0 | 7,0 | 6,0 | 6,0 |
| | Percentil 75 | 8,0 | 7,0 | 8,0 | 8,0 | 7,0 | 8,0 | 8,0 | 7,5 |
| DOLOR 6h | N | 80 | 43 | 37 | 40 | 23 | 17 | 40 | 20 |
| | Media | 2,1 | 1,9 | 2,4 | 2,2 | 1,8 | 2,8 | 2,0 | 2,1 |
| | Desviación típica | 2,4 | 2,4 | 2,5 | 2,6 | 2,4 | 2,8 | 2,3 | 2,5 |
| | Mínimo | 0,0 | 0,0 | 0,0 | 0,0 | 0,0 | 0,0 | 0,0 | 0,0 |
| | Máximo | 9,0 | 9,0 | 9,0 | 9,0 | 9,0 | 8,0 | 9,0 | 7,0 |
| | Percentil 25 | 0,0 | 0,0 | 0,0 | 0,0 | 0,0 | 0,0 | 0,0 | 0,0 |
| | Mediana | 2,0 | 1,0 | 2,0 | 2,0 | 1,0 | 3,0 | 1,0 | 1,0 |
| | Percentil 75 | 3,0 | 3,0 | 3,0 | 3,5 | 3,0 | 4,0 | 3,0 | 3,0 |
| DOLOR 24h | N | 80 | 43 | 37 | 40 | 23 | 17 | 40 | 20 |
| | Media | 1,5 | 1,5 | 1,5 | 1,8 | 1,7 | 1,8 | 1,3 | 1,3 |
| | Desviación típica | 2,1 | 2,1 | 2,1 | 2,3 | 2,3 | 2,2 | 2,0 | 1,9 |
| | Mínimo | 0,0 | 0,0 | 0,0 | 0,0 | 0,0 | 0,0 | 0,0 | 0,0 |
| | Máximo | 9,0 | 9,0 | 8,0 | 9,0 | 9,0 | 8,0 | 7,0 | 5,0 |
| | Percentil 25 | 0,0 | 0,0 | 0,0 | 0,0 | 0,0 | 0,0 | 0,0 | 0,0 |

| | | | | | | | | | |
|---|---|---|---|---|---|---|---|---|---|
| | Mediana | 1,0 | 1,0 | 1,0 | 1,0 | 1,0 | 1,0 | 0,0 | 0,0 |
| | Percentil 75 | 2,0 | 2,0 | 2,0 | 2,5 | 2,0 | 3,0 | 1,5 | 2,0 |
| DOLOR 3d | N | 80 | 43 | 37 | 40 | 23 | 17 | 40 | 20 |
| | Media | 1,3 | 1,2 | 1,4 | 1,3 | 1,0 | 1,5 | 1,3 | 1,4 |
| | Desviación típica | 2,0 | 1,9 | 2,2 | 2,1 | 2,3 | 1,8 | 2,0 | 1,5 |
| | Mínimo | 0,0 | 0,0 | 0,0 | 0,0 | 0,0 | 0,0 | 0,0 | 0,0 |
| | Máximo | 10,0 | 10,0 | 10,0 | 10,0 | 10,0 | 6,0 | 10,0 | 5,0 |
| | Percentil 25 | 0,0 | 0,0 | 0,0 | 0,0 | 0,0 | 0,0 | 0,0 | 0,0 |
| | Mediana | 0,0 | 0,0 | 0,0 | 0,0 | 0,0 | 1,0 | 0,5 | 1,0 |
| | Percentil 75 | 2,0 | 2,0 | 2,0 | 2,0 | 2,0 | 2,0 | 2,0 | 2,5 |

## T27b- REDUCCIÓN DOLOR (VAS) 3d - PRE según GRUPO y AINES PRE

| | | GRUPO | | | | | | | |
|---|---|---|---|---|---|---|---|---|---|
| | | Total | | | PEC | | | POT | |
| | | AINES PRE | | | AINES PRE | | | AINES PRE | |
| | | Total | No | Sí | Total | No | Sí | Total | No |
| DIF.DOLOR.3d_PRE | N | 80 | 43 | 37 | 40 | 23 | 17 | 40 | 20 |
| | Media | -4,5 | -4,3 | -4,8 | -4,7 | -4,1 | -5,5 | -4,3 | -4,4 |
| | Desviación típica | 3,5 | 3,2 | 3,8 | 3,2 | 3,5 | 2,5 | 3,8 | 3,0 |
| | Mínimo | -9,0 | -9,0 | -9,0 | -9,0 | -9,0 | -9,0 | -9,0 | -8,0 |
| | Máximo | 6,0 | 4,0 | 6,0 | 4,0 | 4,0 | -1,0 | 6,0 | 3,0 |
| | Percentil 25 | -7,0 | -7,0 | -8,0 | -7,0 | -7,0 | -8,0 | -7,0 | -6,5 |
| | Mediana | -6,0 | -5,0 | -6,0 | -6,0 | -6,0 | -6,0 | -5,0 | -5,0 |
| | Percentil 75 | -1,5 | -1,0 | -2,0 | -1,5 | -1,0 | -4,0 | -1,5 | -2,5 |

## T27c.- TIEMPO HASTA NO DOLOR / DOLOR LEVE según GRUPO y AINES PRE

| | | | | | | | |
|---|---|---|---|---|---|---|---|
| Total | | | | | | | |
| AINES PRE | | | | | | | |
| Total | | No | | Sí | | Total | |
| N | % | N | % | N | % | N | % |

| | | | | | | | | | |
|---|---|---|---|---|---|---|---|---|---|
| TIEMPO HASTA NO DOLOR DEFINITIVO | Total | 80 | 100,0 % | 43 | 100,0 % | 37 | 100,0 % | 40 | 100,0 % |
| | Pre | 2 | 2,5 % | 1 | 2,3 % | 1 | 2,7 % | 1 | 2,5 % |
| | 6h | 20 | 25,0 % | 12 | 27,9 % | 8 | 21,6 % | 10 | 25,0 % |
| | 24h | 7 | 8,8 % | 3 | 7,0 % | 4 | 10,8 % | 2 | 5,0 % |
| | 3d | 12 | 15,0 % | 6 | 14,0 % | 6 | 16,2 % | 9 | 22,5 % |
| | No se alcanza | 39 | 48,8 % | 21 | 48,8 % | 18 | 48,6 % | 18 | 45,0 % |
| TIEMPO HASTA DOLOR LEVE DEFINITIVO | Total | 80 | 100,0 % | 43 | 100,0 % | 37 | 100,0 % | 40 | 100,0 % |
| | Pre | 4 | 5,0 % | 2 | 4,7 % | 2 | 5,4 % | 2 | 5,0 % |
| | 6h | 53 | 66,3 % | 30 | 69,8 % | 23 | 62,2 % | 24 | 60,0 % |
| | 24h | 7 | 8,8 % | 3 | 7,0 % | 4 | 10,8 % | 5 | 12,5 % |
| | 3d | 7 | 8,8 % | 5 | 11,6 % | 2 | 5,4 % | 4 | 10,0 % |
| | No se alcanza | 9 | 11,3 % | 3 | 7,0 % | 6 | 16,2 % | 5 | 12,5 % |

T28- EVALUACIÓN SATISFACCIÓN GENERAL TRATAMIENTO (0-nada satisfecho a 10-muy satisfecho) según GRUPO y AINES PRE

| | | GRUPO | | | | | | | |
|---|---|---|---|---|---|---|---|---|---|
| | | Total | | | PEC | | | POT | |
| | | AINES PRE | | | AINES PRE | | | AINES PRE | |
| | | Total | No | Sí | Total | No | Sí | Total | No |
| SATISFACCION | N | 80 | 43 | 37 | 40 | 23 | 17 | 40 | 20 |
| | Media | 9,1 | 9,2 | 9,0 | 9,2 | 9,1 | 9,4 | 9,1 | 9,4 |
| | Desviación típica | 1,8 | 1,7 | 2,0 | 1,7 | 2,0 | 1,3 | 2,0 | 1,3 |
| | Mínimo | 1,0 | 1,0 | 1,0 | 1,0 | 1,0 | 5,0 | 1,0 | 6,0 |
| | Máximo | 10,0 | 10,0 | 10,0 | 10,0 | 10,0 | 10,0 | 10,0 | 10,0 |
| | Percentil 25 | 9,0 | 9,0 | 9,0 | 9,0 | 8,0 | 9,0 | 9,0 | 10,0 |
| | Mediana | 10,0 | 10,0 | 10,0 | 10,0 | 10,0 | 10,0 | 10,0 | 10,0 |
| | Percentil 75 | 10,0 | 10,0 | 10,0 | 10,0 | 10,0 | 10,0 | 10,0 | 10,0 |

T29- EVOLUCIÓN DOLOR (VAS) según GRUPO y PAA

| | GRUPO | | |
|---|---|---|---|
| | Total | PEC | POT |
| | PAA | PAA | PAA |

| | | Total | No | Sí | Total | No | Sí | Total | No |
|---|---|---|---|---|---|---|---|---|---|
| DOLOR pre | N | 80 | 47 | 33 | 40 | 20 | 20 | 40 | 27 |
| | Media | 5,8 | 5,2 | 6,6 | 6,0 | 5,1 | 6,8 | 5,6 | 5,3 |
| | Desviación típica | 2,8 | 2,8 | 2,8 | 2,6 | 2,9 | 2,0 | 3,1 | 2,7 |
| | Mínimo | 0,0 | 0,0 | 0,0 | 0,0 | 0,0 | 3,0 | 0,0 | 0,0 |
| | Máximo | 10,0 | 10,0 | 10,0 | 10,0 | 9,0 | 10,0 | 10,0 | 10,0 |
| | Percentil 25 | 4,0 | 3,0 | 6,0 | 4,0 | 2,5 | 6,0 | 4,0 | 4,0 |
| | Mediana | 6,5 | 6,0 | 7,0 | 7,0 | 6,0 | 7,0 | 6,0 | 6,0 |
| | Percentil 75 | 8,0 | 7,0 | 8,0 | 8,0 | 7,5 | 8,0 | 8,0 | 7,0 |
| DOLOR 6h | N | 80 | 47 | 33 | 40 | 20 | 20 | 40 | 27 |
| | Media | 2,1 | 2,0 | 2,3 | 2,2 | 1,7 | 2,8 | 2,0 | 2,2 |
| | Desviación típica | 2,4 | 2,5 | 2,4 | 2,6 | 2,5 | 2,6 | 2,3 | 2,5 |
| | Mínimo | 0,0 | 0,0 | 0,0 | 0,0 | 0,0 | 0,0 | 0,0 | 0,0 |
| | Máximo | 9,0 | 9,0 | 8,0 | 9,0 | 9,0 | 8,0 | 9,0 | 9,0 |
| | Percentil 25 | 0,0 | 0,0 | 0,0 | 0,0 | 0,0 | 0,0 | 0,0 | 0,0 |
| | Mediana | 2,0 | 1,0 | 2,0 | 2,0 | 0,0 | 2,5 | 1,0 | 1,0 |
| | Percentil 75 | 3,0 | 3,0 | 4,0 | 3,5 | 2,5 | 4,0 | 3,0 | 3,0 |
| DOLOR 24h | N | 80 | 47 | 33 | 40 | 20 | 20 | 40 | 27 |
| | Media | 1,5 | 1,5 | 1,5 | 1,8 | 1,8 | 1,8 | 1,3 | 1,3 |
| | Desviación típica | 2,1 | 2,3 | 1,9 | 2,3 | 2,5 | 2,1 | 2,0 | 2,1 |
| | Mínimo | 0,0 | 0,0 | 0,0 | 0,0 | 0,0 | 0,0 | 0,0 | 0,0 |
| | Máximo | 9,0 | 9,0 | 8,0 | 9,0 | 9,0 | 8,0 | 7,0 | 7,0 |
| | Percentil 25 | 0,0 | 0,0 | 0,0 | 0,0 | 0,0 | 0,0 | 0,0 | 0,0 |
| | Mediana | 1,0 | 0,0 | 1,0 | 1,0 | 1,0 | 1,0 | 0,0 | 0,0 |
| | Percentil 75 | 2,0 | 2,0 | 2,0 | 2,5 | 2,0 | 3,0 | 1,5 | 2,0 |
| DOLOR 3d | N | 80 | 47 | 33 | 40 | 20 | 20 | 40 | 27 |
| | Media | 1,3 | 1,4 | 1,2 | 1,3 | 1,2 | 1,3 | 1,3 | 1,5 |
| | Desviación típica | 2,0 | 2,3 | 1,6 | 2,1 | 2,4 | 1,8 | 2,0 | 2,2 |
| | Mínimo | 0,0 | 0,0 | 0,0 | 0,0 | 0,0 | 0,0 | 0,0 | 0,0 |
| | Máximo | 10,0 | 10,0 | 6,0 | 10,0 | 10,0 | 6,0 | 10,0 | 10,0 |
| | Percentil 25 | 0,0 | 0,0 | 0,0 | 0,0 | 0,0 | 0,0 | 0,0 | 0,0 |
| | Mediana | 0,0 | 0,0 | 0,0 | 0,0 | 0,0 | 0,5 | 0,5 | 1,0 |

| | | | | | | | | | |
|---|---|---|---|---|---|---|---|---|---|
| | Percentil 75 | 2,0 | 2,0 | 2,0 | 2,0 | 2,0 | 2,0 | 2,0 | 3,0 |

## T29b- REDUCCIÓN DOLOR (VAS) 3d - PRE según GRUPO y PAA

| | | GRUPO | | | | | | | |
|---|---|---|---|---|---|---|---|---|---|
| | | Total | | | PEC | | | POT | |
| | | PAA | | | PAA | | | PAA | |
| | | Total | No | Sí | Total | No | Sí | Total | No |
| DIF.DOLOR.3d_PRE | N | 80 | 47 | 33 | 40 | 20 | 20 | 40 | 27 |
| | Media | -4,5 | -3,9 | -5,4 | -4,7 | -3,9 | -5,5 | -4,3 | -3,9 |
| | Desviación típica | 3,5 | 3,6 | 3,2 | 3,2 | 3,6 | 2,5 | 3,8 | 3,6 |
| | Mínimo | -9,0 | -8,0 | -9,0 | -9,0 | -8,0 | -9,0 | -9,0 | -8,0 |
| | Máximo | 6,0 | 6,0 | 4,0 | 4,0 | 4,0 | -1,0 | 6,0 | 6,0 |
| | Percentil 25 | -7,0 | -6,0 | -8,0 | -7,0 | -6,5 | -8,0 | -7,0 | -6,0 |
| | Mediana | -6,0 | -5,0 | -6,0 | -6,0 | -6,0 | -6,0 | -5,0 | -5,0 |
| | Percentil 75 | -1,5 | -1,0 | -3,0 | -1,5 | -1,0 | -3,5 | -1,5 | -1,0 |

## T29c.- TIEMPO HASTA NO DOLOR / DOLOR LEVE según GRUPO y PAA

| | | | | | | | | | |
|---|---|---|---|---|---|---|---|---|---|
| | | Total | | | | | | | |
| | | PAA | | | | | | | |
| | | Total | | No | | Sí | | Total | |
| | | N | % | N | % | N | % | N | % |
| TIEMPO HASTA NO DOLOR DEFINITIVO | Total | 80 | 100,0 % | 47 | 100,0 % | 33 | 100,0 % | 40 | 100,0 % |
| | Pre | 2 | 2,5 % | 1 | 2,1 % | 1 | 3,0 % | 1 | 2,5 % |
| | 6h | 20 | 25,0 % | 14 | 29,8 % | 6 | 18,2 % | 10 | 25,0 % |
| | 24h | 7 | 8,8 % | 4 | 8,5 % | 3 | 9,1 % | 2 | 5,0 % |
| | 3d | 12 | 15,0 % | 5 | 10,6 % | 7 | 21,2 % | 9 | 22,5 % |
| | No se alcanza | 39 | 48,8 % | 23 | 48,9 % | 16 | 48,5 % | 18 | 45,0 % |
| TIEMPO HASTA DOLOR LEVE | Total | 80 | 100,0 % | 47 | 100,0 % | 33 | 100,0 % | 40 | 100,0 % |
| | Pre | 4 | 5,0 % | 2 | 4,3 % | 2 | 6,1 % | 2 | 5,0 % |
| | 6h | 53 | 66,3 % | 33 | 70,2 % | 20 | 60,6 % | 24 | 60,0 % |

| | | | | | | | | | |
|---|---|---|---|---|---|---|---|---|---|
| [illegible] DEFINITIVO | 24h | 7 | 8,8 % | 3 | 6,4 % | 4 | 12,1 % | 5 | 12,5 % |
| | 3d | 7 | 8,8 % | 4 | 8,5 % | 3 | 9,1 % | 4 | 10,0 % |
| | No se alcanza | 9 | 11,3 % | 5 | 10,6 % | 4 | 12,1 % | 5 | 12,5 % |

T30- EVALUACIÓN SATISFACCIÓN GENERAL TRATAMIENTO (0-nada satisfecho a 10-muy satisfecho) según GRUPO y PAA

| | | GRUPO | | | | | | | |
|---|---|---|---|---|---|---|---|---|---|
| | | Total | | | PEC | | | POT | |
| | | PAA | | | PAA | | | PAA | |
| | | Total | No | Sí | Total | No | Sí | Total | No |
| SATISFACCION | N | 80 | 47 | 33 | 40 | 20 | 20 | 40 | 27 |
| | Media | 9,1 | 9,1 | 9,2 | 9,2 | 9,2 | 9,3 | 9,1 | 9,1 |
| | Desviación típica | 1,8 | 2,1 | 1,3 | 1,7 | 2,1 | 1,3 | 2,0 | 2,3 |
| | Mínimo | 1,0 | 1,0 | 5,0 | 1,0 | 1,0 | 5,0 | 1,0 | 1,0 |
| | Máximo | 10,0 | 10,0 | 10,0 | 10,0 | 10,0 | 10,0 | 10,0 | 10,0 |
| | Percentil 25 | 9,0 | 9,0 | 9,0 | 9,0 | 9,0 | 9,0 | 9,0 | 10,0 |
| | Mediana | 10,0 | 10,0 | 10,0 | 10,0 | 10,0 | 10,0 | 10,0 | 10,0 |
| | Percentil 75 | 10,0 | 10,0 | 10,0 | 10,0 | 10,0 | 10,0 | 10,0 | 10,0 |

T31.- DOLOR AL MASTICAR POST-OPERATORIO según GRUPO y PAA

| | GRU | | | | | | | | |
|---|---|---|---|---|---|---|---|---|---|
| | Total | | | | | | PE | | |
| | PAA | | | | | | PA | | |
| | Total | | No | | Sí | | Total | | N |
| | N | % | N | % | N | % | N | % | N |
| Total | 80 | 100,0 % | 47 | 100,0 % | 33 | 100,0 % | 40 | 100,0 % | 20 |
| No | 68 | 85,0 % | 40 | 85,1 % | 28 | 84,8 % | 36 | 90,0 % | 19 |
| Sí | 12 | 15,0 % | 7 | 14,9 % | 5 | 15,2 % | 4 | 10,0 % | 1 |

T31b.- GRADO MOLESTIAS AL MASTICAR PRE según PAA y toma AINES PRE

| | AINES PRE | | |
|---|---|---|---|
| | Total | No | Sí |

| | PAA | | | PAA | | | PAA | | |
|---|---|---|---|---|---|---|---|---|---|
| | Total | No | Sí | Total | No | Sí | Total | No | Sí |
| N | 80 | 47 | 33 | 43 | 33 | 10 | 37 | 14 | 23 |
| Media | 6,5 | 5,3 | 8,2 | 6,0 | 5,5 | 7,7 | 7,1 | 4,7 | 8,5 |
| Desviación típica | 3,3 | 3,4 | 2,0 | 3,3 | 3,3 | 2,8 | 3,2 | 3,6 | 1,7 |
| Mínimo | 0,0 | 0,0 | 2,0 | 0,0 | 0,0 | 2,0 | 0,0 | 0,0 | 4,0 |
| Máximo | 10,0 | 10,0 | 10,0 | 10,0 | 10,0 | 10,0 | 10,0 | 10,0 | 10,0 |
| Percentil 25 | 4,0 | 3,0 | 7,0 | 4,0 | 3,0 | 7,0 | 5,0 | 1,0 | 7,0 |
| Mediana | 7,0 | 5,0 | 9,0 | 6,0 | 5,0 | 9,0 | 8,0 | 4,5 | 9,0 |
| Percentil 75 | 9,0 | 8,0 | 10,0 | 9,0 | 9,0 | 10,0 | 10,0 | 8,0 | 10,0 |

## T31c.- MEDICACIÓN DOLOR POST según GRUPO y PAA

| | GRU | | | | | | | | |
|---|---|---|---|---|---|---|---|---|---|
| | Total | | | | | | PE | | |
| | PAA | | | | | | PA | | |
| | Total | | No | | Sí | | Total | | N |
| | N | % | N | % | N | % | N | % | N |
| Total | 80 | 100,0 % | 47 | 100,0 % | 33 | 100,0 % | 40 | 100,0 % | 20 |
| Ninguna | 56 | 70,0 % | 36 | 76,6 % | 20 | 60,6 % | 28 | 70,0 % | 17 |
| Sólo AINES | 8 | 10,0 % | 4 | 8,5 % | 4 | 12,1 % | 2 | 5,0 % | 1 |
| Sólo analgésicos | 10 | 12,5 % | 4 | 8,5 % | 6 | 18,2 % | 8 | 20,0 % | 2 |
| Ambos | 6 | 7,5 % | 3 | 6,4 % | 3 | 9,1 % | 2 | 5,0 % | 0 |

## T32- EVOLUCIÓN DOLOR (VAS) según GRUPO y MOLESTIA MASTICAR pre

| | GRUPO | | | | | | | |
|---|---|---|---|---|---|---|---|---|
| | Total | | | PEC | | | POT | |
| | MOLESTIA MASTICAR PRE | | | MOLESTIA MASTICAR PRE | | | MOLESTIA MASTICAR PR | |
| | Total | Hasta moderada | Intensa | Total | Hasta moderada | Intensa | Total | Hasta moderada |
| N | 80 | 34 | 46 | 40 | 14 | 26 | 40 | 20 |
| Media | 5,8 | 4,6 | 6,7 | 6,0 | 4,6 | 6,7 | 5,6 | 4,5 |
| Desviación típica | 2,8 | 2,7 | 2,6 | 2,6 | 2,8 | 2,2 | 3,1 | 2,8 |

| | | | | | | | | | |
|---|---|---|---|---|---|---|---|---|---|
| DOLOR pre | Mínimo | 0,0 | 0,0 | 0,0 | 0,0 | 0,0 | 0,0 | 0,0 | 0,0 |
| | Máximo | 10,0 | 10,0 | 10,0 | 10,0 | 10,0 | 9,0 | 10,0 | 8,0 |
| | Percentil 25 | 4,0 | 2,0 | 6,0 | 4,0 | 3,0 | 6,0 | 4,0 | 2,0 |
| | Mediana | 6,5 | 5,0 | 7,0 | 7,0 | 5,0 | 7,0 | 6,0 | 5,0 |
| | Percentil 75 | 8,0 | 6,0 | 8,0 | 8,0 | 6,0 | 8,0 | 8,0 | 6,5 |
| DOLOR 6h | N | 80 | 34 | 46 | 40 | 14 | 26 | 40 | 20 |
| | Media | 2,1 | 2,0 | 2,2 | 2,2 | 1,7 | 2,5 | 2,0 | 2,2 |
| | Desviación típica | 2,4 | 2,7 | 2,2 | 2,6 | 2,6 | 2,6 | 2,3 | 2,8 |
| | Mínimo | 0,0 | 0,0 | 0,0 | 0,0 | 0,0 | 0,0 | 0,0 | 0,0 |
| | Máximo | 9,0 | 9,0 | 8,0 | 9,0 | 9,0 | 8,0 | 9,0 | 9,0 |
| | Percentil 25 | 0,0 | 0,0 | 0,0 | 0,0 | 0,0 | 0,0 | 0,0 | 0,0 |
| | Mediana | 2,0 | 1,0 | 2,0 | 2,0 | 0,0 | 2,0 | 1,0 | 1,0 |
| | Percentil 75 | 3,0 | 3,0 | 3,0 | 3,5 | 3,0 | 4,0 | 3,0 | 3,5 |
| DOLOR 24h | N | 80 | 34 | 46 | 40 | 14 | 26 | 40 | 20 |
| | Media | 1,5 | 1,6 | 1,4 | 1,8 | 2,1 | 1,6 | 1,3 | 1,3 |
| | Desviación típica | 2,1 | 2,5 | 1,8 | 2,3 | 2,8 | 1,9 | 2,0 | 2,3 |
| | Mínimo | 0,0 | 0,0 | 0,0 | 0,0 | 0,0 | 0,0 | 0,0 | 0,0 |
| | Máximo | 9,0 | 9,0 | 8,0 | 9,0 | 9,0 | 8,0 | 7,0 | 7,0 |
| | Percentil 25 | 0,0 | 0,0 | 0,0 | 0,0 | 0,0 | 0,0 | 0,0 | 0,0 |
| | Mediana | 1,0 | 0,0 | 1,0 | 1,0 | 1,0 | 1,0 | 0,0 | 0,0 |
| | Percentil 75 | 2,0 | 2,0 | 2,0 | 2,5 | 3,0 | 2,0 | 1,5 | 1,5 |
| DOLOR 3d | N | 80 | 34 | 46 | 40 | 14 | 26 | 40 | 20 |
| | Media | 1,3 | 1,1 | 1,4 | 1,3 | 0,8 | 1,5 | 1,3 | 1,3 |
| | Desviación típica | 2,0 | 2,0 | 2,1 | 2,1 | 1,3 | 2,4 | 2,0 | 2,4 |
| | Mínimo | 0,0 | 0,0 | 0,0 | 0,0 | 0,0 | 0,0 | 0,0 | 0,0 |
| | Máximo | 10,0 | 10,0 | 10,0 | 10,0 | 4,0 | 10,0 | 10,0 | 10,0 |
| | Percentil 25 | 0,0 | 0,0 | 0,0 | 0,0 | 0,0 | 0,0 | 0,0 | 0,0 |
| | Mediana | 0,0 | 0,0 | 0,5 | 0,0 | 0,0 | 0,0 | 0,5 | 0,0 |
| | Percentil 75 | 2,0 | 1,0 | 2,0 | 2,0 | 1,0 | 2,0 | 2,0 | 1,5 |

T32b- REDUCCIÓN DOLOR (VAS) 3d - PRE según GRUPO y MOLESTIA MASTICAR pre

| | GRUPO |
|---|---|

| | | Total | | | PEC | | | POT | |
|---|---|---|---|---|---|---|---|---|---|
| | | MOLESTIA MASTICAR PRE | | | MOLESTIA MASTICAR PRE | | | MOLESTIA MASTICAR PR | |
| | | Total | Hasta moderada | Intensa | Total | Hasta moderada | Intensa | Total | Hasta moderada |
| DIF.DOLOR.3d_PRE | N | 80 | 34 | 46 | 40 | 14 | 26 | 40 | 20 |
| | Media | -4,5 | -3,5 | -5,3 | -4,7 | -3,9 | -5,2 | -4,3 | -3,2 |
| | Desviación típica | 3,5 | 3,7 | 3,1 | 3,2 | 3,1 | 3,2 | 3,8 | 4,1 |
| | Mínimo | -9,0 | -9,0 | -9,0 | -9,0 | -9,0 | -9,0 | -9,0 | -8,0 |
| | Máximo | 6,0 | 6,0 | 4,0 | 4,0 | 1,0 | 4,0 | 6,0 | 6,0 |
| | Percentil 25 | -7,0 | -6,0 | -8,0 | -7,0 | -6,0 | -8,0 | -7,0 | -6,0 |
| | Mediana | -6,0 | -5,0 | -6,0 | -6,0 | -4,5 | -6,0 | -5,0 | -5,0 |
| | Percentil 75 | -1,5 | -1,0 | -4,0 | -1,5 | -1,0 | -4,0 | -1,5 | -0,5 |

T32c.- TIEMPO HASTA NO DOLOR / DOLOR LEVE según GRUPO y MOLESTIA MASTICAR pre

| | | Total | | | | | | | |
|---|---|---|---|---|---|---|---|---|---|
| | | MOLESTIA MASTICAR PRE | | | | | | | |
| | | Total | | Hasta moderada | | Intensa | | Total | |
| | | N | % | N | % | N | % | N | % |
| TIEMPO HASTA NO DOLOR DEFINITIVO | Total | 80 | 100,0 % | 34 | 100,0 % | 46 | 100,0 % | 40 | 100,0 % |
| | Pre | 2 | 2,5 % | 0 | 0,0 % | 2 | 4,3 % | 1 | 2,5 % |
| | 6h | 20 | 25,0 % | 11 | 32,4 % | 9 | 19,6 % | 10 | 25,0 % |
| | 24h | 7 | 8,8 % | 3 | 8,8 % | 4 | 8,7 % | 2 | 5,0 % |
| | 3d | 12 | 15,0 % | 5 | 14,7 % | 7 | 15,2 % | 9 | 22,5 % |
| | No se alcanza | 39 | 48,8 % | 15 | 44,1 % | 24 | 52,2 % | 18 | 45,0 % |
| TIEMPO HASTA DOLOR LEVE DEFINITIVO | Total | 80 | 100,0 % | 34 | 100,0 % | 46 | 100,0 % | 40 | 100,0 % |
| | Pre | 4 | 5,0 % | 0 | 0,0 % | 4 | 8,7 % | 2 | 5,0 % |
| | 6h | 53 | 66,3 % | 24 | 70,6 % | 29 | 63,0 % | 24 | 60,0 % |
| | 24h | 7 | 8,8 % | 3 | 8,8 % | 4 | 8,7 % | 5 | 12,5 % |
| | 3d | 7 | 8,8 % | 4 | 11,8 % | 3 | 6,5 % | 4 | 10,0 % |
| | No se alcanza | 9 | 11,3 % | 3 | 8,8 % | 6 | 13,0 % | 5 | 12,5 % |

## T33- EVALUACIÓN SATISFACCIÓN GENERAL TRATAMIENTO (0-nada satisfecho a 10-muy satisfecho) según GRUPO y MOLESTIA MASTICAR pre

| | | GRUPO | | | | | | | |
|---|---|---|---|---|---|---|---|---|---|
| | | Total | | | PEC | | | POT | |
| | | MOLESTIA MASTICAR PRE | | | MOLESTIA MASTICAR PRE | | | MOLESTIA MASTICAR PI | |
| | | Total | Hasta moderada | Intensa | Total | Hasta moderada | Intensa | Total | Hasta moderada |
| SATISFACCION | N | 80 | 34 | 46 | 40 | 14 | 26 | 40 | 20 |
| | Media | 9,1 | 9,6 | 8,7 | 9,2 | 9,6 | 9,0 | 9,1 | 9,7 |
| | Desviación típica | 1,8 | 0,8 | 2,2 | 1,7 | 0,7 | 2,0 | 2,0 | 0,9 |
| | Mínimo | 1,0 | 7,0 | 1,0 | 1,0 | 8,0 | 1,0 | 1,0 | 7,0 |
| | Máximo | 10,0 | 10,0 | 10,0 | 10,0 | 10,0 | 10,0 | 10,0 | 10,0 |
| | Percentil 25 | 9,0 | 10,0 | 8,0 | 9,0 | 10,0 | 9,0 | 9,0 | 10,0 |
| | Mediana | 10,0 | 10,0 | 10,0 | 10,0 | 10,0 | 10,0 | 10,0 | 10,0 |
| | Percentil 75 | 10,0 | 10,0 | 10,0 | 10,0 | 10,0 | 10,0 | 10,0 | 10,0 |

## T34.- DOLOR AL MASTICAR POST-OPERATORIO según GRUPO y MOLESTIA MASTICAR pre

| | GRU | | | | | | | | |
|---|---|---|---|---|---|---|---|---|---|
| | Total | | | | | | PE | | |
| | MOLESTIA MASTICAR PRE | | | | | | MOLESTIA MA | | |
| | Total | | Hasta moderada | | Intensa | | Total | | Hasta m |
| | N | % | N | % | N | % | N | % | N |
| Total | 80 | 100,0 % | 34 | 100,0 % | 46 | 100,0 % | 40 | 100,0 % | 14 |
| No | 68 | 85,0 % | 27 | 79,4 % | 41 | 89,1 % | 36 | 90,0 % | 14 |
| Sí | 12 | 15,0 % | 7 | 20,6 % | 5 | 10,9 % | 4 | 10,0 % | 0 |

## T35.- GRADO MOLESTIAS AL MASTICAR PRE según GRUPO y PAA

| | GRUPO | | | | | | | | |
|---|---|---|---|---|---|---|---|---|---|
| | Total | | | PEC | | | POT | | |
| | PAA | | | PAA | | | PAA | | |
| | Total | No | Sí | Total | No | Sí | Total | No | Sí |
| N | 80 | 47 | 33 | 40 | 20 | 20 | 40 | 27 | 13 |

| | | | | | | | | | |
|---|---|---|---|---|---|---|---|---|---|
| Media | 6,5 | 5,3 | 8,2 | 7,2 | 6,2 | 8,2 | 5,8 | 4,6 | 8,4 |
| Desviación típica | 3,3 | 3,4 | 2,0 | 2,7 | 2,8 | 2,2 | 3,6 | 3,7 | 1,8 |
| Mínimo | 0,0 | 0,0 | 2,0 | 0,0 | 0,0 | 2,0 | 0,0 | 0,0 | 4,0 |
| Máximo | 10,0 | 10,0 | 10,0 | 10,0 | 10,0 | 10,0 | 10,0 | 10,0 | 10,0 |
| Percentil 25 | 4,0 | 3,0 | 7,0 | 5,0 | 4,0 | 7,0 | 3,0 | 1,0 | 7,0 |
| Mediana | 7,0 | 5,0 | 9,0 | 8,0 | 6,5 | 9,0 | 6,5 | 4,0 | 9,0 |
| Percentil 75 | 9,0 | 8,0 | 10,0 | 9,0 | 8,5 | 10,0 | 9,5 | 8,0 | 10,0 |

T36.- GRADO NERVIOSISMO PRE según GRUPO y EXPERIENCIA PREVIA

| | GRUPO | | | | | | | | |
|---|---|---|---|---|---|---|---|---|---|
| | Total | | | | PEC | | | | |
| | EXPERIENCIA | | | | EXPERIENCIA | | | | |
| | Total | No | Sí | NS | Total | No | Sí | NS | Total |
| N | 80 | 23 | 50 | 7 | 40 | 14 | 24 | 2 | 40 |
| Media | 3,2 | 2,7 | 3,3 | 4,7 | 3,2 | 2,9 | 3,4 | 2,5 | 3,3 |
| Desviación típica | 3,4 | 2,9 | 3,6 | 3,5 | 3,2 | 2,8 | 3,6 | 3,5 | 3,5 |
| Mínimo | 0,0 | 0,0 | 0,0 | 0,0 | 0,0 | 0,0 | 0,0 | 0,0 | 0,0 |
| Máximo | 10,0 | 7,0 | 10,0 | 9,0 | 10,0 | 7,0 | 10,0 | 5,0 | 10,0 |
| Percentil 25 | 0,0 | 0,0 | 0,0 | 0,0 | 0,0 | 0,0 | 0,0 | 0,0 | 0,0 |
| Mediana | 2,0 | 2,0 | 1,5 | 5,0 | 2,0 | 3,0 | 2,0 | 2,5 | 2,5 |
| Percentil 75 | 6,0 | 6,0 | 6,0 | 8,0 | 5,5 | 5,0 | 6,0 | 5,0 | 6,0 |

T37- EVALUACIÓN INCOMODIDAD TRATAMIENTO (0-ninguna a 10-muy desagradable) según GRUPO y EXPERIENCIA PREVIA

| | | GRUPO | | | | | | | |
|---|---|---|---|---|---|---|---|---|---|
| | | Total | | | | PEC | | | |
| | | EXPERIENCIA | | | | EXPERIENCIA | | | |
| | | Total | No | Sí | NS | Total | No | Sí | NS |
| | N | 80 | 23 | 50 | 7 | 40 | 14 | 24 | 2 |
| | Media | 1,6 | 1,7 | 1,5 | 1,3 | 1,5 | 1,5 | 1,4 | 3,0 |
| | Desviación típica | 1,9 | 2,3 | 1,8 | 1,3 | 2,0 | 2,3 | 1,9 | 0,0 |
| INCOMODIDAD | Mínimo | 0,0 | 0,0 | 0,0 | 0,0 | 0,0 | 0,0 | 0,0 | 3,0 |

| | | | | | | | | | |
|---|---|---|---|---|---|---|---|---|---|
| INCOMODIDAD | Máximo | 7,0 | 7,0 | 5,0 | 3,0 | 7,0 | 7,0 | 5,0 | 3,0 |
| | Percentil 25 | 0,0 | 0,0 | 0,0 | 0,0 | 0,0 | 0,0 | 0,0 | 3,0 |
| | Mediana | 1,0 | 1,0 | 1,0 | 1,0 | 0,0 | 0,5 | 0,0 | 3,0 |
| | Percentil 75 | 3,0 | 3,0 | 3,0 | 3,0 | 3,0 | 2,0 | 2,5 | 3,0 |

T38- EVALUACIÓN SATISFACCIÓN GENERAL TRATAMIENTO (0-nada satisfecho a 10-muy satisfecho) según GRUPO y EXPERIENCIA PREVIA

| | | GRUPO | | | | | | | |
|---|---|---|---|---|---|---|---|---|---|
| | | Total | | | | PEC | | | |
| | | EXPERIENCIA | | | | EXPERIENCIA | | | |
| | | Total | No | Sí | NS | Total | No | Sí | NS |
| SATISFACCION | N | 80 | 23 | 50 | 7 | 40 | 14 | 24 | 2 |
| | Media | 9,1 | 9,1 | 9,0 | 10,0 | 9,2 | 9,1 | 9,2 | 10,0 |
| | Desviación típica | 1,8 | 2,1 | 1,8 | 0,0 | 1,7 | 1,4 | 1,9 | 0,0 |
| | Mínimo | 1,0 | 1,0 | 1,0 | 10,0 | 1,0 | 5,0 | 1,0 | 10,0 |
| | Máximo | 10,0 | 10,0 | 10,0 | 10,0 | 10,0 | 10,0 | 10,0 | 10,0 |
| | Percentil 25 | 9,0 | 9,0 | 8,0 | 10,0 | 9,0 | 9,0 | 9,0 | 10,0 |
| | Mediana | 10,0 | 10,0 | 10,0 | 10,0 | 10,0 | 10,0 | 10,0 | 10,0 |
| | Percentil 75 | 10,0 | 10,0 | 10,0 | 10,0 | 10,0 | 10,0 | 10,0 | 10,0 |

T39- TIEMPO ANESTESIA A APERTURA (minutos) según GRUPO y TIPO ANESTESIA

| | GRUPO | | | | | | | | |
|---|---|---|---|---|---|---|---|---|---|
| | Total | | | PEC | | | POT | | |
| | ANESTESIA | | | ANESTESIA | | | ANESTESIA | | |
| | Total | Infiltrativa | Troncular | Total | Infiltrativa | Troncular | Total | Infiltrativa | Troncular |
| N | 79 | 44 | 35 | 39 | 22 | 17 | 40 | 22 | 18 |
| Media | 16,6 | 15,4 | 18,1 | 19,2 | 18,2 | 20,4 | 14,2 | 12,7 | 16,0 |
| Desviación típica | 10,6 | 10,6 | 10,6 | 11,6 | 12,9 | 10,0 | 9,0 | 6,9 | 11,0 |
| Mínimo | 3,0 | 5,0 | 3,0 | 6,0 | 6,0 | 8,0 | 3,0 | 5,0 | 3,0 |
| Máximo | 63,0 | 63,0 | 50,0 | 63,0 | 63,0 | 47,0 | 50,0 | 30,0 | 50,0 |
| Percentil 25 | 10,0 | 9,5 | 12,0 | 12,0 | 12,0 | 15,0 | 8,5 | 8,0 | 9,0 |
| Mediana | 14,0 | 12,0 | 15,0 | 15,0 | 13,0 | 18,0 | 11,5 | 10,5 | 14,5 |

| | | | | | | | | | |
|---|---|---|---|---|---|---|---|---|---|
| Percentil 75 | 20,0 | 17,0 | 20,0 | 22,0 | 20,0 | 23,0 | 19,0 | 14,0 | 20,0 |

T40- EVOLUCIÓN DOLOR (VAS) según GRUPO y TIEMPO ANESTESIA-APERTURA

| | | GRUPO | | | | | | | |
|---|---|---|---|---|---|---|---|---|---|
| | | Total | | | PEC | | | POT | |
| | | TIEMPO ANESTESIA-APERTURA | | | TIEMPO ANESTESIA-APERTURA | | | TIEMPO ANESTESIA-APERT | |
| | | Total | <15 m | >=15 m | Total | <15 m | >=15 m | Total | <15 m |
| DOLOR pre | N | 79 | 42 | 37 | 39 | 16 | 23 | 40 | 26 |
| | Media | 5,8 | 5,4 | 6,3 | 5,9 | 5,3 | 6,4 | 5,6 | 5,4 |
| | Desviación típica | 2,8 | 3,1 | 2,4 | 2,6 | 3,0 | 2,3 | 3,1 | 3,2 |
| | Mínimo | 0,0 | 0,0 | 0,0 | 0,0 | 0,0 | 1,0 | 0,0 | 0,0 |
| | Máximo | 10,0 | 10,0 | 10,0 | 10,0 | 9,0 | 10,0 | 10,0 | 10,0 |
| | Percentil 25 | 4,0 | 4,0 | 6,0 | 4,0 | 3,5 | 6,0 | 4,0 | 4,0 |
| | Mediana | 7,0 | 6,0 | 7,0 | 7,0 | 6,0 | 7,0 | 6,0 | 5,5 |
| | Percentil 75 | 8,0 | 8,0 | 8,0 | 8,0 | 8,0 | 8,0 | 8,0 | 8,0 |
| DOLOR 6h | N | 79 | 42 | 37 | 39 | 16 | 23 | 40 | 26 |
| | Media | 2,1 | 1,9 | 2,4 | 2,2 | 1,8 | 2,6 | 2,0 | 2,0 |
| | Desviación típica | 2,4 | 2,4 | 2,5 | 2,6 | 2,7 | 2,6 | 2,3 | 2,3 |
| | Mínimo | 0,0 | 0,0 | 0,0 | 0,0 | 0,0 | 0,0 | 0,0 | 0,0 |
| | Máximo | 9,0 | 8,0 | 9,0 | 9,0 | 8,0 | 9,0 | 9,0 | 7,0 |
| | Percentil 25 | 0,0 | 0,0 | 0,0 | 0,0 | 0,0 | 0,0 | 0,0 | 0,0 |
| | Mediana | 2,0 | 1,0 | 2,0 | 2,0 | 0,0 | 2,0 | 1,0 | 1,0 |
| | Percentil 75 | 3,0 | 3,0 | 4,0 | 4,0 | 2,5 | 4,0 | 3,0 | 3,0 |
| DOLOR 24h | N | 79 | 42 | 37 | 39 | 16 | 23 | 40 | 26 |
| | Media | 1,5 | 1,1 | 2,0 | 1,7 | 0,8 | 2,4 | 1,3 | 1,2 |
| | Desviación típica | 2,1 | 1,7 | 2,5 | 2,3 | 1,2 | 2,6 | 2,0 | 1,9 |
| | Mínimo | 0,0 | 0,0 | 0,0 | 0,0 | 0,0 | 0,0 | 0,0 | 0,0 |
| | Máximo | 9,0 | 5,0 | 9,0 | 9,0 | 4,0 | 9,0 | 7,0 | 5,0 |
| | Percentil 25 | 0,0 | 0,0 | 0,0 | 0,0 | 0,0 | 0,0 | 0,0 | 0,0 |
| | Mediana | 1,0 | 0,0 | 1,0 | 1,0 | 0,0 | 2,0 | 0,0 | 0,0 |
| | Percentil 75 | 2,0 | 1,0 | 3,0 | 3,0 | 1,0 | 4,0 | 1,5 | 2,0 |
| | N | 79 | 42 | 37 | 39 | 16 | 23 | 40 | 26 |

| | | | | | | | | | |
|---|---|---|---|---|---|---|---|---|---|
| DOLOR 3d | Media | 1,3 | 1,5 | 1,1 | 1,3 | 1,3 | 1,3 | 1,3 | 1,7 |
| | Desviación típica | 2,0 | 2,4 | 1,6 | 2,1 | 2,6 | 1,7 | 2,0 | 2,2 |
| | Mínimo | 0,0 | 0,0 | 0,0 | 0,0 | 0,0 | 0,0 | 0,0 | 0,0 |
| | Máximo | 10,0 | 10,0 | 6,0 | 10,0 | 10,0 | 6,0 | 10,0 | 10,0 |
| | Percentil 25 | 0,0 | 0,0 | 0,0 | 0,0 | 0,0 | 0,0 | 0,0 | 0,0 |
| | Mediana | 0,0 | 0,0 | 0,0 | 0,0 | 0,0 | 1,0 | 0,5 | 1,0 |
| | Percentil 75 | 2,0 | 3,0 | 2,0 | 2,0 | 2,0 | 2,0 | 2,0 | 3,0 |

## T40b- REDUCCIÓN DOLOR (VAS) 3d - PRE según GRUPO y TIEMPO ANESTESIA-APERTURA

| | | GRUPO | | | | | | | |
|---|---|---|---|---|---|---|---|---|---|
| | | Total | | | PEC | | | POT | |
| | | TIEMPO ANESTESIA-APERTURA | | | TIEMPO ANESTESIA-APERTURA | | | TIEMPO ANESTESIA-APERT | |
| | | Total | <15 m | >=15 m | Total | <15 m | >=15 m | Total | <15 m |
| DIF.DOLOR.3d_PRE | N | 79 | 42 | 37 | 39 | 16 | 23 | 40 | 26 |
| | Media | -4,5 | -3,9 | -5,2 | -4,7 | -4,0 | -5,1 | -4,3 | -3,8 |
| | Desviación típica | 3,5 | 3,9 | 2,9 | 3,2 | 3,8 | 2,7 | 3,8 | 4,0 |
| | Mínimo | -9,0 | -9,0 | -9,0 | -9,0 | -8,0 | -9,0 | -9,0 | -9,0 |
| | Máximo | 6,0 | 6,0 | 1,0 | 4,0 | 4,0 | 1,0 | 6,0 | 6,0 |
| | Percentil 25 | -7,0 | -7,0 | -7,0 | -7,0 | -7,5 | -7,0 | -7,0 | -7,0 |
| | Mediana | -6,0 | -5,0 | -6,0 | -6,0 | -5,0 | -6,0 | -5,0 | -5,0 |
| | Percentil 75 | -1,0 | -1,0 | -4,0 | -1,0 | -1,0 | -3,0 | -1,5 | -1,0 |

## T40c.- TIEMPO HASTA NO DOLOR / DOLOR LEVE según GRUPO y TIEMPO ANESTESIA-APERTURA

| | | | | | | | | | |
|---|---|---|---|---|---|---|---|---|---|
| | | Total | | | | | | | |
| | | TIEMPO ANESTESIA-APERTURA | | | | | | | |
| | | Total | | <15 m | | >=15 m | | Total | |
| | | N | % | N | % | N | % | N | % |
| TIEMPO HASTA NO | Total | 79 | 100,0 % | 42 | 100,0 % | 37 | 100,0 % | 39 | 100,0 % |
| | Pre | 2 | 2,5 % | 2 | 4,8 % | 0 | 0,0 % | 1 | 2,6 % |
| | 6h | 20 | 25,3 % | 14 | 33,3 % | 6 | 16,2 % | 10 | 25,6 % |

| | | | | | | | | | |
|---|---|---|---|---|---|---|---|---|---|
| DOLOR DEFINITIVO | 24h | 7 | 8,9 % | 2 | 4,8 % | 5 | 13,5 % | 2 | 5,1 % |
| | 3d | 11 | 13,9 % | 3 | 7,1 % | 8 | 21,6 % | 8 | 20,5 % |
| | No se alcanza | 39 | 49,4 % | 21 | 50,0 % | 18 | 48,6 % | 18 | 46,2 % |
| TIEMPO HASTA DOLOR LEVE DEFINITIVO | Total | 79 | 100,0 % | 42 | 100,0 % | 37 | 100,0 % | 39 | 100,0 % |
| | Pre | 4 | 5,1 % | 2 | 4,8 % | 2 | 5,4 % | 2 | 5,1 % |
| | 6h | 52 | 65,8 % | 29 | 69,0 % | 23 | 62,2 % | 23 | 59,0 % |
| | 24h | 7 | 8,9 % | 3 | 7,1 % | 4 | 10,8 % | 5 | 12,8 % |
| | 3d | 7 | 8,9 % | 3 | 7,1 % | 4 | 10,8 % | 4 | 10,3 % |
| | No se alcanza | 9 | 11,4 % | 5 | 11,9 % | 4 | 10,8 % | 5 | 12,8 % |

T41- EVALUACIÓN DURACIÓN TRATAMIENTO (0-muy corto a 10-demasiado largo) según GRUPO y TIEMPO ANESTESIA-APERTURA

| | | GRUPO | | | | | | | |
|---|---|---|---|---|---|---|---|---|---|
| | | Total | | | PEC | | | POT | |
| | | TIEMPO ANESTESIA-APERTURA | | | TIEMPO ANESTESIA-APERTURA | | | TIEMPO ANESTESIA-APERT | |
| | | Total | <15 m | >=15 m | Total | <15 m | >=15 m | Total | <15 m |
| DURACION | N | 79 | 42 | 37 | 39 | 16 | 23 | 40 | 26 |
| | Media | 2,5 | 2,6 | 2,4 | 3,1 | 3,2 | 3,0 | 2,0 | 2,3 |
| | Desviación típica | 1,8 | 1,9 | 1,7 | 1,9 | 2,1 | 1,8 | 1,6 | 1,7 |
| | Mínimo | 0,0 | 0,0 | 0,0 | 0,0 | 1,0 | 0,0 | 0,0 | 0,0 |
| | Máximo | 10,0 | 10,0 | 7,0 | 10,0 | 10,0 | 7,0 | 5,0 | 5,0 |
| | Percentil 25 | 1,0 | 2,0 | 1,0 | 2,0 | 2,0 | 2,0 | 0,0 | 0,0 |
| | Mediana | 3,0 | 3,0 | 3,0 | 3,0 | 3,0 | 3,0 | 3,0 | 3,0 |
| | Percentil 75 | 3,0 | 3,0 | 3,0 | 3,0 | 3,0 | 4,0 | 3,0 | 3,0 |

T42- EVALUACIÓN INCOMODIDAD TRATAMIENTO (0-muy corto a 10-demasiado largo) según GRUPO y TIEMPO ANESTESIA-APERTURA

| | | GRUPO | | | | | | | |
|---|---|---|---|---|---|---|---|---|---|
| | | Total | | | PEC | | | POT | |
| | | TIEMPO ANESTESIA-APERTURA | | | TIEMPO ANESTESIA-APERTURA | | | TIEMPO ANESTESIA-APERT | |
| | | Total | <15 m | >=15 m | Total | <15 m | >=15 m | Total | <15 m |
| | N | 79 | 42 | 37 | 39 | 16 | 23 | 40 | 26 |
| | Media | 1,5 | 1,4 | 1,7 | 1,5 | 1,6 | 1,4 | 1,6 | 1,3 |

| | | | | | | | | | |
|---|---|---|---|---|---|---|---|---|---|
| INCOMODIDAD | Desviación típica | 1,9 | 1,9 | 2,0 | 2,0 | 2,3 | 1,9 | 1,8 | 1,5 |
| | Mínimo | 0,0 | 0,0 | 0,0 | 0,0 | 0,0 | 0,0 | 0,0 | 0,0 |
| | Máximo | 7,0 | 7,0 | 6,0 | 7,0 | 7,0 | 5,0 | 6,0 | 5,0 |
| | Percentil 25 | 0,0 | 0,0 | 0,0 | 0,0 | 0,0 | 0,0 | 0,0 | 0,0 |
| | Mediana | 1,0 | 1,0 | 1,0 | 0,0 | 0,5 | 0,0 | 1,0 | 1,0 |
| | Percentil 75 | 3,0 | 2,0 | 3,0 | 3,0 | 2,0 | 3,0 | 3,0 | 2,0 |

T43- EVALUACIÓN SATISFACCIÓN GENERAL TRATAMIENTO (0-nada satisfecho a 10-muy satisfecho) según GRUPO y TIEMPO ANESTESIA-APERTURA

| | | GRUPO | | | | | | | |
|---|---|---|---|---|---|---|---|---|---|
| | | Total | | | PEC | | | POT | |
| | | TIEMPO ANESTESIA-APERTURA | | | TIEMPO ANESTESIA-APERTURA | | | TIEMPO ANESTESIA-APERT | |
| | | Total | <15 m | >=15 m | Total | <15 m | >=15 m | Total | <15 m |
| SATISFACCION | N | 79 | 42 | 37 | 39 | 16 | 23 | 40 | 26 |
| | Media | 9,1 | 9,2 | 9,0 | 9,2 | 9,0 | 9,3 | 9,1 | 9,3 |
| | Desviación típica | 1,8 | 1,7 | 2,0 | 1,7 | 2,3 | 1,2 | 2,0 | 1,3 |
| | Mínimo | 1,0 | 1,0 | 1,0 | 1,0 | 1,0 | 5,0 | 1,0 | 6,0 |
| | Máximo | 10,0 | 10,0 | 10,0 | 10,0 | 10,0 | 10,0 | 10,0 | 10,0 |
| | Percentil 25 | 9,0 | 9,0 | 9,0 | 9,0 | 8,5 | 9,0 | 9,0 | 9,0 |
| | Mediana | 10,0 | 10,0 | 10,0 | 10,0 | 10,0 | 10,0 | 10,0 | 10,0 |
| | Percentil 75 | 10,0 | 10,0 | 10,0 | 10,0 | 10,0 | 10,0 | 10,0 | 10,0 |

T44- EVOLUCIÓN DOLOR (VAS) según GRUPO y TIEMPO APERTURA-FINAL

| | | GRUPO | | | | | | | |
|---|---|---|---|---|---|---|---|---|---|
| | | Total | | | PEC | | | POT | |
| | | TIEMPO APERTURA-FINAL | | | TIEMPO APERTURA-FINAL | | | TIEMPO APERTURA-FINA | |
| | | Total | <=8 m | >8 m | Total | <=8 m | >8 m | Total | <=8 m |
| DOLOR pre | N | 79 | 41 | 38 | 39 | 10 | 29 | 40 | 31 |
| | Media | 5,8 | 6,2 | 5,4 | 5,9 | 6,7 | 5,7 | 5,6 | 6,0 |
| | Desviación típica | 2,8 | 2,5 | 3,1 | 2,6 | 1,6 | 2,9 | 3,1 | 2,8 |
| | Mínimo | 0,0 | 0,0 | 0,0 | 0,0 | 4,0 | 0,0 | 0,0 | 0,0 |
| | Máximo | 10,0 | 10,0 | 10,0 | 10,0 | 8,0 | 10,0 | 10,0 | 10,0 |

| | | | | | | | | | |
|---|---|---|---|---|---|---|---|---|---|
| | Percentil 25 | 4,0 | 5,0 | 3,0 | 4,0 | 6,0 | 3,0 | 4,0 | 5,0 |
| | Mediana | 7,0 | 7,0 | 6,0 | 7,0 | 7,0 | 6,0 | 6,0 | 6,0 |
| | Percentil 75 | 8,0 | 8,0 | 8,0 | 8,0 | 8,0 | 8,0 | 8,0 | 8,0 |
| DOLOR 6h | N | 79 | 41 | 38 | 39 | 10 | 29 | 40 | 31 |
| | Media | 2,1 | 2,0 | 2,3 | 2,2 | 2,8 | 2,0 | 2,0 | 1,7 |
| | Desviación típica | 2,4 | 2,4 | 2,5 | 2,6 | 3,0 | 2,5 | 2,3 | 2,1 |
| | Mínimo | 0,0 | 0,0 | 0,0 | 0,0 | 0,0 | 0,0 | 0,0 | 0,0 |
| | Máximo | 9,0 | 9,0 | 9,0 | 9,0 | 8,0 | 9,0 | 9,0 | 9,0 |
| | Percentil 25 | 0,0 | 0,0 | 0,0 | 0,0 | 0,0 | 0,0 | 0,0 | 0,0 |
| | Mediana | 2,0 | 1,0 | 2,0 | 2,0 | 2,0 | 1,0 | 1,0 | 1,0 |
| | Percentil 75 | 3,0 | 3,0 | 4,0 | 4,0 | 3,0 | 4,0 | 3,0 | 3,0 |
| DOLOR 24h | N | 79 | 41 | 38 | 39 | 10 | 29 | 40 | 31 |
| | Media | 1,5 | 0,9 | 2,1 | 1,7 | 1,1 | 2,0 | 1,3 | 0,9 |
| | Desviación típica | 2,1 | 1,9 | 2,2 | 2,3 | 2,5 | 2,2 | 2,0 | 1,7 |
| | Mínimo | 0,0 | 0,0 | 0,0 | 0,0 | 0,0 | 0,0 | 0,0 | 0,0 |
| | Máximo | 9,0 | 8,0 | 9,0 | 9,0 | 8,0 | 9,0 | 7,0 | 7,0 |
| | Percentil 25 | 0,0 | 0,0 | 0,0 | 0,0 | 0,0 | 0,0 | 0,0 | 0,0 |
| | Mediana | 1,0 | 0,0 | 1,5 | 1,0 | 0,0 | 2,0 | 0,0 | 0,0 |
| | Percentil 75 | 2,0 | 1,0 | 4,0 | 3,0 | 1,0 | 3,0 | 1,5 | 1,0 |
| DOLOR 3d | N | 79 | 41 | 38 | 39 | 10 | 29 | 40 | 31 |
| | Media | 1,3 | 1,0 | 1,6 | 1,3 | 1,2 | 1,3 | 1,3 | 1,0 |
| | Desviación típica | 2,0 | 1,4 | 2,5 | 2,1 | 1,7 | 2,3 | 2,0 | 1,4 |
| | Mínimo | 0,0 | 0,0 | 0,0 | 0,0 | 0,0 | 0,0 | 0,0 | 0,0 |
| | Máximo | 10,0 | 5,0 | 10,0 | 10,0 | 5,0 | 10,0 | 10,0 | 5,0 |
| | Percentil 25 | 0,0 | 0,0 | 0,0 | 0,0 | 0,0 | 0,0 | 0,0 | 0,0 |
| | Mediana | 0,0 | 0,0 | 0,5 | 0,0 | 0,5 | 0,0 | 0,5 | 0,0 |
| | Percentil 75 | 2,0 | 2,0 | 2,0 | 2,0 | 2,0 | 2,0 | 2,0 | 2,0 |

## T44b- REDUCCIÓN DOLOR (VAS) 3d - PRE según GRUPO y TIEMPO APERTURA-FINAL

| | GRUPO | | |
|---|---|---|---|
| | Total | PEC | POT |
| | TIEMPO APERTURA-FINAL | TIEMPO APERTURA-FINAL | TIEMPO APERTURA-FINA |

| | | Total | <=8 m | >8 m | Total | <=8 m | >8 m | Total | <=8 m |
|---|---|---|---|---|---|---|---|---|---|
| DIF.DOLOR.3d_PRE | N | 79 | 41 | 38 | 39 | 10 | 29 | 40 | 31 |
| | Media | -4,5 | -5,1 | -3,8 | -4,7 | -5,5 | -4,4 | -4,3 | -5,0 |
| | Desviación típica | 3,5 | 2,9 | 4,0 | 3,2 | 2,5 | 3,4 | 3,8 | 3,0 |
| | Mínimo | -9,0 | -9,0 | -9,0 | -9,0 | -8,0 | -9,0 | -9,0 | -9,0 |
| | Máximo | 6,0 | 3,0 | 6,0 | 4,0 | -1,0 | 4,0 | 6,0 | 3,0 |
| | Percentil 25 | -7,0 | -7,0 | -7,0 | -7,0 | -8,0 | -7,0 | -7,0 | -7,0 |
| | Mediana | -6,0 | -6,0 | -5,5 | -6,0 | -6,0 | -6,0 | -5,0 | -6,0 |
| | Percentil 75 | -1,0 | -4,0 | -1,0 | -1,0 | -4,0 | -1,0 | -1,5 | -3,0 |

T44c.- TIEMPO HASTA NO DOLOR / DOLOR LEVE según GRUPO y TIEMPO APERTURA-FINAL

| | | | | | | | | | |
|---|---|---|---|---|---|---|---|---|---|
| | | Total | | | | | | | |
| | | TIEMPO APERTURA-FINAL | | | | | | | |
| | | Total | | <=8 m | | >8 m | | Total | |
| | | N | % | N | % | N | % | N | % |
| TIEMPO HASTA NO DOLOR DEFINITIVO | Total | 79 | 100,0 % | 41 | 100,0 % | 38 | 100,0 % | 39 | 100,0 % |
| | Pre | 2 | 2,5 % | 0 | 0,0 % | 2 | 5,3 % | 1 | 2,6 % |
| | 6h | 20 | 25,3 % | 13 | 31,7 % | 7 | 18,4 % | 10 | 25,6 % |
| | 24h | 7 | 8,9 % | 5 | 12,2 % | 2 | 5,3 % | 2 | 5,1 % |
| | 3d | 11 | 13,9 % | 4 | 9,8 % | 7 | 18,4 % | 8 | 20,5 % |
| | No se alcanza | 39 | 49,4 % | 19 | 46,3 % | 20 | 52,6 % | 18 | 46,2 % |
| TIEMPO HASTA DOLOR LEVE DEFINITIVO | Total | 79 | 100,0 % | 41 | 100,0 % | 38 | 100,0 % | 39 | 100,0 % |
| | Pre | 4 | 5,1 % | 1 | 2,4 % | 3 | 7,9 % | 2 | 5,1 % |
| | 6h | 52 | 65,8 % | 33 | 80,5 % | 19 | 50,0 % | 23 | 59,0 % |
| | 24h | 7 | 8,9 % | 4 | 9,8 % | 3 | 7,9 % | 5 | 12,8 % |
| | 3d | 7 | 8,9 % | 1 | 2,4 % | 6 | 15,8 % | 4 | 10,3 % |
| | No se alcanza | 9 | 11,4 % | 2 | 4,9 % | 7 | 18,4 % | 5 | 12,8 % |

T45- EVALUACIÓN DURACIÓN TRATAMIENTO (0-muy corto a 10-demasiado largo) según GRUPO y TIEMPO APERTURA-FINAL

| | GRUPO |
|---|---|

| | | Total | | | PEC | | | POT | |
|---|---|---|---|---|---|---|---|---|---|
| | | TIEMPO APERTURA-FINAL | | | TIEMPO APERTURA-FINAL | | | TIEMPO APERTURA-FIN/ | |
| | | Total | <=8 m | >8 m | Total | <=8 m | >8 m | Total | <=8 m |
| DURACION | N | 79 | 41 | 38 | 39 | 10 | 29 | 40 | 31 |
| | Media | 2,5 | 2,1 | 3,0 | 3,1 | 2,7 | 3,2 | 2,0 | 1,9 |
| | Desviación típica | 1,8 | 1,9 | 1,6 | 1,9 | 2,9 | 1,4 | 1,6 | 1,5 |
| | Mínimo | 0,0 | 0,0 | 0,0 | 0,0 | 0,0 | 0,0 | 0,0 | 0,0 |
| | Máximo | 10,0 | 10,0 | 7,0 | 10,0 | 10,0 | 7,0 | 5,0 | 5,0 |
| | Percentil 25 | 1,0 | 1,0 | 2,0 | 2,0 | 1,0 | 3,0 | 0,0 | 0,0 |
| | Mediana | 3,0 | 2,0 | 3,0 | 3,0 | 2,0 | 3,0 | 3,0 | 2,0 |
| | Percentil 75 | 3,0 | 3,0 | 3,0 | 3,0 | 3,0 | 3,0 | 3,0 | 3,0 |

T46- EVALUACIÓN INCOMODIDAD TRATAMIENTO (0-muy corto a 10-demasiado largo) según GRUPO y TIEMPO APERTURA-FINAL

| | | GRUPO | | | | | | | |
|---|---|---|---|---|---|---|---|---|---|
| | | Total | | | PEC | | | POT | |
| | | TIEMPO APERTURA-FINAL | | | TIEMPO APERTURA-FINAL | | | TIEMPO APERTURA-FIN/ | |
| | | Total | <=8 m | >8 m | Total | <=8 m | >8 m | Total | <=8 m |
| INCOMODIDAD | N | 79 | 41 | 38 | 39 | 10 | 29 | 40 | 31 |
| | Media | 1,5 | 1,4 | 1,7 | 1,5 | 1,2 | 1,6 | 1,6 | 1,5 |
| | Desviación típica | 1,9 | 2,0 | 1,9 | 2,0 | 2,3 | 2,0 | 1,8 | 1,9 |
| | Mínimo | 0,0 | 0,0 | 0,0 | 0,0 | 0,0 | 0,0 | 0,0 | 0,0 |
| | Máximo | 7,0 | 6,0 | 7,0 | 7,0 | 6,0 | 7,0 | 6,0 | 6,0 |
| | Percentil 25 | 0,0 | 0,0 | 0,0 | 0,0 | 0,0 | 0,0 | 0,0 | 0,0 |
| | Mediana | 1,0 | 1,0 | 1,0 | 0,0 | 0,0 | 1,0 | 1,0 | 1,0 |
| | Percentil 75 | 3,0 | 2,0 | 3,0 | 3,0 | 1,0 | 3,0 | 3,0 | 3,0 |

T47- EVALUACIÓN SATISFACCIÓN GENERAL TRATAMIENTO (0-nada satisfecho a 10-muy satisfecho) según GRUPO y TIEMPO APERTURA-FINAL

| | GRUPO | | | | | | | |
|---|---|---|---|---|---|---|---|---|
| | Total | | | PEC | | | POT | |
| | TIEMPO APERTURA-FINAL | | | TIEMPO APERTURA-FINAL | | | TIEMPO APERTURA-FIN/ | |
| | Total | <=8 m | >8 m | Total | <=8 m | >8 m | Total | <=8 m |

| | | | | | | | | | |
|---|---|---|---|---|---|---|---|---|---|
| SATISFACCION | N | 79 | 41 | 38 | 39 | 10 | 29 | 40 | 31 |
| | Media | 9,1 | 9,3 | 8,9 | 9,2 | 9,7 | 9,0 | 9,1 | 9,2 |
| | Desviación típica | 1,8 | 1,7 | 2,0 | 1,7 | 0,7 | 1,9 | 2,0 | 1,9 |
| | Mínimo | 1,0 | 1,0 | 1,0 | 1,0 | 8,0 | 1,0 | 1,0 | 1,0 |
| | Máximo | 10,0 | 10,0 | 10,0 | 10,0 | 10,0 | 10,0 | 10,0 | 10,0 |
| | Percentil 25 | 9,0 | 10,0 | 8,0 | 9,0 | 10,0 | 9,0 | 9,0 | 10,0 |
| | Mediana | 10,0 | 10,0 | 10,0 | 10,0 | 10,0 | 10,0 | 10,0 | 10,0 |
| | Percentil 75 | 10,0 | 10,0 | 10,0 | 10,0 | 10,0 | 10,0 | 10,0 | 10,0 |

## T48- EVOLUCIÓN DOLOR (VAS) según GRUPO y SANGRADO PROFUSO

| | | GRUPO | | | | | | | |
|---|---|---|---|---|---|---|---|---|---|
| | | Total | | | PEC | | | POT | |
| | | SANGRADO PROFUSO | | | SANGRADO PROFUSO | | | SANGRADO PROFUSO | |
| | | Total | No | Sí | Total | No | Sí | Total | No |
| DOLOR pre | N | 80 | 51 | 29 | 40 | 24 | 16 | 40 | 27 |
| | Media | 5,8 | 6,1 | 5,2 | 6,0 | 6,3 | 5,4 | 5,6 | 6,0 |
| | Desviación típica | 2,8 | 2,5 | 3,2 | 2,6 | 2,1 | 3,2 | 3,1 | 2,9 |
| | Mínimo | 0,0 | 0,0 | 0,0 | 0,0 | 1,0 | 0,0 | 0,0 | 0,0 |
| | Máximo | 10,0 | 10,0 | 10,0 | 10,0 | 9,0 | 10,0 | 10,0 | 10,0 |
| | Percentil 25 | 4,0 | 5,0 | 2,0 | 4,0 | 6,0 | 3,0 | 4,0 | 5,0 |
| | Mediana | 6,5 | 7,0 | 6,0 | 7,0 | 6,5 | 7,0 | 6,0 | 7,0 |
| | Percentil 75 | 8,0 | 8,0 | 8,0 | 8,0 | 8,0 | 8,0 | 8,0 | 8,0 |
| DOLOR 6h | N | 80 | 51 | 29 | 40 | 24 | 16 | 40 | 27 |
| | Media | 2,1 | 2,0 | 2,3 | 2,2 | 2,3 | 2,1 | 2,0 | 1,7 |
| | Desviación típica | 2,4 | 2,3 | 2,7 | 2,6 | 2,6 | 2,7 | 2,3 | 1,9 |
| | Mínimo | 0,0 | 0,0 | 0,0 | 0,0 | 0,0 | 0,0 | 0,0 | 0,0 |
| | Máximo | 9,0 | 9,0 | 9,0 | 9,0 | 9,0 | 8,0 | 9,0 | 7,0 |
| | Percentil 25 | 0,0 | 0,0 | 0,0 | 0,0 | 0,0 | 0,0 | 0,0 | 0,0 |
| | Mediana | 2,0 | 2,0 | 1,0 | 2,0 | 2,0 | 0,5 | 1,0 | 1,0 |
| | Percentil 75 | 3,0 | 3,0 | 4,0 | 3,5 | 3,0 | 4,0 | 3,0 | 3,0 |
| | N | 80 | 51 | 29 | 40 | 24 | 16 | 40 | 27 |
| | Media | 1,5 | 1,5 | 1,4 | 1,8 | 2,1 | 1,3 | 1,3 | 1,0 |

| | | | | | | | | | |
|---|---|---|---|---|---|---|---|---|---|
| DOLOR 24h | Desviación típica | 2,1 | 2,1 | 2,1 | 2,3 | 2,4 | 2,0 | 2,0 | 1,7 |
| | Mínimo | 0,0 | 0,0 | 0,0 | 0,0 | 0,0 | 0,0 | 0,0 | 0,0 |
| | Máximo | 9,0 | 9,0 | 7,0 | 9,0 | 9,0 | 7,0 | 7,0 | 5,0 |
| | Percentil 25 | 0,0 | 0,0 | 0,0 | 0,0 | 0,0 | 0,0 | 0,0 | 0,0 |
| | Mediana | 1,0 | 1,0 | 0,0 | 1,0 | 1,5 | 0,0 | 0,0 | 0,0 |
| | Percentil 75 | 2,0 | 2,0 | 2,0 | 2,5 | 3,0 | 2,0 | 1,5 | 1,0 |
| DOLOR 3d | N | 80 | 51 | 29 | 40 | 24 | 16 | 40 | 27 |
| | Media | 1,3 | 1,4 | 1,2 | 1,3 | 1,5 | 0,8 | 1,3 | 1,2 |
| | Desviación típica | 2,0 | 2,0 | 2,0 | 2,1 | 2,5 | 1,0 | 2,0 | 1,5 |
| | Mínimo | 0,0 | 0,0 | 0,0 | 0,0 | 0,0 | 0,0 | 0,0 | 0,0 |
| | Máximo | 10,0 | 10,0 | 10,0 | 10,0 | 10,0 | 3,0 | 10,0 | 5,0 |
| | Percentil 25 | 0,0 | 0,0 | 0,0 | 0,0 | 0,0 | 0,0 | 0,0 | 0,0 |
| | Mediana | 0,0 | 0,0 | 0,0 | 0,0 | 0,0 | 0,0 | 0,5 | 0,0 |
| | Percentil 75 | 2,0 | 2,0 | 2,0 | 2,0 | 2,5 | 2,0 | 2,0 | 2,0 |

## T49b- REDUCCIÓN DOLOR (VAS) 3d - PRE según GRUPO y SANGRADO PROFUSO

| | | GRUPO | | | | | | | |
|---|---|---|---|---|---|---|---|---|---|
| | | Total | | | PEC | | | POT | |
| | | SANGRADO PROFUSO | | | SANGRADO PROFUSO | | | SANGRADO PROFUSO | |
| | | Total | No | Sí | Total | No | Sí | Total | No |
| DIF.DOLOR.3d_PRE | N | 80 | 51 | 29 | 40 | 24 | 16 | 40 | 27 |
| | Media | -4,5 | -4,8 | -4,0 | -4,7 | -4,8 | -4,6 | -4,3 | -4,8 |
| | Desviación típica | 3,5 | 3,1 | 4,1 | 3,2 | 3,3 | 3,1 | 3,8 | 3,1 |
| | Mínimo | -9,0 | -9,0 | -9,0 | -9,0 | -9,0 | -9,0 | -9,0 | -8,0 |
| | Máximo | 6,0 | 4,0 | 6,0 | 4,0 | 4,0 | 0,0 | 6,0 | 3,0 |
| | Percentil 25 | -7,0 | -7,0 | -8,0 | -7,0 | -7,0 | -7,5 | -7,0 | -7,0 |
| | Mediana | -6,0 | -6,0 | -5,0 | -6,0 | -6,0 | -5,0 | -5,0 | -5,0 |
| | Percentil 75 | -1,5 | -3,0 | -1,0 | -1,5 | -2,5 | -1,0 | -1,5 | -3,0 |

## T49c.- TIEMPO HASTA NO DOLOR / DOLOR LEVE según GRUPO y SANGRADO PROFUSO

| | | Total | | | | | | | |
|---|---|---|---|---|---|---|---|---|---|
| | | SANGRADO PROFUSO | | | | | | | |
| | | Total | | No | | Sí | | Total | |
| | | N | % | N | % | N | % | N | % |
| TIEMPO HASTA NO DOLOR DEFINITIVO | Total | 80 | 100,0 % | 51 | 100,0 % | 29 | 100,0 % | 40 | 100,0 % |
| | Pre | 2 | 2,5 % | 1 | 2,0 % | 1 | 3,4 % | 1 | 2,5 % |
| | 6h | 20 | 25,0 % | 11 | 21,6 % | 9 | 31,0 % | 10 | 25,0 % |
| | 24h | 7 | 8,8 % | 6 | 11,8 % | 1 | 3,4 % | 2 | 5,0 % |
| | 3d | 12 | 15,0 % | 9 | 17,6 % | 3 | 10,3 % | 9 | 22,5 % |
| | No se alcanza | 39 | 48,8 % | 24 | 47,1 % | 15 | 51,7 % | 18 | 45,0 % |
| TIEMPO HASTA DOLOR LEVE DEFINITIVO | Total | 80 | 100,0 % | 51 | 100,0 % | 29 | 100,0 % | 40 | 100,0 % |
| | Pre | 4 | 5,0 % | 3 | 5,9 % | 1 | 3,4 % | 2 | 5,0 % |
| | 6h | 53 | 66,3 % | 35 | 68,6 % | 18 | 62,1 % | 24 | 60,0 % |
| | 24h | 7 | 8,8 % | 2 | 3,9 % | 5 | 17,2 % | 5 | 12,5 % |
| | 3d | 7 | 8,8 % | 4 | 7,8 % | 3 | 10,3 % | 4 | 10,0 % |
| | No se alcanza | 9 | 11,3 % | 7 | 13,7 % | 2 | 6,9 % | 5 | 12,5 % |

T50- EVALUACIÓN SATISFACCIÓN GENERAL TRATAMIENTO (0-nada satisfecho a 10-muy satisfecho) según GRUPO y SANGRADO PROFUSO

| | | GRUPO | | | | | | | |
|---|---|---|---|---|---|---|---|---|---|
| | | Total | | | PEC | | | POT | |
| | | SANGRADO PROFUSO | | | SANGRADO PROFUSO | | | SANGRADO PROFUSO | |
| | | Total | No | Sí | Total | No | Sí | Total | No |
| SATISFACCION | N | 80 | 51 | 29 | 40 | 24 | 16 | 40 | 27 |
| | Media | 9,1 | 8,9 | 9,4 | 9,2 | 9,0 | 9,4 | 9,1 | 8,9 |
| | Desviación típica | 1,8 | 2,2 | 0,9 | 1,7 | 2,1 | 0,8 | 2,0 | 2,3 |
| | Mínimo | 1,0 | 1,0 | 7,0 | 1,0 | 1,0 | 8,0 | 1,0 | 1,0 |
| | Máximo | 10,0 | 10,0 | 10,0 | 10,0 | 10,0 | 10,0 | 10,0 | 10,0 |
| | Percentil 25 | 9,0 | 9,0 | 9,0 | 9,0 | 9,0 | 9,0 | 9,0 | 9,0 |
| | Mediana | 10,0 | 10,0 | 10,0 | 10,0 | 10,0 | 10,0 | 10,0 | 10,0 |
| | Percentil 75 | 10,0 | 10,0 | 10,0 | 10,0 | 10,0 | 10,0 | 10,0 | 10,0 |

## T51.- SANGRADO PROFUSO según GRUPO y CAUSA

| | Total | | | | | | | | |
|---|---|---|---|---|---|---|---|---|---|
| | CAUSA | | | | | | | | |
| | Total | | Caries | | Obturación | | Resto | | To |
| | N | % | N | % | N | % | N | % | N |
| **Total** | 80 | 100,0 % | 40 | 100,0 % | 23 | 100,0 % | 17 | 100,0 % | 40 |
| **No** | 51 | 63,7 % | 27 | 67,5 % | 16 | 69,6 % | 8 | 47,1 % | 24 |
| **Sí** | 29 | 36,3 % | 13 | 32,5 % | 7 | 30,4 % | 9 | 52,9 % | 16 |

## T52.- SANGRADO CONDUCTO según GRUPO y CAUSA

| | Total | | | | | | | | |
|---|---|---|---|---|---|---|---|---|---|
| | CAUSA | | | | | | | | |
| | Total | | Caries | | Obturación | | Resto | | To |
| | N | % | N | % | N | % | N | % | N |
| **Total** | 80 | 100,0 % | 40 | 100,0 % | 23 | 100,0 % | 17 | 100,0 % | 40 |
| **No** | 59 | 73,8 % | 29 | 72,5 % | 18 | 78,3 % | 12 | 70,6 % | 28 |
| **Sí** | 21 | 26,3 % | 11 | 27,5 % | 5 | 21,7 % | 5 | 29,4 % | 12 |

## T53.- PAA PREVIO según GRUPO y CAUSA

| | Total | | | | | | | | |
|---|---|---|---|---|---|---|---|---|---|
| | CAUSA | | | | | | | | |
| | Total | | Caries | | Obturación | | Resto | | To |
| | N | % | N | % | N | % | N | % | N |
| **Total** | 80 | 100,0 % | 40 | 100,0 % | 23 | 100,0 % | 17 | 100,0 % | 40 |
| **No** | 47 | 58,8 % | 24 | 60,0 % | 11 | 47,8 % | 12 | 70,6 % | 20 |
| **Sí** | 33 | 41,3 % | 16 | 40,0 % | 12 | 52,2 % | 5 | 29,4 % | 20 |

T54- EVOLUCIÓN DOLOR (VAS) según GRUPO y CAUSA

| | | GRUPO | | | | | | | |
|---|---|---|---|---|---|---|---|---|---|
| | | Total | | | | PEC | | | |
| | | CAUSA | | | | CAUSA | | | |
| | | Total | Caries | Obturación | Resto | Total | Caries | Obturación | Resto |
| DOLOR pre | N | 80 | 40 | 23 | 17 | 40 | 19 | 13 | 8 |
| | Media | 5,8 | 5,2 | 6,6 | 6,1 | 6,0 | 5,3 | 7,5 | 5,0 |
| | Desviación típica | 2,8 | 2,9 | 2,4 | 3,0 | 2,6 | 2,8 | 1,5 | 2,6 |
| | Mínimo | 0,0 | 0,0 | 0,0 | 0,0 | 0,0 | 0,0 | 4,0 | 0,0 |
| | Máximo | 10,0 | 9,0 | 10,0 | 10,0 | 10,0 | 9,0 | 10,0 | 8,0 |
| | Percentil 25 | 4,0 | 3,0 | 6,0 | 5,0 | 4,0 | 3,0 | 7,0 | 3,5 |
| | Mediana | 6,5 | 6,0 | 7,0 | 6,0 | 7,0 | 6,0 | 7,0 | 6,0 |
| | Percentil 75 | 8,0 | 8,0 | 8,0 | 8,0 | 8,0 | 8,0 | 8,0 | 6,5 |
| DOLOR 6h | N | 80 | 40 | 23 | 17 | 40 | 19 | 13 | 8 |
| | Media | 2,1 | 2,0 | 2,6 | 1,8 | 2,2 | 1,7 | 3,3 | 1,6 |
| | Desviación típica | 2,4 | 2,3 | 2,4 | 2,8 | 2,6 | 2,6 | 2,3 | 2,8 |
| | Mínimo | 0,0 | 0,0 | 0,0 | 0,0 | 0,0 | 0,0 | 0,0 | 0,0 |
| | Máximo | 9,0 | 9,0 | 8,0 | 9,0 | 9,0 | 9,0 | 8,0 | 8,0 |
| | Percentil 25 | 0,0 | 0,0 | 0,0 | 0,0 | 0,0 | 0,0 | 2,0 | 0,0 |
| | Mediana | 2,0 | 1,5 | 2,0 | 0,0 | 2,0 | 0,0 | 3,0 | 0,0 |
| | Percentil 75 | 3,0 | 3,0 | 4,0 | 3,0 | 3,5 | 3,0 | 4,0 | 2,5 |
| DOLOR 24h | N | 80 | 40 | 23 | 17 | 40 | 19 | 13 | 8 |
| | Media | 1,5 | 1,5 | 1,7 | 1,3 | 1,8 | 1,6 | 2,3 | 1,1 |
| | Desviación típica | 2,1 | 2,1 | 2,1 | 2,1 | 2,3 | 2,5 | 2,3 | 1,5 |
| | Mínimo | 0,0 | 0,0 | 0,0 | 0,0 | 0,0 | 0,0 | 0,0 | 0,0 |
| | Máximo | 9,0 | 9,0 | 8,0 | 7,0 | 9,0 | 9,0 | 8,0 | 4,0 |
| | Percentil 25 | 0,0 | 0,0 | 0,0 | 0,0 | 0,0 | 0,0 | 0,0 | 0,0 |
| | Mediana | 1,0 | 1,0 | 1,0 | 0,0 | 1,0 | 1,0 | 2,0 | 0,5 |
| | Percentil 75 | 2,0 | 2,0 | 3,0 | 2,0 | 2,5 | 2,0 | 3,0 | 2,0 |
| | N | 80 | 40 | 23 | 17 | 40 | 19 | 13 | 8 |
| | Media | 1,3 | 0,8 | 2,1 | 1,4 | 1,3 | 0,5 | 1,8 | 2,1 |
| | Desviación típica | 2,0 | 1,1 | 2,4 | 2,7 | 2,1 | 0,8 | 2,0 | 3,6 |

| | | | | | | | | | |
|---|---|---|---|---|---|---|---|---|---|
| DOLOR 3d | Mínimo | 0,0 | 0,0 | 0,0 | 0,0 | 0,0 | 0,0 | 0,0 | 0,0 |
| | Máximo | 10,0 | 4,0 | 10,0 | 10,0 | 10,0 | 2,0 | 6,0 | 10,0 |
| | Percentil 25 | 0,0 | 0,0 | 0,0 | 0,0 | 0,0 | 0,0 | 0,0 | 0,0 |
| | Mediana | 0,0 | 0,0 | 2,0 | 0,0 | 0,0 | 0,0 | 1,0 | 0,0 |
| | Percentil 75 | 2,0 | 1,5 | 3,0 | 1,0 | 2,0 | 1,0 | 3,0 | 3,5 |

T54b- REDUCCIÓN DOLOR (VAS) 3d - PRE según GRUPO y CAUSA

| | | GRUPO | | | | | | | |
|---|---|---|---|---|---|---|---|---|---|
| | | Total | | | | PEC | | | |
| | | CAUSA | | | | CAUSA | | | |
| | | Total | Caries | Obturación | Resto | Total | Caries | Obturación | Resto |
| DIF.DOLOR.3d_PRE | N | 80 | 40 | 23 | 17 | 40 | 19 | 13 | 8 |
| | Media | -4,5 | -4,4 | -4,5 | -4,6 | -4,7 | -4,8 | -5,6 | -2,9 |
| | Desviación típica | 3,5 | 3,2 | 3,8 | 3,9 | 3,2 | 2,7 | 2,7 | 4,4 |
| | Mínimo | -9,0 | -9,0 | -9,0 | -9,0 | -9,0 | -9,0 | -9,0 | -8,0 |
| | Máximo | 6,0 | 4,0 | 6,0 | 4,0 | 4,0 | 0,0 | -1,0 | 4,0 |
| | Percentil 25 | -7,0 | -7,0 | -7,0 | -8,0 | -7,0 | -6,0 | -7,0 | -6,5 |
| | Mediana | -6,0 | -5,0 | -6,0 | -6,0 | -6,0 | -6,0 | -6,0 | -3,5 |
| | Percentil 75 | -1,5 | -1,5 | -2,0 | -1,0 | -1,5 | -3,0 | -5,0 | 0,5 |

T54c.- TIEMPO HASTA NO DOLOR / DOLOR LEVE según GRUPO y CAUSA

| | | Total | | | | | | | |
|---|---|---|---|---|---|---|---|---|---|
| | | CAUSA | | | | | | | |
| | | Total | | Caries | | Obturación | | Resto | |
| | | N | % | N | % | N | % | N | % |
| TIEMPO HASTA NO DOLOR DEFINITIVO | Total | 80 | 100,0 % | 40 | 100,0 % | 23 | 100,0 % | 17 | 100,0 % |
| | Pre | 2 | 2,5 % | 2 | 5,0 % | 0 | 0,0 % | 0 | 0,0 % |
| | 6h | 20 | 25,0 % | 10 | 25,0 % | 4 | 17,4 % | 6 | 35,3 % |
| | 24h | 7 | 8,8 % | 2 | 5,0 % | 3 | 13,0 % | 2 | 11,8 % |
| | 3d | 12 | 15,0 % | 8 | 20,0 % | 1 | 4,3 % | 3 | 17,6 % |
| | No se alcanza | 39 | 48,8 % | 18 | 45,0 % | 15 | 65,2 % | 6 | 35,3 % |

| | | | | | | | | | |
|---|---|---|---|---|---|---|---|---|---|
| TIEMPO HASTA DOLOR LEVE DEFINITIVO | Total | 80 | 100,0 % | 40 | 100,0 % | 23 | 100,0 % | 17 | 100,0 % |
| | Pre | 4 | 5,0 % | 3 | 7,5 % | 0 | 0,0 % | 1 | 5,9 % |
| | 6h | 53 | 66,3 % | 29 | 72,5 % | 13 | 56,5 % | 11 | 64,7 % |
| | 24h | 7 | 8,8 % | 1 | 2,5 % | 4 | 17,4 % | 2 | 11,8 % |
| | 3d | 7 | 8,8 % | 5 | 12,5 % | 2 | 8,7 % | 0 | 0,0 % |
| | No se alcanza | 9 | 11,3 % | 2 | 5,0 % | 4 | 17,4 % | 3 | 17,6 % |

T55- EVOLUCIÓN DOLOR (VAS) según GRUPO y ANESTESIA COMPLEMENTARIA

| | | GRUPO | | | | | | | |
|---|---|---|---|---|---|---|---|---|---|
| | | Total | | | PEC | | | POT | |
| | | ANESTESIA COMPLEMENTARIA | | | ANESTESIA COMPLEMENTARIA | | | ANESTESIA COMPLEMENT | |
| | | Total | No | Sí | Total | No | Sí | Total | No |
| DOLOR pre | N | 80 | 32 | 48 | 40 | 16 | 24 | 40 | 16 |
| | Media | 5,8 | 5,6 | 5,9 | 6,0 | 5,9 | 6,0 | 5,6 | 5,3 |
| | Desviación típica | 2,8 | 2,9 | 2,8 | 2,6 | 2,8 | 2,5 | 3,1 | 3,0 |
| | Mínimo | 0,0 | 0,0 | 0,0 | 0,0 | 0,0 | 1,0 | 0,0 | 0,0 |
| | Máximo | 10,0 | 9,0 | 10,0 | 10,0 | 9,0 | 10,0 | 10,0 | 9,0 |
| | Percentil 25 | 4,0 | 4,0 | 4,0 | 4,0 | 5,0 | 4,0 | 4,0 | 3,0 |
| | Mediana | 6,5 | 6,5 | 6,5 | 7,0 | 7,0 | 6,0 | 6,0 | 5,5 |
| | Percentil 75 | 8,0 | 8,0 | 8,0 | 8,0 | 8,0 | 8,0 | 8,0 | 8,0 |
| DOLOR 6h | N | 80 | 32 | 48 | 40 | 16 | 24 | 40 | 16 |
| | Media | 2,1 | 2,5 | 1,9 | 2,2 | 2,8 | 1,8 | 2,0 | 2,2 |
| | Desviación típica | 2,4 | 2,8 | 2,2 | 2,6 | 2,6 | 2,6 | 2,3 | 3,0 |
| | Mínimo | 0,0 | 0,0 | 0,0 | 0,0 | 0,0 | 0,0 | 0,0 | 0,0 |
| | Máximo | 9,0 | 9,0 | 9,0 | 9,0 | 8,0 | 9,0 | 9,0 | 9,0 |
| | Percentil 25 | 0,0 | 0,0 | 0,0 | 0,0 | 1,0 | 0,0 | 0,0 | 0,0 |
| | Mediana | 2,0 | 2,0 | 1,0 | 2,0 | 2,0 | 0,0 | 1,0 | 0,5 |
| | Percentil 75 | 3,0 | 4,0 | 3,0 | 3,5 | 4,0 | 3,0 | 3,0 | 4,0 |
| DOLOR 24h | N | 80 | 32 | 48 | 40 | 16 | 24 | 40 | 16 |
| | Media | 1,5 | 1,3 | 1,6 | 1,8 | 1,3 | 2,0 | 1,3 | 1,3 |
| | Desviación típica | 2,1 | 1,8 | 2,3 | 2,3 | 1,4 | 2,7 | 2,0 | 2,2 |
| | Mínimo | 0,0 | 0,0 | 0,0 | 0,0 | 0,0 | 0,0 | 0,0 | 0,0 |
| | Máximo | 9,0 | 7,0 | 9,0 | 9,0 | 4,0 | 9,0 | 7,0 | 7,0 |

| | | | | | | | | | |
|---|---|---|---|---|---|---|---|---|---|
| | Percentil 25 | 0,0 | 0,0 | 0,0 | 0,0 | 0,0 | 0,0 | 0,0 | 0,0 |
| | Mediana | 1,0 | 0,5 | 1,0 | 1,0 | 1,0 | 1,0 | 0,0 | 0,0 |
| | Percentil 75 | 2,0 | 2,0 | 2,0 | 2,5 | 2,5 | 3,0 | 1,5 | 1,5 |
| DOLOR 3d | N | 80 | 32 | 48 | 40 | 16 | 24 | 40 | 16 |
| | Media | 1,3 | 1,2 | 1,4 | 1,3 | 1,2 | 1,3 | 1,3 | 1,2 |
| | Desviación típica | 2,0 | 2,1 | 2,0 | 2,1 | 1,6 | 2,4 | 2,0 | 2,5 |
| | Mínimo | 0,0 | 0,0 | 0,0 | 0,0 | 0,0 | 0,0 | 0,0 | 0,0 |
| | Máximo | 10,0 | 10,0 | 10,0 | 10,0 | 6,0 | 10,0 | 10,0 | 10,0 |
| | Percentil 25 | 0,0 | 0,0 | 0,0 | 0,0 | 0,0 | 0,0 | 0,0 | 0,0 |
| | Mediana | 0,0 | 0,0 | 0,0 | 0,0 | 0,5 | 0,0 | 0,5 | 0,0 |
| | Percentil 75 | 2,0 | 2,0 | 2,5 | 2,0 | 2,0 | 2,0 | 2,0 | 1,5 |

T55b- REDUCCIÓN DOLOR (VAS) 3d - PRE según GRUPO y ANESTESIA COMPLEMENTARIA

| | | GRUPO | | | | | | | |
|---|---|---|---|---|---|---|---|---|---|
| | | Total | | | PEC | | | POT | |
| | | ANESTESIA COMPLEMENTARIA | | | ANESTESIA COMPLEMENTARIA | | | ANESTESIA COMPLEMENT/ | |
| | | Total | No | Sí | Total | No | Sí | Total | No |
| DIF.DOLOR.3d_PRE | N | 80 | 32 | 48 | 40 | 16 | 24 | 40 | 16 |
| | Media | -4,5 | -4,4 | -4,5 | -4,7 | -4,8 | -4,7 | -4,3 | -4,1 |
| | Desviación típica | 3,5 | 3,6 | 3,4 | 3,2 | 2,9 | 3,4 | 3,8 | 4,3 |
| | Mínimo | -9,0 | -9,0 | -9,0 | -9,0 | -8,0 | -9,0 | -9,0 | -9,0 |
| | Máximo | 6,0 | 6,0 | 4,0 | 4,0 | 0,0 | 4,0 | 6,0 | 6,0 |
| | Percentil 25 | -7,0 | -7,0 | -7,0 | -7,0 | -7,0 | -7,0 | -7,0 | -7,5 |
| | Mediana | -6,0 | -5,5 | -6,0 | -6,0 | -5,5 | -6,0 | -5,0 | -5,5 |
| | Percentil 75 | -1,5 | -2,0 | -1,0 | -1,5 | -2,0 | -1,5 | -1,5 | -2,0 |

T55c.- TIEMPO HASTA NO DOLOR / DOLOR LEVE según GRUPO y ANESTESIA COMPLEMENTARIA

| | | | | |
|---|---|---|---|---|
| | Total | | | |
| | ANESTESIA COMPLEMENTARIA | | | |
| | Total | No | Sí | Total |

| | | N | % | N | % | N | % | N | % |
|---|---|---|---|---|---|---|---|---|---|
| TIEMPO HASTA NO DOLOR DEFINITIVO | Total | 80 | 100,0 % | 32 | 100,0 % | 48 | 100,0 % | 40 | 100,0 % |
| | Pre | 2 | 2,5 % | 1 | 3,1 % | 1 | 2,1 % | 1 | 2,5 % |
| | 6h | 20 | 25,0 % | 10 | 31,3 % | 10 | 20,8 % | 10 | 25,0 % |
| | 24h | 7 | 8,8 % | 2 | 6,3 % | 5 | 10,4 % | 2 | 5,0 % |
| | 3d | 12 | 15,0 % | 4 | 12,5 % | 8 | 16,7 % | 9 | 22,5 % |
| | No se alcanza | 39 | 48,8 % | 15 | 46,9 % | 24 | 50,0 % | 18 | 45,0 % |
| TIEMPO HASTA DOLOR LEVE DEFINITIVO | Total | 80 | 100,0 % | 32 | 100,0 % | 48 | 100,0 % | 40 | 100,0 % |
| | Pre | 4 | 5,0 % | 1 | 3,1 % | 3 | 6,3 % | 2 | 5,0 % |
| | 6h | 53 | 66,3 % | 21 | 65,6 % | 32 | 66,7 % | 24 | 60,0 % |
| | 24h | 7 | 8,8 % | 5 | 15,6 % | 2 | 4,2 % | 5 | 12,5 % |
| | 3d | 7 | 8,8 % | 3 | 9,4 % | 4 | 8,3 % | 4 | 10,0 % |
| | No se alcanza | 9 | 11,3 % | 2 | 6,3 % | 7 | 14,6 % | 5 | 12,5 % |

T56- EVALUACIÓN SATISFACCIÓN GENERAL TRATAMIENTO (0-nada satisfecho a 10-muy satisfecho) según GRUPO y ANESTESIA COMPLEMENTARIA

| | | GRUPO | | | | | | | |
|---|---|---|---|---|---|---|---|---|---|
| | | Total | | | PEC | | | POT | |
| | | ANESTESIA COMPLEMENTARIA | | | ANESTESIA COMPLEMENTARIA | | | ANESTESIA COMPLEMENT | |
| | | Total | No | Sí | Total | No | Sí | Total | No |
| SATISFACCION | N | 80 | 32 | 48 | 40 | 16 | 24 | 40 | 16 |
| | Media | 9,1 | 9,4 | 8,9 | 9,2 | 9,3 | 9,1 | 9,1 | 9,6 |
| | Desviación típica | 1,8 | 1,2 | 2,1 | 1,7 | 1,4 | 1,9 | 2,0 | 1,1 |
| | Mínimo | 1,0 | 5,0 | 1,0 | 1,0 | 5,0 | 1,0 | 1,0 | 6,0 |
| | Máximo | 10,0 | 10,0 | 10,0 | 10,0 | 10,0 | 10,0 | 10,0 | 10,0 |
| | Percentil 25 | 9,0 | 9,5 | 8,5 | 9,0 | 9,0 | 9,0 | 9,0 | 10,0 |
| | Mediana | 10,0 | 10,0 | 10,0 | 10,0 | 10,0 | 10,0 | 10,0 | 10,0 |
| | Percentil 75 | 10,0 | 10,0 | 10,0 | 10,0 | 10,0 | 10,0 | 10,0 | 10,0 |

T57.- INFLAMACIÓN ENCÍA POST-OPERATORIA según GRUPO E INTRALIGAMENTOSA

| | | |
|---|---|---|
| | Total | |

| | | INTRALIGAMENTOSA | | | | | | | |
|---|---|---|---|---|---|---|---|---|---|
| | | Total | | No | | Sí | | Total | |
| | | N | % | N | % | N | % | N | % |
| INFLAMACION ENCIA | Total | 80 | 100,0 % | 43 | 100,0 % | 37 | 100,0 % | 40 | 100,0 % |
| | No | 76 | 95,0 % | 43 | 100,0 % | 33 | 89,2 % | 38 | 95,0 % |
| | Sí | 4 | 5,0 % | 0 | 0,0 % | 4 | 10,8 % | 2 | 5,0 % |

T58- EVALUACIÓN DURACIÓN TRATAMIENTO (0-muy corto a 10-demasiado largo) según GRUPO y RECONSTRUCCIÓN PRE-ENDODONCIA

| | | GRUPO | | | | | | | |
|---|---|---|---|---|---|---|---|---|---|
| | | Total | | | PEC | | | POT | |
| | | RECO PREENDO | | | RECO PREENDO | | | RECO PREENDO | |
| | | Total | No | Sí | Total | No | Sí | Total | No |
| DURACION | N | 80 | 49 | 31 | 40 | 23 | 17 | 40 | 26 |
| | Media | 2,6 | 2,5 | 2,6 | 3,1 | 3,1 | 3,1 | 2,0 | 2,0 |
| | Desviación típica | 1,8 | 1,8 | 1,9 | 1,9 | 2,1 | 1,7 | 1,6 | 1,3 |
| | Mínimo | 0,0 | 0,0 | 0,0 | 0,0 | 0,0 | 0,0 | 0,0 | 0,0 |
| | Máximo | 10,0 | 10,0 | 6,0 | 10,0 | 10,0 | 6,0 | 5,0 | 4,0 |
| | Percentil 25 | 1,0 | 2,0 | 1,0 | 2,0 | 2,0 | 2,0 | 0,0 | 1,0 |
| | Mediana | 3,0 | 3,0 | 3,0 | 3,0 | 3,0 | 3,0 | 3,0 | 3,0 |
| | Percentil 75 | 3,0 | 3,0 | 5,0 | 3,5 | 3,0 | 5,0 | 3,0 | 3,0 |

| | | | | | | | | | |
|---|---|---|---|---|---|---|---|---|---|
| | | | | | | | | | |
| | | | | | | | | | |
| | | | | | | | | | |
| | | | | | | | | | |
| | | | | | | | | | |
| | | | | | | | | | |
| | | | | | | | | | |
| | | | | | | | | | |
| | | | | | | | | | |
| | | | | | | | | | |
| | | | | | | | | | |
| | | | | | | | | | |
| | | | | | | | | | |
| | | | | | | | | | |
| | | | | | | | | | |
| | | | | | | | | | |
| | | | | | | | | | |
| | | | | | | | | | |
| | | | | | | | | | |
| | | | | | | | | | |
| | | | | | | | | | |
| | | | | | | | | | |
| | | | | | | | | | |
| | | | | | | | | | |
| | | | | | | | | | |
| | | | | | | | | | |
| | | | | | | | | | |
| | | | | | | | | | |
| | | | | | | | | | |
| | | | | | | | | | |
| | | | | | | | | | |

| | | | | | | | | | |
|---|---|---|---|---|---|---|---|---|---|
| | | | | | | | | | |
| | | | | | | | | | |
| | | | | | | | | | |
| | | | | | | | | | |
| | | | | | | | | | |
| | | | | | | | | | |
| | | | | | | | | | |
| | | | | | | | | | |
| | | | | | | | | | |
| | | | | | | | | | |
| | | | | | | | | | |
| | | | | | | | | | |
| | | | | | | | | | |
| | | | | | | | | | |
| | | | | | | | | | |
| | | | | | | | | | |
| | | | | | | | | | |
| | | | | | | | | | |
| | | | | | | | | | |
| | | | | | | | | | |
| | | | | | | | | | |
| | | | | | | | | | |
| | | | | | | | | | |
| | | | | | | | | | |
| | | | | | | | | | |
| | | | | | | | | | |
| | | | | | | | | | |
| | | | | | | | | | |
| | | | | | | | | | |
| | | | | | | | | | |

| | | | | | | | | | |
|---|---|---|---|---|---|---|---|---|---|
| | | | | | | | | | |
| | | | | | | | | | |
| | | | | | | | | | |
| | | | | | | | | | |
| | | | | | | | | | |
| | | | | | | | | | |
| | | | | | | | | | |
| | | | | | | | | | |
| | | | | | | | | | |
| | | | | | | | | | |
| | | | | | | | | | |
| | | | | | | | | | |
| | | | | | | | | | |
| | | | | | | | | | |
| | | | | | | | | | |
| | | | | | | | | | |
| | | | | | | | | | |
| | | | | | | | | | |
| | | | | | | | | | |
| | | | | | | | | | |
| | | | | | | | | | |
| | | | | | | | | | |
| | | | | | | | | | |
| | | | | | | | | | |
| | | | | | | | | | |
| | | | | | | | | | |
| | | | | | | | | | |
| | | | | | | | | | |
| | | | | | | | | | |
| | | | | | | | | | |

| | | | | | | | | | |
|---|---|---|---|---|---|---|---|---|---|
| | | | | | | | | | |
| | | | | | | | | | |
| | | | | | | | | | |
| | | | | | | | | | |
| | | | | | | | | | |
| | | | | | | | | | |
| | | | | | | | | | |
| | | | | | | | | | |
| | | | | | | | | | |
| | | | | | | | | | |
| | | | | | | | | | |
| | | | | | | | | | |
| | | | | | | | | | |
| | | | | | | | | | |
| | | | | | | | | | |
| | | | | | | | | | |
| | | | | | | | | | |
| | | | | | | | | | |
| | | | | | | | | | |
| | | | | | | | | | |
| | | | | | | | | | |
| | | | | | | | | | |
| | | | | | | | | | |
| | | | | | | | | | |
| | | | | | | | | | |
| | | | | | | | | | |
| | | | | | | | | | |
| | | | | | | | | | |
| | | | | | | | | | |
| | | | | | | | | | |

| | | | | | | | | | |
|---|---|---|---|---|---|---|---|---|---|
| | | | | | | | | | |
| | | | | | | | | | |
| | | | | | | | | | |
| | | | | | | | | | |
| | | | | | | | | | |
| | | | | | | | | | |
| | | | | | | | | | |
| | | | | | | | | | |
| | | | | | | | | | |
| | | | | | | | | | |
| | | | | | | | | | |
| | | | | | | | | | |
| | | | | | | | | | |
| | | | | | | | | | |
| | | | | | | | | | |
| | | | | | | | | | |
| | | | | | | | | | |
| | | | | | | | | | |
| | | | | | | | | | |
| | | | | | | | | | |
| | | | | | | | | | |
| | | | | | | | | | |
| | | | | | | | | | |
| | | | | | | | | | |
| | | | | | | | | | |
| | | | | | | | | | |
| | | | | | | | | | |
| | | | | | | | | | |
| | | | | | | | | | |

| | | | | | | | | | |
|---|---|---|---|---|---|---|---|---|---|
| | | | | | | | | | |
| | | | | | | | | | |
| | | | | | | | | | |
| | | | | | | | | | |
| | | | | | | | | | |
| | | | | | | | | | |
| | | | | | | | | | |
| | | | | | | | | | |
| | | | | | | | | | |
| | | | | | | | | | |
| | | | | | | | | | |
| | | | | | | | | | |
| | | | | | | | | | |
| | | | | | | | | | |
| | | | | | | | | | |
| | | | | | | | | | |
| | | | | | | | | | |
| | | | | | | | | | |
| | | | | | | | | | |
| | | | | | | | | | |
| | | | | | | | | | |
| | | | | | | | | | |
| | | | | | | | | | |
| | | | | | | | | | |
| | | | | | | | | | |
| | | | | | | | | | |
| | | | | | | | | | |
| | | | | | | | | | |
| | | | | | | | | | |
| | | | | | | | | | |
| | | | | | | | | | |

| | | | | | | | | | |
|---|---|---|---|---|---|---|---|---|---|
| | | | | | | | | | |
| | | | | | | | | | |
| | | | | | | | | | |
| | | | | | | | | | |
| | | | | | | | | | |
| | | | | | | | | | |
| | | | | | | | | | |
| | | | | | | | | | |
| | | | | | | | | | |
| | | | | | | | | | |
| | | | | | | | | | |
| | | | | | | | | | |
| | | | | | | | | | |
| | | | | | | | | | |
| | | | | | | | | | |
| | | | | | | | | | |
| | | | | | | | | | |
| | | | | | | | | | |
| | | | | | | | | | |
| | | | | | | | | | |
| | | | | | | | | | |
| | | | | | | | | | |
| | | | | | | | | | |
| | | | | | | | | | |
| | | | | | | | | | |
| | | | | | | | | | |
| | | | | | | | | | |
| | | | | | | | | | |
| | | | | | | | | | |
| | | | | | | | | | |
| | | | | | | | | | |

| | | | | | | | | | |
|---|---|---|---|---|---|---|---|---|---|
| | | | | | | | | | |
| | | | | | | | | | |
| | | | | | | | | | |
| | | | | | | | | | |
| | | | | | | | | | |
| | | | | | | | | | |
| | | | | | | | | | |
| | | | | | | | | | |
| | | | | | | | | | |
| | | | | | | | | | |
| | | | | | | | | | |
| | | | | | | | | | |
| | | | | | | | | | |
| | | | | | | | | | |
| | | | | | | | | | |
| | | | | | | | | | |
| | | | | | | | | | |
| | | | | | | | | | |
| | | | | | | | | | |
| | | | | | | | | | |
| | | | | | | | | | |
| | | | | | | | | | |
| | | | | | | | | | |
| | | | | | | | | | |
| | | | | | | | | | |
| | | | | | | | | | |
| | | | | | | | | | |
| | | | | | | | | | |
| | | | | | | | | | |
| | | | | | | | | | |

| Sí |
|---|
| 20 |
| 5,5 |
| 3,4 |
| 0,0 |
| 10,0 |
| 2,5 |
| 6,5 |
| 8,0 |
| 20 |
| 2,0 |
| 2,1 |
| 0,0 |
| 9,0 |
| 0,5 |
| 1,5 |
| 3,0 |
| 20 |
| 1,3 |
| 2,1 |
| 0,0 |
| 7,0 |
| 0,0 |

| | | | | | | | | | |
|---|---|---|---|---|---|---|---|---|---|
| 0,0 | | | | | | | | | |
| 1,0 | | | | | | | | | |
| 20 | | | | | | | | | |
| 1,3 | | | | | | | | | |
| 2,5 | | | | | | | | | |
| 0,0 | | | | | | | | | |
| 10,0 | | | | | | | | | |
| 0,0 | | | | | | | | | |
| 0,0 | | | | | | | | | |
| 1,5 | | | | | | | | | |
| | | | | | | | | | |
| | | | | | | | | | |
| | | | | | | | | | |
| | | | | | | | | | |
| | | | | | | | | | |
| Sí | | | | | | | | | |
| 20 | | | | | | | | | |
| -4,2 | | | | | | | | | |
| 4,6 | | | | | | | | | |
| -9,0 | | | | | | | | | |
| 6,0 | | | | | | | | | |
| -8,0 | | | | | | | | | |
| -6,0 | | | | | | | | | |
| 0,0 | | | | | | | | | |
| | | | | | | | | | |

| GRUPO | | | | | | | | | |
|---|---|---|---|---|---|---|---|---|---|
| PEC | | | | POT | | | | | |
| AINES PRE | | | | AINES PRE | | | | | |
| No | | Sí | | Total | | No | | Sí | |
| N | % | N | % | N | % | N | % | N | % |

| | | | | | | | | | |
|---|---|---|---|---|---|---|---|---|---|
| 23 | 100,0 % | 17 | 100,0 % | 40 | 100,0 % | 20 | 100,0 % | 20 | 100,0 % |
| 1 | 4,3 % | 0 | 0,0 % | 1 | 2,5 % | 0 | 0,0 % | 1 | 5,0 % |
| 6 | 26,1 % | 4 | 23,5 % | 10 | 25,0 % | 6 | 30,0 % | 4 | 20,0 % |
| 2 | 8,7 % | 0 | 0,0 % | 5 | 12,5 % | 1 | 5,0 % | 4 | 20,0 % |
| 6 | 26,1 % | 3 | 17,6 % | 3 | 7,5 % | 0 | 0,0 % | 3 | 15,0 % |
| 8 | 34,8 % | 10 | 58,8 % | 21 | 52,5 % | 13 | 65,0 % | 8 | 40,0 % |
| 23 | 100,0 % | 17 | 100,0 % | 40 | 100,0 % | 20 | 100,0 % | 20 | 100,0 % |
| 2 | 8,7 % | 0 | 0,0 % | 2 | 5,0 % | 0 | 0,0 % | 2 | 10,0 % |
| 15 | 65,2 % | 9 | 52,9 % | 29 | 72,5 % | 15 | 75,0 % | 14 | 70,0 % |
| 2 | 8,7 % | 3 | 17,6 % | 2 | 5,0 % | 1 | 5,0 % | 1 | 5,0 % |
| 2 | 8,7 % | 2 | 11,8 % | 3 | 7,5 % | 3 | 15,0 % | 0 | 0,0 % |
| 2 | 8,7 % | 3 | 17,6 % | 4 | 10,0 % | 1 | 5,0 % | 3 | 15,0 % |

| | | | | | | | | | |
|---|---|---|---|---|---|---|---|---|---|
| Sí | | | | | | | | | |
| 20 | | | | | | | | | |
| 8,7 | | | | | | | | | |
| 2,5 | | | | | | | | | |
| 1,0 | | | | | | | | | |
| 10,0 | | | | | | | | | |
| 8,5 | | | | | | | | | |
| 10,0 | | | | | | | | | |
| 10,0 | | | | | | | | | |

| Sí | | | | | | | | | |
|---|---|---|---|---|---|---|---|---|---|
| 13 | | | | | | | | | |
| 6,2 | | | | | | | | | |
| 3,7 | | | | | | | | | |
| 0,0 | | | | | | | | | |
| 10,0 | | | | | | | | | |
| 5,0 | | | | | | | | | |
| 8,0 | | | | | | | | | |
| 9,0 | | | | | | | | | |
| 13 | | | | | | | | | |
| 1,6 | | | | | | | | | |
| 1,9 | | | | | | | | | |
| 0,0 | | | | | | | | | |
| 6,0 | | | | | | | | | |
| 0,0 | | | | | | | | | |
| 1,0 | | | | | | | | | |
| 2,0 | | | | | | | | | |
| 13 | | | | | | | | | |
| 1,2 | | | | | | | | | |
| 1,6 | | | | | | | | | |
| 0,0 | | | | | | | | | |
| 5,0 | | | | | | | | | |
| 0,0 | | | | | | | | | |
| 1,0 | | | | | | | | | |
| 1,0 | | | | | | | | | |
| 13 | | | | | | | | | |
| 1,0 | | | | | | | | | |
| 1,4 | | | | | | | | | |
| 0,0 | | | | | | | | | |
| 4,0 | | | | | | | | | |
| 0,0 | | | | | | | | | |
| 0,0 | | | | | | | | | |

| | | | | | | | | | |
|---|---|---|---|---|---|---|---|---|---|
| 2,0 | | | | | | | | | |
| | | | | | | | | | |
| | | | | | | | | | |
| | | | | | | | | | |
| | | | | | | | | | |
| | | | | | | | | | |
| Sí | | | | | | | | | |
| 13 | | | | | | | | | |
| -5,2 | | | | | | | | | |
| 4,1 | | | | | | | | | |
| -9,0 | | | | | | | | | |
| 4,0 | | | | | | | | | |
| -8,0 | | | | | | | | | |
| -7,0 | | | | | | | | | |
| -3,0 | | | | | | | | | |
| | | | | | | | | | |

| GRUPO | | | | | | | | | |
|---|---|---|---|---|---|---|---|---|---|
| PEC | | | | POT | | | | | |
| PAA | | | | PAA | | | | | |
| No | | Sí | | Total | | No | | Sí | |
| N | % | N | % | N | % | N | % | N | % |
| 20 | 100,0 % | 20 | 100,0 % | 40 | 100,0 % | 27 | 100,0 % | 13 | 100,0 % |
| 1 | 5,0 % | 0 | 0,0 % | 1 | 2,5 % | 0 | 0,0 % | 1 | 7,7 % |
| 6 | 30,0 % | 4 | 20,0 % | 10 | 25,0 % | 8 | 29,6 % | 2 | 15,4 % |
| 1 | 5,0 % | 1 | 5,0 % | 5 | 12,5 % | 3 | 11,1 % | 2 | 15,4 % |
| 4 | 20,0 % | 5 | 25,0 % | 3 | 7,5 % | 1 | 3,7 % | 2 | 15,4 % |
| 8 | 40,0 % | 10 | 50,0 % | 21 | 52,5 % | 15 | 55,6 % | 6 | 46,2 % |
| 20 | 100,0 % | 20 | 100,0 % | 40 | 100,0 % | 27 | 100,0 % | 13 | 100,0 % |
| 2 | 10,0 % | 0 | 0,0 % | 2 | 5,0 % | 0 | 0,0 % | 2 | 15,4 % |
| 13 | 65,0 % | 11 | 55,0 % | 29 | 72,5 % | 20 | 74,1 % | 9 | 69,2 % |

| | | | | | | | | | |
|---|---|---|---|---|---|---|---|---|---|
| 1 | 5,0 % | 4 | 20,0 % | 2 | 5,0 % | 2 | 7,4 % | 0 | 0,0 % |
| 2 | 10,0 % | 2 | 10,0 % | 3 | 7,5 % | 2 | 7,4 % | 1 | 7,7 % |
| 2 | 10,0 % | 3 | 15,0 % | 4 | 10,0 % | 3 | 11,1 % | 1 | 7,7 % |

| Sí |
|---|
| 13 |
| 9,0 |
| 1,4 |
| 6,0 |
| 10,0 |
| 8,0 |
| 10,0 |
| 10,0 |

| JPO | | | | | | | | |
|---|---|---|---|---|---|---|---|---|
| EC | | | POT | | | | | |
| AA | | | PAA | | | | | |
| o | Sí | | Total | | No | | Sí | |
| % | N | % | N | % | N | % | N | % |
| 100,0 % | 20 | 100,0 % | 40 | 100,0 % | 27 | 100,0 % | 13 | 100,0 % |
| 95,0 % | 17 | 85,0 % | 32 | 80,0 % | 21 | 77,8 % | 11 | 84,6 % |
| 5,0 % | 3 | 15,0 % | 8 | 20,0 % | 6 | 22,2 % | 2 | 15,4 % |

| UPO | | | | | | | | |
|---|---|---|---|---|---|---|---|---|
| EC | | | POT | | | | | |
| A | | | PAA | | | | | |
| o | Sí | | Total | | No | | Sí | |
| % | N | % | N | % | N | % | N | % |
| 100,0 % | 20 | 100,0 % | 40 | 100,0 % | 27 | 100,0 % | 13 | 100,0 % |
| 85,0 % | 11 | 55,0 % | 28 | 70,0 % | 19 | 70,4 % | 9 | 69,2 % |
| 5,0 % | 1 | 5,0 % | 6 | 15,0 % | 3 | 11,1 % | 3 | 23,1 % |
| 10,0 % | 6 | 30,0 % | 2 | 5,0 % | 2 | 7,4 % | 0 | 0,0 % |
| 0,0 % | 2 | 10,0 % | 4 | 10,0 % | 3 | 11,1 % | 1 | 7,7 % |

| RE |
|---|
| Intensa |
| 20 |
| 6,8 |
| 3,0 |

| | | | | | | | | | |
|---|---|---|---|---|---|---|---|---|---|
| 0,0 | | | | | | | | | |
| 10,0 | | | | | | | | | |
| 5,0 | | | | | | | | | |
| 8,0 | | | | | | | | | |
| 9,0 | | | | | | | | | |
| 20 | | | | | | | | | |
| 1,9 | | | | | | | | | |
| 1,7 | | | | | | | | | |
| 0,0 | | | | | | | | | |
| 6,0 | | | | | | | | | |
| 0,0 | | | | | | | | | |
| 2,0 | | | | | | | | | |
| 3,0 | | | | | | | | | |
| 20 | | | | | | | | | |
| 1,2 | | | | | | | | | |
| 1,7 | | | | | | | | | |
| 0,0 | | | | | | | | | |
| 5,0 | | | | | | | | | |
| 0,0 | | | | | | | | | |
| 0,5 | | | | | | | | | |
| 1,5 | | | | | | | | | |
| 20 | | | | | | | | | |
| 1,4 | | | | | | | | | |
| 1,6 | | | | | | | | | |
| 0,0 | | | | | | | | | |
| 5,0 | | | | | | | | | |
| 0,0 | | | | | | | | | |
| 1,0 | | | | | | | | | |
| 2,5 | | | | | | | | | |
| | | | | | | | | | |
| | | | | | | | | | |
| | | | | | | | | | |

| | | | | | | | | | |
|---|---|---|---|---|---|---|---|---|---|
| | | | | | | | | | |
| RE | | | | | | | | | |
| Intensa | | | | | | | | | |
| 20 | | | | | | | | | |
| -5,4 | | | | | | | | | |
| 3,2 | | | | | | | | | |
| -9,0 | | | | | | | | | |
| 1,0 | | | | | | | | | |
| -8,0 | | | | | | | | | |
| -7,0 | | | | | | | | | |
| -3,5 | | | | | | | | | |
| | | | | | | | | | |

| GRUPO | | | | | | | | | |
|---|---|---|---|---|---|---|---|---|---|
| PEC | | | | POT | | | | | |
| MOLESTIA MASTICAR PRE | | | | MOLESTIA MASTICAR PRE | | | | | |
| Hasta moderada | | Intensa | | Total | | Hasta moderada | | Intensa | |
| N | % | N | % | N | % | N | % | N | % |
| 14 | 100,0 % | 26 | 100,0 % | 40 | 100,0 % | 20 | 100,0 % | 20 | 100,0 % |
| 0 | 0,0 % | 1 | 3,8 % | 1 | 2,5 % | 0 | 0,0 % | 1 | 5,0 % |
| 4 | 28,6 % | 6 | 23,1 % | 10 | 25,0 % | 7 | 35,0 % | 3 | 15,0 % |
| 1 | 7,1 % | 1 | 3,8 % | 5 | 12,5 % | 2 | 10,0 % | 3 | 15,0 % |
| 4 | 28,6 % | 5 | 19,2 % | 3 | 7,5 % | 1 | 5,0 % | 2 | 10,0 % |
| 5 | 35,7 % | 13 | 50,0 % | 21 | 52,5 % | 10 | 50,0 % | 11 | 55,0 % |
| 14 | 100,0 % | 26 | 100,0 % | 40 | 100,0 % | 20 | 100,0 % | 20 | 100,0 % |
| 0 | 0,0 % | 2 | 7,7 % | 2 | 5,0 % | 0 | 0,0 % | 2 | 10,0 % |
| 10 | 71,4 % | 14 | 53,8 % | 29 | 72,5 % | 14 | 70,0 % | 15 | 75,0 % |
| 1 | 7,1 % | 4 | 15,4 % | 2 | 5,0 % | 2 | 10,0 % | 0 | 0,0 % |
| 2 | 14,3 % | 2 | 7,7 % | 3 | 7,5 % | 2 | 10,0 % | 1 | 5,0 % |
| 1 | 7,1 % | 4 | 15,4 % | 4 | 10,0 % | 2 | 10,0 % | 2 | 10,0 % |
| | | | | | | | | | |

| |
|---|
| RE |
| Intensa |
| 20 |
| 8,5 |
| 2,6 |
| 1,0 |
| 10,0 |
| 8,0 |
| 10,0 |
| 10,0 |

| UPO | | | | | | | | |
|---|---|---|---|---|---|---|---|---|
| EC | | | POT | | | | | |
| ASTICAR PRE | | | MOLESTIA MASTICAR PRE | | | | | |
| oderada | Intensa | | Total | | Hasta moderada | | Intensa | |
| % | N | % | N | % | N | % | N | % |
| 100,0 % | 26 | 100,0 % | 40 | 100,0 % | 20 | 100,0 % | 20 | 100,0 % |
| 100,0 % | 22 | 84,6 % | 32 | 80,0 % | 13 | 65,0 % | 19 | 95,0 % |
| 0,0 % | 4 | 15,4 % | 8 | 20,0 % | 7 | 35,0 % | 1 | 5,0 % |

| POT | | |
|---|---|---|
| EXPERIENCIA | | |
| No | Sí | NS |
| 9 | 26 | 5 |
| 2,6 | 3,1 | 5,6 |
| 3,1 | 3,6 | 3,5 |
| 0,0 | 0,0 | 0,0 |
| 7,0 | 10,0 | 9,0 |
| 0,0 | 0,0 | 5,0 |
| 0,0 | 0,5 | 6,0 |
| 6,0 | 6,0 | 8,0 |

| POT | | | |
|---|---|---|---|
| | EXPERIENCIA | | |
| Total | No | Sí | NS |
| 40 | 9 | 26 | 5 |
| 1,6 | 2,0 | 1,7 | 0,6 |
| 1,8 | 2,5 | 1,7 | 0,5 |
| 0,0 | 0,0 | 0,0 | 0,0 |

| | | | |
|---|---|---|---|
| 6,0 | 6,0 | 5,0 | 1,0 |
| 0,0 | 0,0 | 0,0 | 0,0 |
| 1,0 | 1,0 | 1,0 | 1,0 |
| 3,0 | 3,0 | 3,0 | 1,0 |

| POT | | | |
|---|---|---|---|
| EXPERIENCIA | | | |
| Total | No | Sí | NS |
| 40 | 9 | 26 | 5 |
| 9,1 | 9,0 | 8,9 | 10,0 |
| 2,0 | 3,0 | 1,8 | 0,0 |
| 1,0 | 1,0 | 3,0 | 10,0 |
| 10,0 | 10,0 | 10,0 | 10,0 |
| 9,0 | 10,0 | 8,0 | 10,0 |
| 10,0 | 10,0 | 10,0 | 10,0 |
| 10,0 | 10,0 | 10,0 | 10,0 |

| | | | | | | | | | |
|---|---|---|---|---|---|---|---|---|---|
| | | | | | | | | | |
| | | | | | | | | | |
| | | | | | | | | | |
| | | | | | | | | | |
| | | | | | | | | | |
| TURA | | | | | | | | | |
| >=15 m | | | | | | | | | |
| 14 | | | | | | | | | |
| 6,0 | | | | | | | | | |
| 2,8 | | | | | | | | | |
| 0,0 | | | | | | | | | |
| 9,0 | | | | | | | | | |
| 5,0 | | | | | | | | | |
| 7,0 | | | | | | | | | |
| 8,0 | | | | | | | | | |
| 14 | | | | | | | | | |
| 2,1 | | | | | | | | | |
| 2,3 | | | | | | | | | |
| 0,0 | | | | | | | | | |
| 9,0 | | | | | | | | | |
| 1,0 | | | | | | | | | |
| 1,5 | | | | | | | | | |
| 3,0 | | | | | | | | | |
| 14 | | | | | | | | | |
| 1,3 | | | | | | | | | |
| 2,2 | | | | | | | | | |
| 0,0 | | | | | | | | | |
| 7,0 | | | | | | | | | |
| 0,0 | | | | | | | | | |
| 0,0 | | | | | | | | | |
| 1,0 | | | | | | | | | |
| 14 | | | | | | | | | |

| |
|---|
| 0,7 |
| 1,3 |
| 0,0 |
| 4,0 |
| 0,0 |
| 0,0 |
| 1,0 |
| |
| |
| |
| |
| TURA |
| >=15 m |
| 14 |
| -5,3 |
| 3,3 |
| -9,0 |
| 1,0 |
| -8,0 |
| -6,0 |
| -4,0 |

| GRUPO | | | | | | | | | |
|---|---|---|---|---|---|---|---|---|---|
| PEC | | | | POT | | | | | |
| TIEMPO ANESTESIA-APERTURA | | | | TIEMPO ANESTESIA-APERTURA | | | | | |
| <15 m | | >=15 m | | Total | | <15 m | | >=15 m | |
| N | % | N | % | N | % | N | % | N | % |
| 16 | 100,0 % | 23 | 100,0 % | 40 | 100,0 % | 26 | 100,0 % | 14 | 100,0 % |
| 1 | 6,3 % | 0 | 0,0 % | 1 | 2,5 % | 1 | 3,8 % | 0 | 0,0 % |
| 7 | 43,8 % | 3 | 13,0 % | 10 | 25,0 % | 7 | 26,9 % | 3 | 21,4 % |

| | | | | | | | | | |
|---|---|---|---|---|---|---|---|---|---|
| 0 | 0,0 % | 2 | 8,7 % | 5 | 12,5 % | 2 | 7,7 % | 3 | 21,4 % |
| 3 | 18,8 % | 5 | 21,7 % | 3 | 7,5 % | 0 | 0,0 % | 3 | 21,4 % |
| 5 | 31,3 % | 13 | 56,5 % | 21 | 52,5 % | 16 | 61,5 % | 5 | 35,7 % |
| 16 | 100,0 % | 23 | 100,0 % | 40 | 100,0 % | 26 | 100,0 % | 14 | 100,0 % |
| 1 | 6,3 % | 1 | 4,3 % | 2 | 5,0 % | 1 | 3,8 % | 1 | 7,1 % |
| 11 | 68,8 % | 12 | 52,2 % | 29 | 72,5 % | 18 | 69,2 % | 11 | 78,6 % |
| 2 | 12,5 % | 3 | 13,0 % | 2 | 5,0 % | 1 | 3,8 % | 1 | 7,1 % |
| 0 | 0,0 % | 4 | 17,4 % | 3 | 7,5 % | 3 | 11,5 % | 0 | 0,0 % |
| 2 | 12,5 % | 3 | 13,0 % | 4 | 10,0 % | 3 | 11,5 % | 1 | 7,1 % |
| | | | | | | | | | |
| | | | | | | | | | |
| | | | | | | | | | |
| | | | | | | | | | |
| TURA | | | | | | | | | |
| >=15 m | | | | | | | | | |
| 14 | | | | | | | | | |
| 1,5 | | | | | | | | | |
| 1,2 | | | | | | | | | |
| 0,0 | | | | | | | | | |
| 3,0 | | | | | | | | | |
| 0,0 | | | | | | | | | |
| 1,5 | | | | | | | | | |
| 3,0 | | | | | | | | | |
| | | | | | | | | | |
| | | | | | | | | | |
| | | | | | | | | | |
| | | | | | | | | | |
| TURA | | | | | | | | | |
| >=15 m | | | | | | | | | |
| 14 | | | | | | | | | |
| 2,1 | | | | | | | | | |

| | | | | | | | | | |
|---|---|---|---|---|---|---|---|---|---|
| 2,2 | | | | | | | | | |
| 0,0 | | | | | | | | | |
| 6,0 | | | | | | | | | |
| 0,0 | | | | | | | | | |
| 1,0 | | | | | | | | | |
| 3,0 | | | | | | | | | |
| | | | | | | | | | |
| | | | | | | | | | |
| | | | | | | | | | |
| | | | | | | | | | |
| TURA | | | | | | | | | |
| >=15 m | | | | | | | | | |
| 14 | | | | | | | | | |
| 8,6 | | | | | | | | | |
| 2,9 | | | | | | | | | |
| 1,0 | | | | | | | | | |
| 10,0 | | | | | | | | | |
| 9,0 | | | | | | | | | |
| 10,0 | | | | | | | | | |
| 10,0 | | | | | | | | | |
| | | | | | | | | | |
| | | | | | | | | | |
| | | | | | | | | | |
| | | | | | | | | | |
| AL | | | | | | | | | |
| >8 m | | | | | | | | | |
| 9 | | | | | | | | | |
| 4,3 | | | | | | | | | |
| 3,8 | | | | | | | | | |
| 0,0 | | | | | | | | | |
| 9,0 | | | | | | | | | |

| | | | | | | | | | |
|---|---|---|---|---|---|---|---|---|---|
| 0,0 | | | | | | | | | |
| 4,0 | | | | | | | | | |
| 8,0 | | | | | | | | | |
| 9 | | | | | | | | | |
| 3,2 | | | | | | | | | |
| 2,5 | | | | | | | | | |
| 0,0 | | | | | | | | | |
| 7,0 | | | | | | | | | |
| 1,0 | | | | | | | | | |
| 3,0 | | | | | | | | | |
| 4,0 | | | | | | | | | |
| 9 | | | | | | | | | |
| 2,6 | | | | | | | | | |
| 2,4 | | | | | | | | | |
| 0,0 | | | | | | | | | |
| 5,0 | | | | | | | | | |
| 1,0 | | | | | | | | | |
| 1,0 | | | | | | | | | |
| 5,0 | | | | | | | | | |
| 9 | | | | | | | | | |
| 2,6 | | | | | | | | | |
| 3,2 | | | | | | | | | |
| 0,0 | | | | | | | | | |
| 10,0 | | | | | | | | | |
| 1,0 | | | | | | | | | |
| 1,0 | | | | | | | | | |
| 4,0 | | | | | | | | | |
| | | | | | | | | | |
| | | | | | | | | | |
| | | | | | | | | | |
| | | | | | | | | | |
| AL | | | | | | | | | |

| >8 m |
| --- |
| 9 |
| -1,8 |
| 5,3 |
| -8,0 |
| 6,0 |
| -7,0 |
| 0,0 |
| 1,0 |

| GRUPO | | | | | | | | | |
| --- | --- | --- | --- | --- | --- | --- | --- | --- | --- |
| PEC | | | | POT | | | | | |
| TIEMPO APERTURA-FINAL | | | | TIEMPO APERTURA-FINAL | | | | | |
| <=8 m | | >8 m | | Total | | <=8 m | | >8 m | |
| N | % | N | % | N | % | N | % | N | % |
| 10 | 100,0 % | 29 | 100,0 % | 40 | 100,0 % | 31 | 100,0 % | 9 | 100,0 % |
| 0 | 0,0 % | 1 | 3,4 % | 1 | 2,5 % | 0 | 0,0 % | 1 | 11,1 % |
| 3 | 30,0 % | 7 | 24,1 % | 10 | 25,0 % | 10 | 32,3 % | 0 | 0,0 % |
| 1 | 10,0 % | 1 | 3,4 % | 5 | 12,5 % | 4 | 12,9 % | 1 | 11,1 % |
| 1 | 10,0 % | 7 | 24,1 % | 3 | 7,5 % | 3 | 9,7 % | 0 | 0,0 % |
| 5 | 50,0 % | 13 | 44,8 % | 21 | 52,5 % | 14 | 45,2 % | 7 | 77,8 % |
| 10 | 100,0 % | 29 | 100,0 % | 40 | 100,0 % | 31 | 100,0 % | 9 | 100,0 % |
| 0 | 0,0 % | 2 | 6,9 % | 2 | 5,0 % | 1 | 3,2 % | 1 | 11,1 % |
| 7 | 70,0 % | 16 | 55,2 % | 29 | 72,5 % | 26 | 83,9 % | 3 | 33,3 % |
| 2 | 20,0 % | 3 | 10,3 % | 2 | 5,0 % | 2 | 6,5 % | 0 | 0,0 % |
| 0 | 0,0 % | 4 | 13,8 % | 3 | 7,5 % | 1 | 3,2 % | 2 | 22,2 % |
| 1 | 10,0 % | 4 | 13,8 % | 4 | 10,0 % | 1 | 3,2 % | 3 | 33,3 % |

| | | | | | | | | | |
|---|---|---|---|---|---|---|---|---|---|
| | | | | | | | | | |
| AL | | | | | | | | | |
| >8 m | | | | | | | | | |
| 9 | | | | | | | | | |
| 2,3 | | | | | | | | | |
| 1,9 | | | | | | | | | |
| 0,0 | | | | | | | | | |
| 5,0 | | | | | | | | | |
| 0,0 | | | | | | | | | |
| 3,0 | | | | | | | | | |
| 3,0 | | | | | | | | | |
| | | | | | | | | | |
| | | | | | | | | | |
| | | | | | | | | | |
| | | | | | | | | | |
| AL | | | | | | | | | |
| >8 m | | | | | | | | | |
| 9 | | | | | | | | | |
| 2,0 | | | | | | | | | |
| 1,7 | | | | | | | | | |
| 0,0 | | | | | | | | | |
| 5,0 | | | | | | | | | |
| 1,0 | | | | | | | | | |
| 2,0 | | | | | | | | | |
| 3,0 | | | | | | | | | |
| | | | | | | | | | |
| | | | | | | | | | |
| | | | | | | | | | |
| | | | | | | | | | |
| AL | | | | | | | | | |
| >8 m | | | | | | | | | |

| | | | | | | | | | |
|---|---|---|---|---|---|---|---|---|---|
| 9 | | | | | | | | | |
| 8,4 | | | | | | | | | |
| 2,3 | | | | | | | | | |
| 3,0 | | | | | | | | | |
| 10,0 | | | | | | | | | |
| 8,0 | | | | | | | | | |
| 9,0 | | | | | | | | | |
| 10,0 | | | | | | | | | |
| | | | | | | | | | |
| | | | | | | | | | |
| | | | | | | | | | |
| | | | | | | | | | |
| | | | | | | | | | |
| **Sí** | | | | | | | | | |
| 13 | | | | | | | | | |
| 4,9 | | | | | | | | | |
| 3,4 | | | | | | | | | |
| 0,0 | | | | | | | | | |
| 10,0 | | | | | | | | | |
| 2,0 | | | | | | | | | |
| 5,0 | | | | | | | | | |
| 8,0 | | | | | | | | | |
| 13 | | | | | | | | | |
| 2,6 | | | | | | | | | |
| 2,9 | | | | | | | | | |
| 0,0 | | | | | | | | | |
| 9,0 | | | | | | | | | |
| 1,0 | | | | | | | | | |
| 1,0 | | | | | | | | | |
| 4,0 | | | | | | | | | |
| 13 | | | | | | | | | |
| 1,7 | | | | | | | | | |

| | | | | | | | | | |
|---|---|---|---|---|---|---|---|---|---|
| 2,4 | | | | | | | | | |
| 0,0 | | | | | | | | | |
| 7,0 | | | | | | | | | |
| 0,0 | | | | | | | | | |
| 1,0 | | | | | | | | | |
| 2,0 | | | | | | | | | |
| 13 | | | | | | | | | |
| 1,6 | | | | | | | | | |
| 2,8 | | | | | | | | | |
| 0,0 | | | | | | | | | |
| 10,0 | | | | | | | | | |
| 0,0 | | | | | | | | | |
| 1,0 | | | | | | | | | |
| 1,0 | | | | | | | | | |
| | | | | | | | | | |
| | | | | | | | | | |
| | | | | | | | | | |
| | | | | | | | | | |
| | | | | | | | | | |
| **Sí** | | | | | | | | | |
| 13 | | | | | | | | | |
| -3,3 | | | | | | | | | |
| 5,0 | | | | | | | | | |
| -9,0 | | | | | | | | | |
| 6,0 | | | | | | | | | |
| -8,0 | | | | | | | | | |
| -5,0 | | | | | | | | | |
| 0,0 | | | | | | | | | |
| | | | | | | | | | |

**GRUPO**

| PEC | | | | POT | | | | | |
|---|---|---|---|---|---|---|---|---|---|
| SANGRADO PROFUSO | | | | SANGRADO PROFUSO | | | | | |
| No | | Sí | | Total | | No | | Sí | |
| N | % | N | % | N | % | N | % | N | % |
| 24 | 100,0 % | 16 | 100,0 % | 40 | 100,0 % | 27 | 100,0 % | 13 | 100,0 % |
| 0 | 0,0 % | 1 | 6,3 % | 1 | 2,5 % | 1 | 3,7 % | 0 | 0,0 % |
| 4 | 16,7 % | 6 | 37,5 % | 10 | 25,0 % | 7 | 25,9 % | 3 | 23,1 % |
| 1 | 4,2 % | 1 | 6,3 % | 5 | 12,5 % | 5 | 18,5 % | 0 | 0,0 % |
| 8 | 33,3 % | 1 | 6,3 % | 3 | 7,5 % | 1 | 3,7 % | 2 | 15,4 % |
| 11 | 45,8 % | 7 | 43,8 % | 21 | 52,5 % | 13 | 48,1 % | 8 | 61,5 % |
| 24 | 100,0 % | 16 | 100,0 % | 40 | 100,0 % | 27 | 100,0 % | 13 | 100,0 % |
| 1 | 4,2 % | 1 | 6,3 % | 2 | 5,0 % | 2 | 7,4 % | 0 | 0,0 % |
| 14 | 58,3 % | 10 | 62,5 % | 29 | 72,5 % | 21 | 77,8 % | 8 | 61,5 % |
| 2 | 8,3 % | 3 | 18,8 % | 2 | 5,0 % | 0 | 0,0 % | 2 | 15,4 % |
| 2 | 8,3 % | 2 | 12,5 % | 3 | 7,5 % | 2 | 7,4 % | 1 | 7,7 % |
| 5 | 20,8 % | 0 | 0,0 % | 4 | 10,0 % | 2 | 7,4 % | 2 | 15,4 % |

| Sí |
|---|
| 13 |
| 9,5 |
| 1,1 |
| 7,0 |
| 10,0 |
| 10,0 |
| 10,0 |
| 10,0 |

| GRUPO | | | | | | | | | | |
|---|---|---|---|---|---|---|---|---|---|---|
| PEC | | | | | | | | | | |
| CAUSA | | | | | | | | | | |
| tal | Caries | | Obturación | | Resto | | Total | | Car | |
| % | N | % | N | % | N | % | N | % | N | |
| 100,0 % | 19 | 100,0 % | 13 | 100,0 % | 8 | 100,0 % | 40 | 100,0 % | 21 | |
| 60,0 % | 12 | 63,2 % | 8 | 61,5 % | 4 | 50,0 % | 27 | 67,5 % | 15 | |
| 40,0 % | 7 | 36,8 % | 5 | 38,5 % | 4 | 50,0 % | 13 | 32,5 % | 6 | |
| | | | | | | | | | | |

| GRUPO | | | | | | | | | | |
|---|---|---|---|---|---|---|---|---|---|---|
| PEC | | | | | | | | | | |
| CAUSA | | | | | | | | | | |
| tal | Caries | | Obturación | | Resto | | Total | | Car | |
| % | N | % | N | % | N | % | N | % | N | |
| 100,0 % | 19 | 100,0 % | 13 | 100,0 % | 8 | 100,0 % | 40 | 100,0 % | 21 | |
| 70,0 % | 14 | 73,7 % | 9 | 69,2 % | 5 | 62,5 % | 31 | 77,5 % | 15 | |
| 30,0 % | 5 | 26,3 % | 4 | 30,8 % | 3 | 37,5 % | 9 | 22,5 % | 6 | |
| | | | | | | | | | | |

| GRUPO | | | | | | | | | | |
|---|---|---|---|---|---|---|---|---|---|---|
| PEC | | | | | | | | | | |
| CAUSA | | | | | | | | | | |
| tal | Caries | | Obturación | | Resto | | Total | | Car | |
| % | N | % | N | % | N | % | N | % | N | |
| 100,0 % | 19 | 100,0 % | 13 | 100,0 % | 8 | 100,0 % | 40 | 100,0 % | 21 | |
| 50,0 % | 11 | 57,9 % | 3 | 23,1 % | 6 | 75,0 % | 27 | 67,5 % | 13 | |
| 50,0 % | 8 | 42,1 % | 10 | 76,9 % | 2 | 25,0 % | 13 | 32,5 % | 8 | |
| | | | | | | | | | | |

| POT | | | |
|---|---|---|---|
| CAUSA | | | |
| Total | Caries | Obturación | Resto |
| 40 | 21 | 10 | 9 |
| 5,6 | 5,1 | 5,5 | 7,0 |
| 3,1 | 3,1 | 2,9 | 3,1 |
| 0,0 | 0,0 | 0,0 | 0,0 |
| 10,0 | 9,0 | 9,0 | 10,0 |
| 4,0 | 3,0 | 4,0 | 6,0 |
| 6,0 | 5,0 | 6,5 | 8,0 |
| 8,0 | 8,0 | 8,0 | 9,0 |
| 40 | 21 | 10 | 9 |
| 2,0 | 2,3 | 1,6 | 1,9 |
| 2,3 | 2,1 | 2,2 | 2,9 |
| 0,0 | 0,0 | 0,0 | 0,0 |
| 9,0 | 7,0 | 7,0 | 9,0 |
| 0,0 | 0,0 | 0,0 | 0,0 |
| 1,0 | 2,0 | 1,0 | 1,0 |
| 3,0 | 3,0 | 2,0 | 3,0 |
| 40 | 21 | 10 | 9 |
| 1,3 | 1,3 | 0,9 | 1,4 |
| 2,0 | 1,8 | 1,7 | 2,7 |
| 0,0 | 0,0 | 0,0 | 0,0 |
| 7,0 | 5,0 | 5,0 | 7,0 |
| 0,0 | 0,0 | 0,0 | 0,0 |
| 0,0 | 1,0 | 0,0 | 0,0 |
| 1,5 | 2,0 | 1,0 | 1,0 |
| 40 | 21 | 10 | 9 |
| 1,3 | 1,0 | 2,4 | 0,8 |
| 2,0 | 1,3 | 3,0 | 1,6 |

| | | | |
|---|---|---|---|
| 0,0 | 0,0 | 0,0 | 0,0 |
| 10,0 | 4,0 | 10,0 | 5,0 |
| 0,0 | 0,0 | 0,0 | 0,0 |
| 0,5 | 1,0 | 2,5 | 0,0 |
| 2,0 | 2,0 | 3,0 | 1,0 |

| POT | | | |
|---|---|---|---|
| CAUSA | | | |
| Total | Caries | Obturación | Resto |
| 40 | 21 | 10 | 9 |
| -4,3 | -4,0 | -3,1 | -6,2 |
| 3,8 | 3,6 | 4,7 | 2,8 |
| -9,0 | -8,0 | -8,0 | -9,0 |
| 6,0 | 4,0 | 6,0 | 0,0 |
| -7,0 | -7,0 | -6,0 | -8,0 |
| -5,0 | -5,0 | -4,5 | -6,0 |
| -1,5 | -1,0 | -1,0 | -5,0 |

| GRUPO | | | | | | | | | |
|---|---|---|---|---|---|---|---|---|---|
| PEC | | | | | | | | | |
| CAUSA | | | | | | | | | |
| Total | | Caries | | Obturación | | Resto | | Total | |
| N | % | N | % | N | % | N | % | N | % |
| 40 | 100,0 % | 19 | 100,0 % | 13 | 100,0 % | 8 | 100,0 % | 40 | 100,0 % |
| 1 | 2,5 % | 1 | 5,3 % | 0 | 0,0 % | 0 | 0,0 % | 1 | 2,5 % |
| 10 | 25,0 % | 6 | 31,6 % | 2 | 15,4 % | 2 | 25,0 % | 10 | 25,0 % |
| 2 | 5,0 % | 0 | 0,0 % | 1 | 7,7 % | 1 | 12,5 % | 5 | 12,5 % |
| 9 | 22,5 % | 6 | 31,6 % | 1 | 7,7 % | 2 | 25,0 % | 3 | 7,5 % |
| 18 | 45,0 % | 6 | 31,6 % | 9 | 69,2 % | 3 | 37,5 % | 21 | 52,5 % |

| | | | | | | | | | |
|---|---|---|---|---|---|---|---|---|---|
| 40 | 100,0 % | 19 | 100,0 % | 13 | 100,0 % | 8 | 100,0 % | 40 | 100,0 % |
| 2 | 5,0 % | 2 | 10,5 % | 0 | 0,0 % | 0 | 0,0 % | 2 | 5,0 % |
| 24 | 60,0 % | 14 | 73,7 % | 5 | 38,5 % | 5 | 62,5 % | 29 | 72,5 % |
| 5 | 12,5 % | 0 | 0,0 % | 4 | 30,8 % | 1 | 12,5 % | 2 | 5,0 % |
| 4 | 10,0 % | 3 | 15,8 % | 1 | 7,7 % | 0 | 0,0 % | 3 | 7,5 % |
| 5 | 12,5 % | 0 | 0,0 % | 3 | 23,1 % | 2 | 25,0 % | 4 | 10,0 % |

| | | | | | | | | | |
|---|---|---|---|---|---|---|---|---|---|
| | | | | | | | | | |
| | | | | | | | | | |
| | | | | | | | | | |
| | | | | | | | | | |
| ARIA | | | | | | | | | |
| Sí | | | | | | | | | |
| 24 | | | | | | | | | |
| 5,8 | | | | | | | | | |
| 3,1 | | | | | | | | | |
| 0,0 | | | | | | | | | |
| 10,0 | | | | | | | | | |
| 4,0 | | | | | | | | | |
| 7,0 | | | | | | | | | |
| 8,0 | | | | | | | | | |
| 24 | | | | | | | | | |
| 1,9 | | | | | | | | | |
| 1,7 | | | | | | | | | |
| 0,0 | | | | | | | | | |
| 7,0 | | | | | | | | | |
| 0,5 | | | | | | | | | |
| 2,0 | | | | | | | | | |
| 3,0 | | | | | | | | | |
| 24 | | | | | | | | | |
| 1,3 | | | | | | | | | |
| 1,9 | | | | | | | | | |
| 0,0 | | | | | | | | | |
| 5,0 | | | | | | | | | |

| | | | | | | | | | |
|---|---|---|---|---|---|---|---|---|---|
| 0,0 | | | | | | | | | |
| 0,0 | | | | | | | | | |
| 1,5 | | | | | | | | | |
| 24 | | | | | | | | | |
| 1,4 | | | | | | | | | |
| 1,6 | | | | | | | | | |
| 0,0 | | | | | | | | | |
| 5,0 | | | | | | | | | |
| 0,0 | | | | | | | | | |
| 1,0 | | | | | | | | | |
| 3,0 | | | | | | | | | |
| | | | | | | | | | |
| | | | | | | | | | |
| | | | | | | | | | |
| | | | | | | | | | |
| ARIA | | | | | | | | | |
| Sí | | | | | | | | | |
| 24 | | | | | | | | | |
| -4,4 | | | | | | | | | |
| 3,5 | | | | | | | | | |
| -9,0 | | | | | | | | | |
| 4,0 | | | | | | | | | |
| -7,0 | | | | | | | | | |
| -5,0 | | | | | | | | | |
| -1,0 | | | | | | | | | |
| | | | | | | | | | |

| GRUPO | | | | |
|---|---|---|---|---|
| PEC | | POT | | |
| ANESTESIA COMPLEMENTARIA | | ANESTESIA COMPLEMENTARIA | | |
| No | Sí | Total | No | Sí |

| N | % | N | % | N | % | N | % | N | % |
|---|---|---|---|---|---|---|---|---|---|
| 16 | 100,0 % | 24 | 100,0 % | 40 | 100,0 % | 16 | 100,0 % | 24 | 100,0 % |
| 1 | 6,3 % | 0 | 0,0 % | 1 | 2,5 % | 0 | 0,0 % | 1 | 4,2 % |
| 3 | 18,8 % | 7 | 29,2 % | 10 | 25,0 % | 7 | 43,8 % | 3 | 12,5 % |
| 1 | 6,3 % | 1 | 4,2 % | 5 | 12,5 % | 1 | 6,3 % | 4 | 16,7 % |
| 3 | 18,8 % | 6 | 25,0 % | 3 | 7,5 % | 1 | 6,3 % | 2 | 8,3 % |
| 8 | 50,0 % | 10 | 41,7 % | 21 | 52,5 % | 7 | 43,8 % | 14 | 58,3 % |
| 16 | 100,0 % | 24 | 100,0 % | 40 | 100,0 % | 16 | 100,0 % | 24 | 100,0 % |
| 1 | 6,3 % | 1 | 4,2 % | 2 | 5,0 % | 0 | 0,0 % | 2 | 8,3 % |
| 10 | 62,5 % | 14 | 58,3 % | 29 | 72,5 % | 11 | 68,8 % | 18 | 75,0 % |
| 3 | 18,8 % | 2 | 8,3 % | 2 | 5,0 % | 2 | 12,5 % | 0 | 0,0 % |
| 1 | 6,3 % | 3 | 12,5 % | 3 | 7,5 % | 2 | 12,5 % | 1 | 4,2 % |
| 1 | 6,3 % | 4 | 16,7 % | 4 | 10,0 % | 1 | 6,3 % | 3 | 12,5 % |

| ARIA |
|---|
| Sí |
| 24 |
| 8,7 |
| 2,4 |
| 1,0 |
| 10,0 |
| 8,0 |
| 10,0 |
| 10,0 |

| GRUPO | |
|---|---|
| PEC | POT |

| INTRALIGAMENTOSA | | | | INTRALIGAMENTOSA | | | | | |
|---|---|---|---|---|---|---|---|---|---|
| No | | Sí | | Total | | No | | Sí | |
| N | % | N | % | N | % | N | % | N | % |
| 23 | 100,0 % | 17 | 100,0 % | 40 | 100,0 % | 20 | 100,0 % | 20 | 100,0 % |
| 23 | 100,0 % | 15 | 88,2 % | 38 | 95,0 % | 20 | 100,0 % | 18 | 90,0 % |
| 0 | 0,0 % | 2 | 11,8 % | 2 | 5,0 % | 0 | 0,0 % | 2 | 10,0 % |

| | | | | | | | | | |
|---|---|---|---|---|---|---|---|---|---|
| | | | | | | | | | |
| | | | | | | | | | |
| | | | | | | | | | |
| | | | | | | | | | |
| | | | | | | | | | |
| Sí | | | | | | | | | |
| 14 | | | | | | | | | |
| 2,1 | | | | | | | | | |
| 2,1 | | | | | | | | | |
| 0,0 | | | | | | | | | |
| 5,0 | | | | | | | | | |
| 0,0 | | | | | | | | | |
| 2,5 | | | | | | | | | |
| 3,0 | | | | | | | | | |

| | | | | | |
|---|---|---|---|---|---|
| | | | | | |
| | | | | | |
| | | | | | |
| | | | | | |
| | | | | | |
| | | | | | |
| | | | | | |
| | | | | | |
| | | | | | |
| | | | | | |
| | | | | | |
| | | | | | |
| | | | | | |
| | | | | | |
| | | | | | |
| | | | | | |
| | | | | | |
| | | | | | |
| | | | | | |
| | | | | | |
| | | | | | |
| | | | | | |
| | | | | | |
| | | | | | |
| | | | | | |
| | | | | | |
| | | | | | |
| | | | | | |
| | | | | | |
| | | | | | |

| | | | | | |
|---|---|---|---|---|---|
| | | | | | |
| | | | | | |
| | | | | | |
| | | | | | |
| | | | | | |
| | | | | | |
| | | | | | |
| | | | | | |
| | | | | | |
| | | | | | |
| | | | | | |
| | | | | | |
| | | | | | |
| | | | | | |
| | | | | | |
| | | | | | |
| | | | | | |
| | | | | | |
| | | | | | |
| | | | | | |
| | | | | | |
| | | | | | |
| | | | | | |
| | | | | | |
| | | | | | |
| | | | | | |
| | | | | | |
| | | | | | |
| | | | | | |
| | | | | | |

| | | | | | |
|---|---|---|---|---|---|
| | | | | | |
| | | | | | |
| | | | | | |
| | | | | | |
| | | | | | |
| | | | | | |
| | | | | | |
| | | | | | |
| | | | | | |
| | | | | | |
| | | | | | |
| | | | | | |
| | | | | | |
| | | | | | |
| | | | | | |
| | | | | | |
| | | | | | |
| | | | | | |
| | | | | | |
| | | | | | |
| | | | | | |
| | | | | | |
| | | | | | |
| | | | | | |
| | | | | | |
| | | | | | |
| | | | | | |
| | | | | | |
| | | | | | |
| | | | | | |

| | | | | | |
|---|---|---|---|---|---|
| | | | | | |
| | | | | | |
| | | | | | |
| | | | | | |
| | | | | | |
| | | | | | |
| | | | | | |
| | | | | | |
| | | | | | |
| | | | | | |
| | | | | | |
| | | | | | |
| | | | | | |
| | | | | | |
| | | | | | |
| | | | | | |
| | | | | | |
| | | | | | |
| | | | | | |
| | | | | | |
| | | | | | |
| | | | | | |
| | | | | | |
| | | | | | |
| | | | | | |
| | | | | | |
| | | | | | |
| | | | | | |
| | | | | | |
| | | | | | |

| | | | | | |
|---|---|---|---|---|---|
| | | | | | |
| | | | | | |
| | | | | | |
| | | | | | |
| | | | | | |
| | | | | | |
| | | | | | |
| | | | | | |
| | | | | | |
| | | | | | |
| | | | | | |
| | | | | | |
| | | | | | |
| | | | | | |
| | | | | | |
| | | | | | |
| | | | | | |
| | | | | | |
| | | | | | |
| | | | | | |
| | | | | | |
| | | | | | |
| | | | | | |
| | | | | | |
| | | | | | |
| | | | | | |
| | | | | | |
| | | | | | |
| | | | | | |
| | | | | | |

| | | | | | |
|---|---|---|---|---|---|
| | | | | | |
| | | | | | |
| | | | | | |
| | | | | | |
| | | | | | |
| | | | | | |
| | | | | | |
| | | | | | |
| | | | | | |
| | | | | | |
| | | | | | |
| | | | | | |
| | | | | | |
| | | | | | |
| | | | | | |
| | | | | | |
| | | | | | |
| | | | | | |
| | | | | | |
| | | | | | |
| | | | | | |
| | | | | | |
| | | | | | |
| | | | | | |
| | | | | | |
| | | | | | |
| | | | | | |
| | | | | | |
| | | | | | |
| | | | | | |

| | | | | | |
|---|---|---|---|---|---|
| | | | | | |
| | | | | | |
| | | | | | |
| | | | | | |
| | | | | | |
| | | | | | |
| | | | | | |
| | | | | | |
| | | | | | |
| | | | | | |
| | | | | | |
| | | | | | |
| | | | | | |
| | | | | | |
| | | | | | |
| | | | | | |
| | | | | | |
| | | | | | |
| | | | | | |
| | | | | | |
| | | | | | |
| | | | | | |
| | | | | | |
| | | | | | |
| | | | | | |
| | | | | | |
| | | | | | |
| | | | | | |
| | | | | | |
| | | | | | |
| | | | | | |

| | | | | | |
|---|---|---|---|---|---|
| | | | | | |
| | | | | | |
| | | | | | |
| | | | | | |
| | | | | | |
| | | | | | |
| | | | | | |
| | | | | | |
| | | | | | |
| | | | | | |
| | | | | | |
| | | | | | |
| | | | | | |
| | | | | | |
| | | | | | |
| | | | | | |
| | | | | | |
| | | | | | |
| | | | | | |
| | | | | | |
| | | | | | |
| | | | | | |
| | | | | | |
| | | | | | |
| | | | | | |
| | | | | | |
| | | | | | |
| | | | | | |
| | | | | | |
| | | | | | |
| | | | | | |

| | | | | | |
|---|---|---|---|---|---|
| | | | | | |
| | | | | | |
| | | | | | |
| | | | | | |
| | | | | | |
| | | | | | |
| | | | | | |
| | | | | | |
| | | | | | |
| | | | | | |
| | | | | | |
| | | | | | |
| | | | | | |
| | | | | | |
| | | | | | |
| | | | | | |
| | | | | | |
| | | | | | |
| | | | | | |
| | | | | | |
| | | | | | |
| | | | | | |
| | | | | | |
| | | | | | |
| | | | | | |
| | | | | | |
| | | | | | |
| | | | | | |
| | | | | | |
| | | | | | |
| | | | | | |

| | | | | | |
|---|---|---|---|---|---|
| | | | | | |
| | | | | | |
| | | | | | |
| | | | | | |
| | | | | | |
| | | | | | |
| | | | | | |
| | | | | | |
| | | | | | |
| | | | | | |
| | | | | | |
| | | | | | |
| | | | | | |
| | | | | | |
| | | | | | |
| | | | | | |
| | | | | | |
| | | | | | |
| | | | | | |
| | | | | | |
| | | | | | |
| | | | | | |
| | | | | | |
| | | | | | |
| | | | | | |
| | | | | | |
| | | | | | |
| | | | | | |
| | | | | | |
| | | | | | |
| | | | | | |

| | | | | | |
|---|---|---|---|---|---|
| | | | | | |
| | | | | | |
| | | | | | |
| | | | | | |
| | | | | | |
| | | | | | |
| | | | | | |
| | | | | | |
| | | | | | |
| | | | | | |
| | | | | | |
| | | | | | |
| | | | | | |
| | | | | | |
| | | | | | |
| | | | | | |
| | | | | | |
| | | | | | |
| | | | | | |
| | | | | | |
| | | | | | |
| | | | | | |
| | | | | | |
| | | | | | |
| | | | | | |
| | | | | | |
| | | | | | |
| | | | | | |
| | | | | | |
| | | | | | |

| | | | | | |
|---|---|---|---|---|---|
| | | | | | |
| | | | | | |
| | | | | | |
| | | | | | |
| | | | | | |
| | | | | | |
| | | | | | |
| | | | | | |
| | | | | | |
| | | | | | |
| | | | | | |
| | | | | | |
| | | | | | |
| | | | | | |
| | | | | | |
| | | | | | |
| | | | | | |
| | | | | | |
| | | | | | |
| | | | | | |
| | | | | | |
| | | | | | |
| | | | | | |
| | | | | | |
| | | | | | |
| | | | | | |
| | | | | | |
| | | | | | |
| | | | | | |
| | | | | | |

| | | | | | |
|---|---|---|---|---|---|
| | | | | | |
| | | | | | |
| | | | | | |
| | | | | | |
| | | | | | |
| | | | | | |
| | | | | | |
| | | | | | |
| | | | | | |
| | | | | | |
| | | | | | |
| | | | | | |
| | | | | | |
| | | | | | |
| | | | | | |
| | | | | | |
| | | | | | |
| | | | | | |
| | | | | | |
| | | | | | |
| | | | | | |
| | | | | | |
| | | | | | |
| | | | | | |
| | | | | | |
| | | | | | |
| | | | | | |
| | | | | | |
| | | | | | |

| | | | | | |
|---|---|---|---|---|---|
| | | | | | |
| | | | | | |
| | | | | | |
| | | | | | |
| | | | | | |
| | | | | | |
| | | | | | |
| | | | | | |
| | | | | | |
| | | | | | |
| | | | | | |
| | | | | | |
| | | | | | |
| | | | | | |
| | | | | | |
| | | | | | |
| | | | | | |
| | | | | | |
| | | | | | |
| | | | | | |
| | | | | | |
| | | | | | |
| | | | | | |
| | | | | | |
| | | | | | |
| | | | | | |
| | | | | | |
| | | | | | |
| | | | | | |
| | | | | | |

| | | | | | |
|---|---|---|---|---|---|
| | | | | | |
| | | | | | |
| | | | | | |
| | | | | | |
| | | | | | |
| | | | | | |
| | | | | | |
| | | | | | |
| | | | | | |
| | | | | | |
| | | | | | |
| | | | | | |
| | | | | | |
| | | | | | |
| | | | | | |
| | | | | | |
| | | | | | |
| | | | | | |
| | | | | | |
| | | | | | |
| | | | | | |
| | | | | | |
| | | | | | |
| | | | | | |
| | | | | | |
| | | | | | |
| | | | | | |
| | | | | | |
| | | | | | |
| | | | | | |
| | | | | | |

| | | | | | |
|---|---|---|---|---|---|
| | | | | | |
| | | | | | |
| | | | | | |
| | | | | | |
| | | | | | |
| | | | | | |
| | | | | | |
| | | | | | |
| | | | | | |
| | | | | | |
| | | | | | |
| | | | | | |
| | | | | | |
| | | | | | |
| | | | | | |
| | | | | | |
| | | | | | |
| | | | | | |
| | | | | | |
| | | | | | |
| | | | | | |
| | | | | | |
| | | | | | |
| | | | | | |
| | | | | | |
| | | | | | |
| | | | | | |
| | | | | | |
| | | | | | |
| | | | | | |

| | | | | | |
|---|---|---|---|---|---|
| | | | | | |
| | | | | | |
| | | | | | |
| | | | | | |
| | | | | | |
| | | | | | |
| | | | | | |
| | | | | | |
| | | | | | |
| | | | | | |
| | | | | | |
| | | | | | |
| | | | | | |
| | | | | | |
| | | | | | |
| | | | | | |
| | | | | | |
| | | | | | |
| | | | | | |
| | | | | | |
| | | | | | |
| | | | | | |
| | | | | | |
| | | | | | |
| | | | | | |
| | | | | | |
| | | | | | |
| | | | | | |
| | | | | | |
| | | | | | |

| | | | | | |
|---|---|---|---|---|---|
| | | | | | |
| | | | | | |
| | | | | | |
| | | | | | |
| | | | | | |
| | | | | | |
| | | | | | |
| | | | | | |
| | | | | | |
| | | | | | |
| | | | | | |
| | | | | | |
| | | | | | |
| | | | | | |
| | | | | | |
| | | | | | |
| | | | | | |
| | | | | | |
| | | | | | |
| | | | | | |
| | | | | | |
| | | | | | |
| | | | | | |
| | | | | | |
| | | | | | |
| | | | | | |
| | | | | | |
| | | | | | |
| | | | | | |
| | | | | | |

| | | | | | |
|---|---|---|---|---|---|
| | | | | | |
| | | | | | |
| | | | | | |
| | | | | | |
| | | | | | |
| | | | | | |
| | | | | | |
| | | | | | |
| | | | | | |
| | | | | | |
| | | | | | |
| | | | | | |
| | | | | | |
| | | | | | |
| | | | | | |
| | | | | | |
| | | | | | |
| | | | | | |
| | | | | | |
| | | | | | |
| | | | | | |
| | | | | | |
| | | | | | |
| | | | | | |
| | | | | | |
| | | | | | |
| | | | | | |
| | | | | | |
| | | | | | |
| | | | | | |
| | | | | | |

| | | | | | |
|---|---|---|---|---|---|
| | | | | | |
| | | | | | |
| | | | | | |
| | | | | | |
| | | | | | |
| | | | | | |
| | | | | | |
| | | | | | |
| | | | | | |
| | | | | | |
| | | | | | |
| | | | | | |
| | | | | | |
| | | | | | |
| | | | | | |
| | | | | | |
| | | | | | |
| | | | | | |
| | | | | | |
| | | | | | |
| | | | | | |
| | | | | | |
| | | | | | |
| | | | | | |
| | | | | | |
| | | | | | |
| | | | | | |
| | | | | | |
| | | | | | |
| | | | | | |

| POT | | | | |
|---|---|---|---|---|
| CAUSA | | | | |
| ies | Obturación | | Resto | |
| % | N | % | N | % |
| 100,0 % | 10 | 100,0 % | 9 | 100,0 % |
| 71,4 % | 8 | 80,0 % | 4 | 44,4 % |
| 28,6 % | 2 | 20,0 % | 5 | 55,6 % |

| POT | | | | |
|---|---|---|---|---|
| CAUSA | | | | |
| ies | Obturación | | Resto | |
| % | N | % | N | % |
| 100,0 % | 10 | 100,0 % | 9 | 100,0 % |
| 71,4 % | 9 | 90,0 % | 7 | 77,8 % |
| 28,6 % | 1 | 10,0 % | 2 | 22,2 % |

| POT | | | | |
|---|---|---|---|---|
| CAUSA | | | | |
| ies | Obturación | | Resto | |
| % | N | % | N | % |
| 100,0 % | 10 | 100,0 % | 9 | 100,0 % |
| 61,9 % | 8 | 80,0 % | 6 | 66,7 % |
| 38,1 % | 2 | 20,0 % | 3 | 33,3 % |

| POT | | | | | |
|---|---|---|---|---|---|
| CAUSA | | | | | |
| Caries | | Obturación | | Resto | |
| N | % | N | % | N | % |
| 21 | 100,0 % | 10 | 100,0 % | 9 | 100,0 % |
| 1 | 4,8 % | 0 | 0,0 % | 0 | 0,0 % |
| 4 | 19,0 % | 2 | 20,0 % | 4 | 44,4 % |
| 2 | 9,5 % | 2 | 20,0 % | 1 | 11,1 % |
| 2 | 9,5 % | 0 | 0,0 % | 1 | 11,1 % |
| 12 | 57,1 % | 6 | 60,0 % | 3 | 33,3 % |

| | | | | | |
|---|---|---|---|---|---|
| 21 | 100,0 % | 10 | 100,0 % | 9 | 100,0 % |
| 1 | 4,8 % | 0 | 0,0 % | 1 | 11,1 % |
| 15 | 71,4 % | 8 | 80,0 % | 6 | 66,7 % |
| 1 | 4,8 % | 0 | 0,0 % | 1 | 11,1 % |
| 2 | 9,5 % | 1 | 10,0 % | 0 | 0,0 % |
| 2 | 9,5 % | 1 | 10,0 % | 1 | 11,1 % |
